# Molecular Rearrangement of Pyrazino[2,3-*c*]quinolin-5(6*H*)-ones during Their Reaction with Isocyanic Acid

**DOI:** 10.3390/ijms23105481

**Published:** 2022-05-13

**Authors:** Antonín Klásek, Antonín Lyčka, Filip Křemen, Aleš Růžička, Michal Rouchal

**Affiliations:** 1 Department of Chemistry, Faculty of Technology, Tomas Bata University in Zlín, Vavrečkova 5669, 760 01 Zlín, Czech Republic; klasek@utb.cz (A.K.); kremen.filip@seznam.cz (F.K.); 2 Department of Chemistry, Faculty of Science, University of Hradec Králové, Rokitanského 62, 530 03 Hradec Králové, Czech Republic; antonin.lycka@vuos.com; 3 Department of General and Inorganic Chemistry, Faculty of Chemical Technology, University of Pardubice, Studentská 573, 532 10 Pardubice, Czech Republic; ales.ruzicka@upce.cz

**Keywords:** 3-(3-acylureido)-2,3-dihydro-1*H*-indol-2-ones, 4-alkylidene-1’*H*-spiro[imidazolidine-5,3’-indole]-2,2’-diones, imidazo[1,5-*c*]quinazoline-3,5-diones, ^1^H-, ^13^C- and ^15^N-NMR, scXRD, biological activity

## Abstract

New tetrahydropyrazino[2,3-*c*]quinolin-5(6*H*)-ones were prepared from 3-chloroquinoline-2,4(1*H*,3*H*)-diones and ethylene diamine. In their reaction with HNCO, an unprecedented molecular rearrangement produced new types of hydantoin derivatives. All prepared compounds were characterized on the basis of their ^1^H, ^13^C, and ^15^N NMR and ESI mass spectra and some were authenticated by X-ray analysis of single crystalline material. A proposed mechanism for rearrangement is discussed in this essay. The CDK and ABL inhibition activity as well as in vitro cytotoxicity of the prepared compounds was also tested.

## 1. Introduction

The presence of an amino group is common in many biologically active compounds. The group of reactive compounds including an amino group (especially α-aminoketones with respect to their easily conversion) belongs to various heterocycles [1]. Suitable compounds of this type are 3-aminoquinoline-2,4-diones, which is our particular area of interest [2,3,4,5,6,7,8,9,10,11,12,13,14,15]. Even if the occurrence of these compounds in the relevant literature was early rather than rare [16,17], we managed to prepare 3-aminoderivatives using 3-chloroquinolinediones and ammonium salts or primary amines [2]. Later, we proved that these compounds may also be prepared from 3-hydroxyquinoline-2,4-diones in reaction with ammonia or ammonium salts [14].

The biological activity of some 3-aminoquinoline-2,4-diones has been described [18]. 3-Amino-3-(4-fluorophenyl)-1*H*-quinoline-2,4-dione was demonstrated as effective against oxidative stress-related diseases [19] and suppresses reactive oxygen species [20,21]. A similar effect was exhibited by 3-amino-6-fluoro-3-(4-fluorophenyl)-1*H*-quinoline-2,4-dione [19].

We found that 3-aminoquinoline-2,4-diones were subject to molecular rearrangements after their reaction with urea [3,4,5], nitrourea [6,7], isocyanates [8], isothiocyanates [9,10,11], isothiocyanic acid [12,13,15], and isocyanic acid [13], creating a broad palette of new heterocyclic compounds, e.g., imidazo[1,5-*c*]quinazoline-3,5-diones, 3-(3-acylureido)-2,3-dihydro-1*H*-indol-2-ones, 4-alkylidene-1’*H*-spiro[imidazolidine-5,3’-indole]-2,2’-diones and spiro-linked imidazoline-2-thiones.

We also examined the reaction of 3-chloroquinolin-2,4-diones **1** with ethanolamine and found that the results were similar to those from the reaction of **1** with simple aliphatic amines, and 3-(2-hydroxyethylamino)quinoline-2,4-diones were obtained. Their reaction with isocyanic acid presented rearranged products that were structurally analogous to those listed above. However, their reaction with isothiocyanic acid proceeded differently and resulted in mainly non-rearranged compounds [15].

Considering these results, we decided to study the reactions of 3-chloroquinoline-2,4-diones **1** with 1,2-diamines. In the literature, most of the reactions reported are of α-haloketones with *o*-phenylenediamines. Surprisingly, reactions of tertiary α-bromoketones with aliphatic 1,2-diamines have only been described in one article [22].

In our previous paper [23], we described the reaction of N-1 unsubstituted 3-chloroquinolinediones with ethylene diamine. The results of this reaction were remarkable because we obtained two types of new quinazoline derivatives that did not react with isocyanic and isothiocyanic acids.

Hydantoin (systematically imidazolidine-2,4-dione) represents a structural motif that has been of interest to many researchers in recent years, not only chemically but also biologically [24,25,26,27]. Hydantoin-based compounds exhibit a broad range of biological activities, such as fungicidal, herbicidal, antitumor, anti-inflammatory, anti-HIV, hypolipidemic, antiarrhythmic, antiplatelet, and antihypertensive activities [28,29,30]. Some of these compounds have been approved for clinical use to treat many human diseases. For example, they act as muscle relaxants, anticonvulsants, or androgen receptor antagonists [28].

In this paper, we demonstrate that the reaction of ethylene diamine with N-1 substituted 3-chloroquinoline-2,4-diones proceeds smoothly without rearrangement to result in pyrazino[2,3-*c*]quinolin-5(6*H*)-ones **2**. Moreover, new molecular rearrangement of the easily obtainable compounds **2** yielded two hitherto unknown types of potentially biologically active hydantoins during their reaction with isothiocyanic acid.

## 2. Results and Discussion

**Scheme 1:**
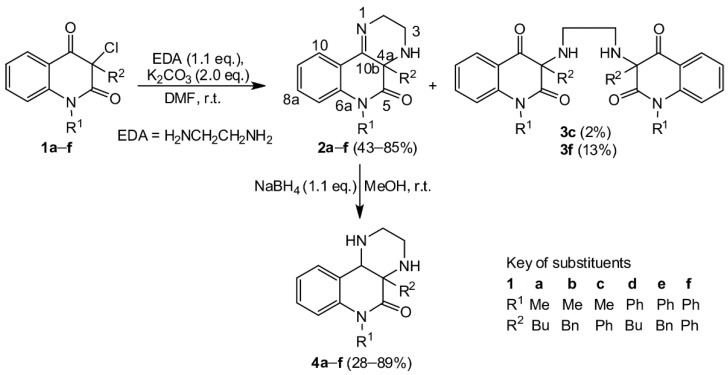
The preparation and reduction of pyrazino [2,3-*c*]quinolin-5(6*H*)-ones **2**.

**Scheme 2:**
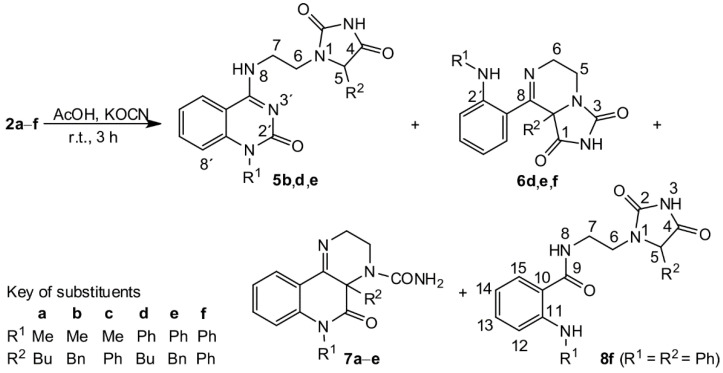
Reaction of compounds **2** with potassium cyanate.

**Figure 1:**
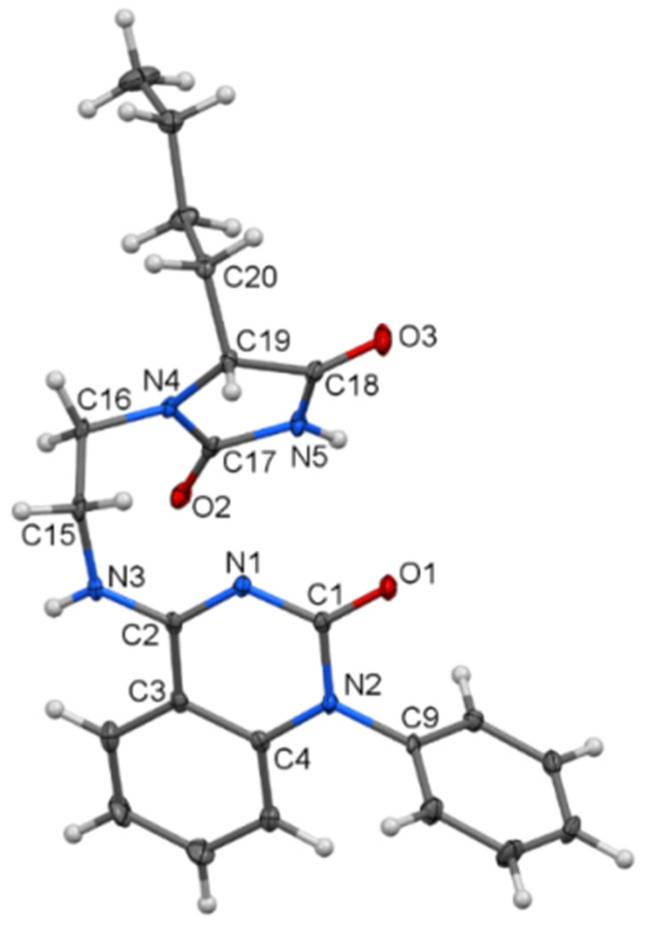
Molecular structure of compound **5d**—*ORTEP* diagram drawn with 40% probability level.

Our purpose was to study in detail the reaction connected with the isolation of a large quantity of minority compounds and to clarify the reaction mechanism. The reactions of 3-chloroquinolin-2,4-diones **1a–f** with ethylene diamine were performed in DMF in the presence of powdered potassium carbonate. In a good yield, novel tricyclic pyrazino[2,3-*c*]quinolin-5(6*H*)-ones **2** were obtained (Scheme 1). In just two cases, a small quantity of dimeric compounds **3c** and **3f** was produced via double alkylation of ethylene diamine with the chloroderivatives **1c** and **1f**. Their ^1^H and ^13^C NMR spectra exhibited two sets of signals according to the presence of two observable diastereoisomers. Reaction of compounds **2** with sodium borohydride confirmed the presence of the imine group and led to the expected dihydroderivatives **4** (Scheme 1). Even though ethylene diamine is a strong base, we did not observe the formation of other compounds that would be products of a rearrangement analogous to rearrangement of 3-aminoquinolinediones. The NMR spectra and chemical shifts for the isolated compounds **2**, **3**, and **4** are presented in the Supplementary Materials (see Figures S1–S15 and Tables S1–S3, respectively). The reactions of compound **2** with potassium cyanate were carried out with a molar ratio 1:1.6 in a solution of acetic acid (Scheme 2, Table 1). Our first look at the IR and NMR spectra for the reaction products showed that at least three types of compounds were present. However, we were not able to determine the structure of the isolated compounds from their NMR spectra. Only a few isolated fragments were found, but it was impossible to determine how they were interconnected. Fortunately, after more unsuccessful experimentation, we managed to prepare a single crystal of the compound acquired from compound **2d**. The structure of this compound (**5d**) was established by X-ray diffraction analysis (Figure 1). Although the structures of imidazolidine-2,4-dione (also a part of **5d** skeleton) derivatives had been described crystallographically more than 170 times, derivatives with a longer hydrocarbon chain are absent from the literature. Moreover, the second part of the **5d** molecule, a 1,2-dihydroquinazolin-2-one fragment, is scarcely reported [31,32].

In **5d** (Figure 1), the planes of the imidazolidine-2,4-dione and 1,2-dihydroquinazolin-2-one parts, which are separated by an iminoethane bridge, exhibit an interplanar angle of 26.16(9)°. The two molecules are interconnected by C=O···H–N bridges (see Supplementary Materials, Figure S29).

The structure of **5d** is surprising because its creation requires the scission of the C(2)–C(3) bond in the starting compound **2d**. We did not observe such a reaction at any time. The transformation of quinolinedione to a quinazolinedione skeleton was previously observed only in cases where the starting compound was N-unsubstituted, allowing the formation of a useful isocyanate intermediate [23].

Compound **5d** consists of two bioactive moieties: 4-iminoquinazolin-2-one and substituted hydantoin. Several methods for the preparation of closely related quinazolin-4-ones [33] and quinazoline-2,4-diones [34] were recently described; however, none of them are remotely similar to the presented transformation. It must be pointed out that the reaction of compounds **2** with HNCO was carried out with a molar ratio 1:1.6 because we did not anticipate initially the reaction of compound **2** with more than one mole of isocyanic acid. Therefore, complete conversion of compounds **2** to **5** cannot be expected, but rather, only the formation of a mixture of products can proceed (Table 1). Using an excess of KNCO, the composition of the reaction products changed (Table 1), but at no time was the full conversion of **2** to **5** achieved.

Compounds **5b** and **5e** belong to the group of compounds produced by the reaction of **2** with two equivalents of HNCO that exhibited an absorption band at ca. 1770 cm^−1^ in the IR spectrum characteristic of hydantoins [35]. All their NMR data (see Supplementary Materials, Table S4 and Figures S16 and S18, respectively) are in the agreement with the proposed structure.

In addition to compound **5d**, the next product was obtained from compound **2d**. From ESI-MS and elemental analysis, it was determined that only one mole of HNCO was consumed. Its IR spectrum exhibited an absorption band at 1776 cm^–1^, indicative of the presence of a hydantoin ring [14], and a singlet at 11.2 ppm appeared in the ^1^H NMR spectrum pertaining to a NH proton in position 2 of the hydantoin moiety [36]. The fragment Ar―NH―Ph was also found, which bears witness to the opening of the quinolinone ring in **2d**.

**Figure 2:**
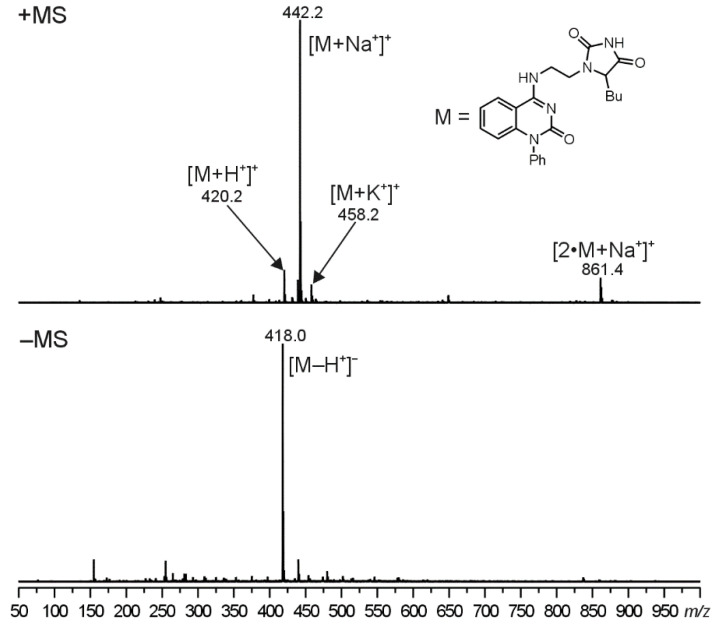
The first-order positive and negative ion ESI-MS spectra for compound **5d**. The assignments for the observed signals are shown in the brackets.

The molecular structure of compounds **5** were proved using ESI-MS/MS analyses. In the positive-ion first-order mass spectra, four singly charged ions were observed. The most abundant ion, assigned as a sodium adduct of the molecule ([M+Na^+^]^+^), was accompanied by two less intense signals at *m*/*z* corresponding to a protonated molecule ([M+H^+^]^+^) and a potassium adduct of the molecule ([M+K^+^]^+^). Moreover, a sodium adduct of the dimer ([2 M+Na^+^]^+^) was observed in the case of compounds **5**. In the negative polarity mode, an ion assigned as a deprotonated molecule ([M+Na^+^]^+^) was formed. Illustrative ESI mass spectra for compound **5d** can be seen in Figure 2 (ESI-MS spectra for compounds **5b** and **5e** are given in the Supplementary Materials, Figures S47 and S48, respectively).

Compound **6d** represents the second structural group of products produced from the reaction of **2** with only one mole of isocyanic acid that exhibited an IR absorption band at *ca* 1760 cm^–1^. Compounds **6e** and **6f** also pertain to this group. All these compounds display an absorption band at *ca* 1760 cm^–1^ in the IR spectrum and a broad signal at *ca* 11.1 ppm in their ^1^H NMR spectra. In their ^13^C NMR spectra (see Supplementary Materials, Table S5 and Figures S19–S21, respectively), quaternary carbons signals appeared at *ca* 68.9 ppm and, in their ^15^N NMR spectra, a signal adherent to the C=N group can be seen, much like that for the starting compound **2**. Four nitrogen atoms were present in forenamed compounds. One belonged to a C=N group, the second was imidic, and the third pertained to a tertiary amino group. Therefore, the fourth nitrogen atom, which exhibited a singlet at *ca* 8 ppm in its ^1^H NMR spectrum, must be part of Ar―NH―R^1^ grouping. In both positive and negative ion ESI-MS spectra for compounds **6**, the most abundant signal was observed at *m/z* corresponding to a (de)protonated molecule (see Supplementary Materials, Figures S49–S51).

The third product of the reaction of **2d** with HCNO was a compound that did not have any IR absorption around 1760 cm^–1^ and, therefore, did not contain a hydantoin ring. Its ^1^H and ^13^C NMR spectra (see Supplementary Materials, Figure S25) were similar to **2d**, but the presence of a CONH_2_ group in the results suggest the structure of **7d**. The reaction of this compound with an excess of HNCO (Table 1) provided compound **5d**, indicating that **7d** is the first intermediate in the molecular rearrangement of **2d**. It was found that compounds **7** resulted from all compounds **2** except **2f**.

**Figure 3:**
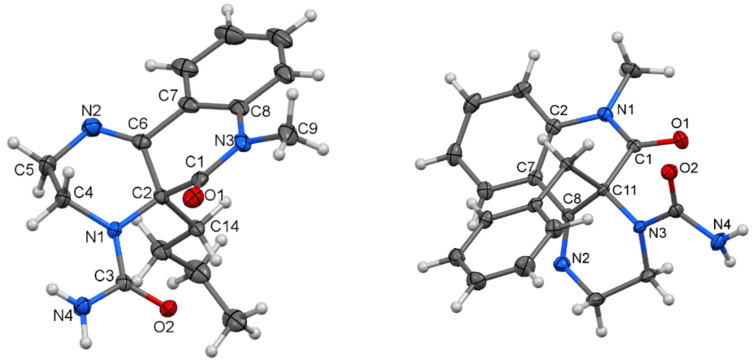
Molecular structure of compound **7a** (**left**) and **7b** (**right**)—*ORTEP* diagrams drawn with 40% probability level.

The molecular structure of compounds **7a** (Figure 3, left) and **7b** (Figure 3, right) were proven by X-ray analysis. The structures of **7a** and **7b** are characterized by the presence of substituted tricyclic systems where the π―electron conjugation is interrupted by the presence of a stereogenic center at C-2 (**7a**) and or C-11 (**7b**) as well as an ethylene bridge. The constitution of the tricyclic system in **7a** is totally unknown. On the other hand, the characteristic interatomic distances and angles in both compounds that crystallize in achiral space groups *P*2_1_/*c* and *P*-1, respectively, are essentially the same as previously known structures with the same type of functional groups and atom hybridization [37,38].

Three molecules of **7a** co-crystallize with two molecules of water to form an extensive system of H-bridges. In **7b**, both optical isomers are interconnected by an NH···O=C bridging motif. Co-crystallized dichloromethane molecules occupy tunnels formed by the aromatic rings of the molecule. All the geometric parameters for all X-rayed structures are given in the Supplementary Materials (Figures S28–S33, Tables S8–S16). Compounds **7c** and **7d** exhibited anomalous behavior in the form of very broad signals when their NMR spectra were measured in DMSO-*d*_6_. Therefore, they were measured in CDCl_3_.

As in the case of the above-mentioned compounds, the structures of compounds **7** were confirmed using mass spectrometry. Except commonly observed ions, such as protonated molecules, sodium and potassium adducts of the molecule, and/or sodium adducts of the dimer, we observed a singly charged signal in the positive-ion first-order ESI mass spectra that was assigned as a [M+H^+^–HCNO]^+^ ion. Its presence can be explained, according to tandem mass spectrometry experiments, as a result of in-source fragmentation. ESI mass spectra for compounds **7** are given in the Supplementary Materials (see Figures S52–S56).

**Scheme 3:**
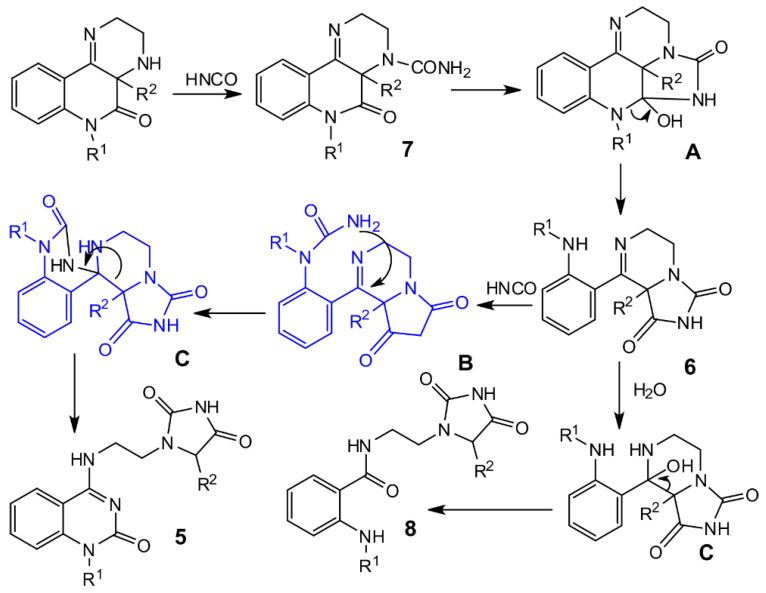
Proposed reaction mechanism.

Compound **7** is primarily the product of the reaction between compound **2** and isocyanic acid, and therefore provides the starting compounds for the following molecular rearrangement to compounds **5** and **6**. Our proposal for the reaction mechanism for rearrangement of compounds **2** is illustrated in Scheme 3. We suppose that addition of compound **2** to isocyanic acid produces compound **7,** which is subsequently changed to compound **6** via the intermediate **A**. The reaction of compound **6** with isocyanic acid affords the intermediate **B**, which undergoes retro-Claisen condensation for the formation of compounds **5**.

One of the isolated products, prepared from **2f**, was different from the compounds mentioned above. The fragment NCH_2_CH_2_N was present, but the compound did not contain the C=N group, and instead of a quaternary carbon atom, it contained a CHR group. The presence of an IR band at 1775 cm^–1^ in the IR spectrum and 11.2 ppm in the ^1^H NMR spectrum indicated that the hydantoin ring must be present. In the molecule that pointed to the structure **8f**, the amide group was found (see Supplementary Materials, Table S7 and Figure S27). Not only IR and NMR, but also mass spectrometry provided clear evidence for the structure of compound **8f**. Results for its ESI-MS analysis are given in Supplementary Materials (see Figure S57). The origin of this compound can be explained by the addition of water to compound **6f** and following retro-Claisen condensation through intermediates **B** and **C** (Scheme 3).

As mentioned in the introduction, some compounds bearing a quinoline or hydantoin moiety are known to possess a wide range of biological activities. However, there are only few examples of compounds possessing both of the above-mentioned structural motifs. For example, Kumar and co-workers published the synthesis of new series of 7-chloroquinoline-thiohydantoin derivatives with potent antimalarial activity [39]. Quinoline and hydantoin derivatives are well-known for their anticancer activity, as recently described in several comprehensive reviews [28,40]. According to this fact, we decided to test the antiproliferative activity of compounds **5**, **6**, and **7** on two types of human tumor cell lines (K-562, chronic myelogenous leukemia and MV4;11, acute myelogenous leukemia). Moreover, the inhibitory potency of these compounds was assayed on two types of protein kinases, namely the recombinant heterodimeric complex CDK2/cyclin E and tyrosine-protein kinase ABL1. Unfortunately, no biological activity was observed for concentrations up to 10 µM.

## 3. Materials and Methods

### 3.1. General Data

Melting points were determined with a Kofler block. IR (KBr) spectra were recorded with a Smart OMNI-Transmission Nicolet iS10 spectrophotometer. The ^1^H, ^13^C, and ^15^N NMR spectra were recorded with a Bruker Avance III HD 500 spectrometer (500.13 MHz for ^1^H, 125.76 MHz for ^13^C, and 50.68 MHz for ^15^N) in DMSO-*d*_6_. ^1^H and ^13^C chemical shifts are given on the *δ* scale (ppm) and are referenced to internal TMS (*δ* = 0.0). ^15^N chemical shifts were referred to external neat CH_3_NO_2_ in a co-axial capillary (*δ* = 0.0). All 2D experiments (gradient-selected (gs)-COSY, gs-TOCSY, gs-HMQC, gs-HMQC-RELAY, gs-HMBC) were performed using the manufacturer’s software. Full-sets of diffraction data for **5d** (yellow) and **7a** and **7b** (colorless) were collected at 150(2)K with a D8-Venture diffractometer (Bruker, Germany) equipped with Cu (Cu/K_α_ radiation; λ = 1.54178 Å) or Mo (Mo/K_α_ radiation; λ = 0.71073 Å) microfocus X-ray (IµS) sources, CMOS photon detector, and an Oxford Cryosystems cooling device was used for data collection. Experimental details are stated in Supporting Information. The frames were integrated with the Bruker SAINT Software package using a narrow-frame algorithm. Data were corrected for absorption effects using the Multi-Scan method (SADABS). The obtained data were treated by XT-version 2018/1 and SHELXL-2017/1 software implemented in an APEX3 v2016.5-0 (Bruker AXS) system [41]. The positive-ion EI mass spectra were measured on a QP-2010 instrument (Shimadzu, Japan) within the mass range *m*/*z* = 50–600 using a direct inlet probe (DI). Samples were dissolved in dichloromethane (30 μg·mL^–1^) and 10 μL of the solution was evaporated in a DI cuvette at 50 °C. The ion source temperature was 200 °C; the energy of electrons was 70 eV. Only signals exceeding a relative abundance of 5% are listed. The electrospray mass spectra (ESI-MS) were recorded using an amaZon X ion-trap mass spectrometer (Bruker Daltonics, Bremen, Germany) equipped with an electrospray ion source. All experiments were conducted in both positive and negative polarity mode. Individual samples (with a concentration of 500 ng·mL^–1^) were infused into the ESI source as methanol/water (1/1, *v*/*v*) solutions via a syringe pump with a constant flow rate of 3 μL·min^–1^. The other instrumental conditions were as follows: *m*/*z* range 50–1500, electrospray voltage of –4.2 kV (4.2 kV in negative polarity mode), capillary exit voltage of 140 V (–140 V in negative polarity mode), drying gas temperature of 220 °C, drying gas flow of 6.0 dm^3^·min^–1^, nebulizer pressure of 55.16 kPa. Nitrogen was used as the nebulizing and drying gases for all experiments. Tandem mass spectra were collected using collision-induced dissociation (CID) with He as the collision gas after isolating the required ions. Column chromatography was carried out on silica gel (Merck, grade 60, 70–230 mesh) using successive mixtures of chloroform/ethanol (in ratios from 99:1 to 8:2) (S1) or benzene/ethyl acetate (in ratios from 99:1 to 8:2) (S2). Reactions, the course of separation, and the purity of substances were monitored by TLC (elution systems: benzene/ethyl acetate (4:1) (S3), chloroform/ethanol (9:1 and 1:1) (S4 and S5), and chloroform/ethyl acetate (7:3) (S6)) on Alugram^®^ SIL G/UV_254_ foils (Macherey-Nagel, Germany). Elemental analyses (C, H, N) were performed with an EA Flash EA 1112 Elemental Analyzer (Thermo Fisher Scientific, Waltham, MA, USA).

### 3.2. General Procedure for the Reaction of Compounds **1** with Ethylene Diamine

To the solution of compound **1** (1 mmol) in DMF (9 mL), pulverized potassium carbonate (276 mg, 2 mmol) and ethylene diamine (EDA) (0.1 mL, 1.1 mmol) were added and the mixture was stirred at room temperature. The course of the reaction was monitored with TLC. After the spot corresponding to compound **1** faded away, the reaction mixture was diluted with water (20 mL). The deposited product was filtered with suction, dried and crystallized with an appropriate solvent. In cases where the crude product was oily or waxy, the solution was extracted with chloroform (3 × 20 mL). The collected extracts were dried, evaporated to dryness, and the residue was separated by chromatography on a silica gel column.

4a-Butyl-6-methyl-2,3,4,4a-tetrahydro-pyrazino[2,3-*c*]quinolin-5(6*H*)-one (**2a**)

Compound was prepared from **1a** and EDA with 53% yield, reaction time 6 h. White solid, mp 111–113 °C (ethyl acetate/hexane). ^1^H and ^13^C chemical shifts in DMSO-d_6_: C(2)H_2_ 50.2/3.82 and 3.49, C(3)H_2_ 39.2/2.90 and 2.64, C(4a) 63.2, C(5) 171.7, C(6a) 139.1, C(7)H 114.9/7.21, C(8)H 131.4/7.49, C(9)H 123.3/7.18, C(10)H 125.1/7.65, C(10a) 123.9, C(10b) 162.7, C(1’(R^1^))H_3_ 30.1/3.33, C(1’(R^2^))H_2_ 39.0/1.45 and 1.31, C(2’(R^2^))H_2_ 25.4/1.31 and 1.05, C(3’(R^2^))H_2_ 22.0/1.05, C(4’(R^2^))H_3_ 13.8/0.69 ppm. IR (cm^–1^) ν: 3346, 3042, 2959, 2936, 2872, 2825, 1676, 1645, 1602, 1462, 1431, 1410, 1367, 1347, 1315, 1297, 1279, 1265, 1229, 1192, 1125, 1109, 1056, 1044, 992, 961, 946, 840, 759, 740, 680, 654,627, 579, 532. ESI-MS (pos.) *m*/*z* (%): 565.2 [2·M + Na^+^]^+^ (7), 294.1 [M + K^+^]^+^ (19), 272.1 [M + Na^+^]^+^ (100), 216.0 [M + H^+^]^+^ (11). Anal. Calcd for C_16_H_21_N_3_O (271.36): C 70.82; H 7.80; N 15.49. Found: C 70.55; H 8.00; N 15.40.

6-Methyl-4a-benzyl-2,3,4,4a-tetrahydropyrazino[2,3-*c*]quinolin-5(6*H*)-one (**2b**)

Compound was prepared from **1b** and EDA with 43% yield. Colorless solid, mp 102–106 °C (benzene/hexane). ^1^H and ^13^C chemical shifts in DMSO-d_6_: C(2)H_2_ 49.8/3.64 and 3.02, C(3)H_2_ 38.8/2.88 and 2.45, C(4a) 64.1, C(5) 170.8, C(6a) 139.1, C(7)H 115.1/7.0,2 C(8)H 131.6/7.24, C(9)H 123.3/7.19, C(10)H 125.3/7.64, C(10a) 123.8, C(10b) 161.7, C(1’(R^1^))H_3_ 30.1/3.35, C(1’(R^2^))H_2_ 44.9/2.75 and 2.68, C(2’(R^2^)) 135.2, C(3’(R^2^))H 130.4/6.94, C(4’(R^2^))H 127.4/7.16, C(5’(R^2^))H 126.8/7.19 ppm. ^15^N chemical shifts and ^1^J(^15^N, ^1^H) coupling constants in DMSO-d_6_: N(1) −52.7, N(4) −351.7, N(6) −257.6 ppm. IR (cm^–1^) ν: 3329, 3066, 3028, 2942, 2905, 2838, 1668, 1633, 1603, 1497, 1472, 1455, 1438, 1416, 1357, 1339, 1298, 1270, 1226, 1202, 1159, 1129, 1076, 1062, 1047, 1010, 958, 903, 763, 699, 656, 620, 535, 504. ESI-MS (pos.) *m*/*z* (%): 633.2 [2·M + Na^+^]^+^ (6), 328.0 [M + Na^+^]^+^ (20), 306.0 [M + H^+^]^+^ (100), 214.9 [M + H^+^ − C_7_H_7_]^+^ (10). Anal. Calcd for C_19_H_19_N_3_O (305.37): C 74.73; H 6.27; N 13.76. Found: C 74.63; H 6.40; N 13.84.

6-Methyl-4a-phenyl-2,3,4,4a-tetrahydropyrazino[2,3-*c*]quinolin-5(6*H*)-one (**2c**)

Compound was prepared from **1c** with 54% yield beside **3c**. White solid, mp 178–182 °C (benzene). ^1^H and ^13^C chemical shifts in DMSO-d_6_: C(2)H_2_ 49.9/3.96 and 3.81, C(3)H_2_ 37.8/2.71 and 2.65, C(4a) 65.6, C(5) 169.2, C(6a) 138.8, C(7)H 115.1/7.12, C(8)H 131.5/7.43, C(9)H 123.4/7.14, C(10)H 125.1/7.81, C(10a) 124.2, C(10b) 160.6, C(1’(R^1^))H_3_ 30.1/3.39, C(1’(R^2^)) 139.9, C(2’(R^2^))H 126.9/7.18, C(3’(R^2^)) 128.5/7.29, C(4’(R^2^))H 127.9/7.24 ppm. IR (cm^–1^) ν: 3358, 2933, 2838, 1671, 1638, 1602, 1469, 1446, 1417, 1362, 1297, 1150, 1124, 1080, 991, 896, 845, 763, 744, 706, 692, 661, 625, 569, 536, 506. EI-MS *m*/*z* (%): 292 (21), 291 (M^+^, 100), 290 (20), 262 (23), 261 (47), 160 (12), 132 (14), 131 (20), 104 (18), 77 (16). ESI-MS (pos.) *m*/*z* (%): 605.2 [2·M + Na^+^]^+^ (42), 583.2 [2·M + H^+^]^+^ (16), 314.1 [M + Na^+^]^+^ (18), 292.1 [M + H^+^]^+^ (100). Anal. Calcd for C_18_H_17_N_3_O (291.35): C 74.20; H 5.88; N 14.42. Found: C 73.99; H 5.98; N 14.24.

4a-Butyl-6-phenyl-2,3,4,4a-tetrahydropyrazino[2,3-*c*]quinolin-5(6*H*)-one (**2d**)

Compound was prepared from **1d** and EDA with 85% yield. Colorless solid, mp 86–90 °C (hexane). ^1^H and ^13^C chemical shifts in DMSO-d_6_: C(2)H_2_ 50.2/3.82 and 3.49, C(3)H_2_ 39.2/2.90 and 2.64, C(4a) 63.2, C(5) 171.7, C(6a) 139.1, C(7)H 114.9/7.21, C(8)H 131.4/7.49, C(9)H 123.3/7.18, C(10)H 125.1/7.65, C(10a) 123.9, C(10b) 162.7, C(1’(R^1^)) 139.1, C(2’(R^1^))H overlap/7.25, C(3’(R^1^))H 130.2/7.57, C(4’(R^1^))H 128.7/7.50, C(1’(R^2^))H_2_ 39.0/1.73 and 1.59, C(2’(R^2^))H_2_ 25.4/1.38 and 1.15, C(3’(R^2^))H_2_ 22.0/1.15, C(4’(R^2^))H_3_ 13.9/0.77 ppm. IR (cm^–1^) ν: 3448, 3330, 2954, 2868, 1677, 1642, 1604, 1492, 1456, 1348, 1332, 1314, 1293, 1261, 1222, 1177, 1113, 1072, 999, 770, 697, 682, 656, 648, 610, 491. EI-MS: *m*/*z* (%) 334 (6), 333 (24), 291 (6), 290 (25), 277 (21), 276 (100), 275 (7), 262 (6), 77 (7), 57 (5). ESI-MS (pos.) *m*/*z* (%): 356.1 [M + Na^+^]^+^ (5), 334.2 [M + H^+^]^+^ (100), 278.1 [M + H^+^ – C_4_H_8_]^+^ (3). Anal. Calcd for C_21_H_23_N_3_O (333.43): C 75.65; H 6.95; N 12.60. Found: C 75.82; H 7.14; N 12.55.

4a-Benzyl-6-phenyl-2,3,4,4a-tetrahydropyrazino[2,3-c]quinolin-5(6*H*)-one (**2e**)

Compound was prepared from **1e** and EDA with 51% yield, reaction time 3 h. White solid, mp 161–164 °C (benzene/cyclohexane). ^1^H and ^13^C chemical shifts in DMSO-d_6_: C(2)H_2_ 49.8/3.73 and 3.10, C(3)H_2_ 38.6/2.95 and 2.56, C(4a) 64.4, C(5) 170.9, C(6a) 140.0, C(7)H 116.2/6.34, C(8)H 131.2/7.35, C(9)H 123.4/7.13, C810)H 125.6/7.73, C(10a) 123.4, C(10b) 161.5, C(1’(R^1^)) 137.9, C(2’(R^1^))H overlapped signals, C(3’(R^1^))H 130.1/7.52, C(4’(R^1^))H 128.7/7.51, C(1’(R^2^))H_2_ 44.7/3.06 and 2.91, C(2’(R^2^)) 135.2, C(3’(R^2^))H 130.6/7.10, C(4’(R^2^))H 127.5/7.22, C(5’(R^2^))H 126.9/7.22 ppm. IR (cm^–1^) ν: 3354, 2925, 2897, 2836, 1690, 1638, 1600, 1492, 1460, 1351, 1315, 1275, 1214, 1164, 1073, 1003, 959, 877, 782, 766, 741, 702, 657, 632, 598. EI-MS: *m*/*z* (%): 367 (14), 277 (21), 276 (100), 91 (8), 77 (5). ESI-MS (pos.) *m*/*z* (%): 757.3 [2·M + Na^+^]^+^ (5), 390.1 [M + Na^+^]^+^ (21), 368.1 [M + H^+^]^+^ (100), 277.0 [M + H^+^ – C_7_H_7_]^+^ (5). Anal. Calcd for C_24_H_21_N_3_O (367.44): C 78.45; H 5.76; N 11.44. Found: C 78.41; H 5.88; N 11.43.

4a,6-Diphenyl-2,3,4,4a-tetrahydropyrazino[2,3-*c*]quinolin-5(6*H*)-one (**2f**)

Compound was prepared from **1f** and EDA with 66% yield. Colorless solid, mp 156–160 °C (hexane). ^1^H and ^13^C chemical shifts in DMSO-d_6_: C(2)H_2_ 49.9/3.97 and 3.94, C(3)H_2_ 37.5/2.72 and 2.60, C(4a) 66.1, C(5) 169.4, C(6a) 139.7, C(7)H 116.9/6.09, C(8)H 131.2/7.16, C(9)H 123.5/7.08, C(10)H 125.5/7.83, C(10a) 123.7, C(10b) 160.2, C(1’(R^1^)) 137.8, C(2’(R^1^))H overlapped signals, C(3’(R^1^))H 130.3/7.56, C(4’(R^1^))H 129.5/7.52, C(1’(R^2^)) 139.4, C(2’(R^2^))H 127.0/7.26, C(3’(R^2^))H 129.5/7.35 C(4’(R^2^))H 128.7/7.28 ppm. ^15^N chemical shifts and ^1^J(^15^N, ^1^H) coupling constants in DMSO-d_6:_ N(1) −53.0, N(4) −339.3, N(6) −234.3 ppm. IR (cm^–1^) ν: 3442, 2908, 2820, 1681, 1638, 1602, 1493, 1458, 1422, 1355, 1335, 1261, 1261, 1160, 1109, 995, 936, 893, 758, 740, 719, 700, 663, 627, 574, 516. EI-MS: *m*/*z* (%): 354 (26), 353 (100), 352 (16), 338 (6), 324 (13), 323 (26), 296 (5), 250 (7), 249 (5), 248 (5), 222 (7), 221 (9), 194 (14), 193 (6), 149 (6), 131 (8), 104 (11), 103 (6), 77 (17), 71 (5), 66 (5), 57 (9), 55 (6), 51 (7), 43 (10). ESI-MS (pos.) *m*/*z* (%): 354.1 [M + H^+^]^+^ (100). Anal. Calcd for C_23_H_19_N_3_O (353.42): C 78.16; H 5.42; N 11.89: Found: C 78.06; H 5.50; N 11.88.

3,3’-(Ethane-1,2-diyl)bis(azanediyl)bis(1-methyl-3-phenylquinoline)-2,4(1*H*,3*H*)-dione (**3c**)

Compound was prepared from **1c** with 2% yield beside **2c**. Yellowish solid, mp 198–210 °C (benzene-hexane). ^1^H and ^13^C chemical shifts in DMSO-d_6_: C(2) 171.32 and 171.23, C(3) 76.98 and 76.96, C(4) 193.27 and 192.72, C(4a) 120.71 and 120.60, C(5)H 127.48 and 127.39/7.75, C(6)H 123.29 and 123.27/7.17, C(7)H 136.41 and 136.39/7.69, C(8)H 115.99 and 115.96/7.39, C(8a) 142.3, NH 2.60, CH_2_ 45.38 and 45.17/2.59 and 2.48, C(1’(R^1^)) 139.63 and 139.43 C(1’(R^2^)) 137.78 and 137.44, other C/H signals exist as broadened overlapped signals resonating at 126.9–131.2/7.12–7.8 ppm. IR (cm^–1^) ν: 3333, 3064, 3033, 2946, 2854, 1703, 1666, 1602, 1472, 1417, 1354, 1303, 1254, 1185, 1114, 1034, 994, 911, 864, 764, 699, 684, 637, 600, 533, 495. ESI-MS (pos.) *m*/*z* (%): 581.2 [M + Na^+^]^+^ (44), 559.2 [M + H^+^]^+^ (100). ESI-MS (neg.) *m*/*z* (%): 575.1 [M – H^+^ + H_2_O]^–^ (100), 557.1 [M – H^+^]^–^ (37). Anal. Calcd for C_34_H_30_N_4_O_4_ (558.63): C 73.10; H 5.41; N 10.03. Found: C 73.41; H 5.68; N 9.95.

3,3’-(Ethane-1,2-diyl)bis(azanediyl)bis(1,3-diphenylquinoline)-2,4(1*H*,3*H*)-dione (**3f**)

Compound was prepared from **1f** with 13% yield. Colorless solid, mp 262–268 °C (benzene/hexane). ^1^H and ^13^C chemical shifts in DMSO-d_6_: C(2) 171.33 and 171.26, C(3) 77.23 and 77.19, C(4) 193.10 and 192.64, C(4a) 120.50 and 120.44, C(6)H 123.48 and 123.40, C(7)H 135.93/7.46, C(8)H 116.59 and 116.55/6.32, C(8a) 142.3, N 2.60, CH_2_ 45.33 and 45.17/2.58 and 2.51, C(1’(R^1^))H_3_ 30.03 and 30.00/3.53 and 3.50, C(1’(R^2^)) 137.91 and 137.86, C(2’(R^2^))H 126.69 and 126.64/7.31, C(3’(R^2^))H 128.84 and 128.79/7.28 C(4’(R^2^))H 128.66 and 128.62/7.28 ppm. IR (cm^–1^) ν: 3442, 3063, 2927, 2858, 1707, 1673, 1600, 1492, 1461, 1337, 1303, 1249, 1192, 1173, 1158, 1113, 1072, 1031, 1002, 981, 902, 820, 762, 747, 719, 703, 650, 609, 576, 539, 516. ESI-MS (pos.) *m*/*z* (%): 1387.5 [2·M + Na^+^]^+^ (11), 1365.4 [2·M + H^+^]^+^ (6), 721.2 [M + K^+^]^+^ (7), 705.3 [M + Na^+^]^+^ (31), 683.3 [M + H^+^]^+^ (100) Anal. Calcd for C_44_H_34_N_4_O_4_ (682.77): C 77.40; H 5.02; N 8.21. Found: C 76.98; H 5.13; N 8.36.

### 3.3. General Procedure for the Reduction of Compounds 2 with NaBH_4_

To the solution of compound **2** (1.5 mmol) in methanol (20 mL), NaBH_4_ (67 mg, 1.7 mmol) was added over 5 min. The mixture was stirred for 1.5–3 h at room temperature and then poured onto 20 mL of crushed ice. Hydrochloric acid (35%, 0.28 mL) was added, and after 5 min, 5% NaHCO_3_. The alkaline reaction mixture was extracted with chloroform (3 × 25 mL), dried and evaporated to dryness. The residue was crystallized with an appropriate solvent.

4a-Butyl-6-methyl-1,2,3,4,4a,10b-hexahydropyrazino[2,3-*c*]quinolin-5(6*H*)-one (**4a**)

Compound was prepared from **2a** with 28% yield. Colorless solid, mp 145–149 °C (hexane). ^1^H and ^13^C chemical shifts in DMSO-d_6_: C(2)H_2_ 45.4/2.90 and 2.68, C(3)H_2_ 39.9/2.65 and 2.61, C(4a) 56.9, C(5) 171.6, C(6a) 138.1, C(7)H 114.1/7.06, C(8)H 127.6/7.27, C(9)H 123.4/7.27, C(10)H 122.7/7.06, C(10a) 127.4, C(10b)H 58.2/3.87, C(1’(R^1^))H_3_ 29.3/3.24, C(2’(R^1^))H 129.2/7.29, C(3’(R^1^))H 130.1/7.50, C(4’(R^1^))H 129.0/7.50, C(1’(R^2^))H_2_ 22.6/1.96 and 0.57, C(2’(R^2^))H_2_ 24.2/1.14 and 0.86, C(3’(R^2^))H_2_ 22.4/1.05, C(4’(R^2^))H_3_ 14.0/0.73 ppm. IR (cm^–1^) ν: 3369, 3064, 3040, 2951, 2928, 2862, 2801, 1666, 1601, 1497, 1470, 1443, 1418, 1357, 1294, 1275, 1233, 1203, 1156, 1124, 1040, 983, 958, 887, 874, 847, 824, 758, 684, 632, 593, 548, 537. ESI-MS (pos.) *m*/*z* (%): 547.2 [2·M + H^+^]^+^ (7), 274.1 [M + H^+^]^+^ (100). Anal. Calcd for C_16_H_23_N_3_O (273.37): C 70.30; H 8.48; N 15.37. Found: C 70.53; H 8.34; N 15.23.

4a-Benzyl-6-methyl-1,2,3,4,4a,10b-hexahydropyrazino[2,3-*c*]quinolin-5(6*H*)-one (**4b**)

Compound was prepared from **2b** with 30% yield. Colorless solid, mp 207–210 °C (ethanol). ^1^H and ^13^C chemical shifts in DMSO-d_6_: C(2)H_2_ 45.5/3.11 and 3.03, C(3)H_2_ 40.1/2.77 and 2.71, C(4a) 58.6, C(5) 170.4, C(6a) 138.2, C(7)H 114.3/7.13, C(8)H 127.8/7.37, C(9)H 123.5/7.32, C(10)H 122.8/7.13, C(10a) 127.3, C(10b)H 58.4/3.98, C(1’(R^1^))H_3_ 29.3/3.24, C(1’(R^2^))H_2_ 29.7/3.21 and 2.04, C(2’(R^2^)) 136.7, C(3’(R^2^))H 129.7/6.80, C(4’(R^2^))H 127.9/7.15, C(5’(R^2^))H 126.1/7.15 ppm. IR (cm^–1^) ν: 3295, 3070, 3024, 2969, 2940, 2898, 2835, 2814, 2769, 2721, 1665, 1604, 1504, 1479, 1470, 1459, 1422, 1367, 1336, 1318, 1284, 1238, 1145, 1132, 1118, 1082, 1050, 991, 973, 862, 827, 764, 730, 702, 691, 662, 643, 504. ESI-MS (pos.) *m*/*z* (%): 637.2 [2·M + Na^+^]^+^ (4), 330.1 [M + Na^+^]^+^ (9), 308.1 [M + H^+^]^+^ (100). Anal. Calcd for C_19_H_21_N_3_O (307.39): C 74.69; H 6.89; N 13.69. Found: C 74.57; H 7.05; N 13.61.

6-Methyl-4a-phenyl-1,2,3,4,4a,10b-hexahydropyrazino[2,3-*c*]quinolin-5(6*H*)-one (**4c**)

Compound was prepared from **2c** with 89% yield. Colorless solid, mp 216–218 °C (benzene). ^1^H and ^13^C chemical shifts in DMSO-d_6_: C(2)H_2_ 46.1/2.99 and 2.87, C(3)H_2_ 40.4/2.60 and 2.32, C(4a) 59.7, C(5) 170.4, C(6a) 138.0, C(7)H 114.7/6.95, C(8)H 127.4/7.20, C(9)H 123.1/7.13, C(10)H 122.8/7.49, C(10a) 12714, C(10b)H 58.0/4.25, C(1’(R^1^))H_3_ 29.8/3.23, C(1’(R^2^)) 137.4, C(2’(R^2^))H 129.1/7.460.86, C(3’(R^2^)) 127.3/7.05, C(4’(R^2^))H 126.7/7.05 ppm. IR (cm^–1^) ν: 3067, 2954, 2922, 2802, 1668, 1601, 1495, 1470, 1448, 1412, 1355, 1306, 1271, 1155, 1140, 1117, 1042, 981, 951, 816, 771, 756, 719, 705, 679, 694, 600, 543. ESI-MS (pos.) *m*/*z* (%): 587.2 [2·M + H^+^]^+^ (7), 316.0 [M + Na^+^]^+^ (8), 294.1 [M + H^+^]^+^ (100). Anal. Calcd for C_18_H_19_N_3_O (293.36): C 73.69; H 6.53; N 14.32. Found: C 73.60; H 6.70; N 14.32.

4a-Butyl-6-phenyl-1,2,3,4,4a,10b-hexahydropyrazino[2,3-*c*]quinolin-5(6*H*)-one (**4d**)

Compound was prepared from **2d** with 62% yield. Colorless solid, mp 120–124 °C (benzene). ^1^H and ^13^C chemical shifts in DMSO-d_6_: C(2)H_2_ 45.4/2.96 and 2.74, C(3)H_2_ 39.7/2.73 and 2.66, C(4a) 57.5, C(5) 171.8, C(6a) 138.6, C(7)H 115.5/6.15, C(8)H 127.4/7.07, C(9)H 123.9/7.04, C(10)H 123.0/7.44, C(10a) 127.1, C(10b)H 58.2/4.15, C(1’(R^1^)) 139.2, C(2’(R^1^))H 129.2/7.15, C(3’(R^1^))H 130.0/7.54, C(4’(R^1^))H 128.2/7.44, C(1’(R^2^))H_2_ 22.7/2.08 and 0.89, C(2’(R^2^))H_2_ 24.3/1.26 and 1.02, C(3’(R^2^))H_2_ 22.5/1.18, C(4’(R^2^))H_3_ 14.1/0.80 ppm. ^15^N chemical shifts and ^1^J(^15^N, ^1^H) coupling constants in DMSO-d_6:_ N(1) −344.6, N(4) −354.2, N(6) −235.0 ppm. IR (cm^–1^) ν: 3251, 3206, 3064, 2958, 2932, 2872, 1709, 1666, 1604, 1494, 1461, 1405, 1379, 1353, 1300, 1266, 1201, 1158, 1141, 1105, 1048, 929, 872, 838, 757, 696, 667, 564. ESI-MS (pos.) *m*/*z* (%): 671.3 [2·M + H^+^]^+^ (11), 358.1 [M + Na^+^]^+^ (5), 336.1 [M + H^+^]^+^ (100). Anal. Calcd for C_20_H_21_N_3_O (335.40): C 71.62; H 6.31; N 12.53. Found: C 71.79; H 6.48; N 12.43.

4a-Benzyl-6-phenyl-1,2,3,4,4a,10b-hexahydropyrazino[2,3-*c*]quinolin-5(6*H*)-one (**4e**)

Compound was prepared from **2e** with 80% yield. Colorless solid, mp 182–184 °C (benzene/hexane) ^1^H and ^13^C chemical shifts in DMSO-d_6_: C(2)H_2_ 45.5/3.08 and 2.82, C(3)H_2_ 40.4/3.15 and 2.74, C(4a) 58.8, C(5) 170.4, C(6a) 138.5, C(7)H 116.1/6.29, C(8)H 127.5/7.14, C(9)H 123.8/7.14, C(10)H 123.1/7.44, C(10a) 127.4, C(10b)H 58.6/4.27, C(1’(R^1^)) 139.2, C(2’(R^1^))H 129.1/7.16, C(3’(R^1^))H 129.8/7.52, C(4’(R^1^))H 128.0/7.43, C(1’(R^2^))H_2_ 29.8/3.36 and 2.27, C(2’(R^2^)) 136.7, C(3’(R^2^))H 129.9/7.04, C(4’(R^2^))H 128.1/7.26, C(5’(R^2^))H 126.2/7.16 ppm. IR (cm^–1^) ν: 3287, 3268, 3059, 3019, 2913, 2851, 1690, 1603, 1489, 1449, 1347, 1335, 1289, 1277, 1233, 1193, 1153, 1123, 1080, 1029, 897, 951, 899, 874, 755, 724, 695, 639, 595, 556. ESI-MS (pos.) *m*/*z* (%): 761.3 [2·M + Na^+^]^+^ (5), 739.3 [2·M + H^+^]^+^ (18), 392.1 [M + Na^+^]^+^ (10), 370.1 [M + H^+^]^+^ (100). Anal. Calcd for C_24_H_23_N_3_O (369.46): C 78.02; H 6.27; N 11.37. Found: C 77.97; H 6.25; N 11.28.

4a,6-Diphenyl-1,2,3,4,4a,10b-hexahydropyrazino[2,3-*c*]quinolin-5(6*H*)-one (**4f**)

Compound was prepared from **2f** with 80% yield. Colorless solid, mp 174–179 °C (benzene). ^1^H and ^13^C chemical shifts in DMSO-d_6_: C(2)H_2_ 45.5/3.09 and 2.96, C(3)H_2_ 40.1/2.70 and 2.43, C(4a) 60.3, C(5) 170.2, C(6a) 138.2, C(7)H 115.8/6.01, C(8)H 127.1/6.98, C(9)H 123.3/7.11, C(10)H 123.2/7.53, C(10a) 127.8, C(10b)H 57.5/4.55, C(1’(R^1^)) 138.2, C(2’(R^1^))H 128.7/7.08, C(3’(R^1^))H 129.9/7.52, C(4’(R^1^))H 128.2/7.43, C(1’(R^2^)) 136.9, C(2’(R^2^))H 129.3/7.64, C(3’(R^2^))H 127.5/7.16, C(4’(R^2^))H 127.1/7.16 ppm. IR (cm^–1^) ν: 3261, 2943, 2904, 2851, 1674, 1604, 1467, 1452, 1346, 1291, 1144, 1072, 772, 755, 717, 698, 648, 586, 550. ESI-MS (pos.) *m*/*z* (%): 356.2 [M + H^+^]^+^ (100). Anal. Calcd for C_23_H_21_N_3_O (355.43): C 77.72; H 5.96; N 11.82. Found: C 77.59; H 6.00; N 11.69.

### 3.4. General Procedure for the Reaction of Compounds 2 with Isocyanic Acid

To the solution of **2** (1.5 mmol) in acetic acid (4.5 mL), potassium cyanate (0.195 g, 2.4 mmol) was added, and the mixture was stirred for 3 h at room temperature. The mixture was poured onto crushed ice (20 mL) and extracted with chloroform (5 × 15 mL). The collected extracts were dried and evaporated to dryness. The residue was chromatographed on a silica gel column.

5-Benzyl-1-{2-[(1-methyl-2-oxo-2,3-dihydroquinazolin-4(1*H*)-ylidene)amino]ethyl}imida-zolidine-2,4-dione (**5b**)

Compound was prepared from **2b** with 18% yield beside **7b**. Colorless solid, mp 209–215 °C (ethyl acetate). ^1^H and ^13^C chemical shifts in DMSO-d_6_: C(2) 156.5, C(4) 173.8, C(5)H 60.6/4.55, C(6)H_2_ 38.7/3.61, C(7)H_2_ 38.1/3.85 and 3.17, C(2’) 155.2, C(4’) 160.0, C(4a’) 109.8, C(5’)H 123.7/7.95, C(6’)H 121.0/7.20, C(7’)H 133.9/7.67, C(8’)H 114.4/7.34, C(8a’) 142.8, C(1’(R^1^))H_3_ 30.0/3.43, C(2’(R^1^))H_3_ 30.0/3.43 C(3’(R^1^))H_3_ 30.0/3.43 C(4’(R^1^))H_3,_ 30.0/3.43, C(1’(R^2^))H_2_ 33.4/3.14 and 3.01, C(2’(R^2^)) 135.4, C(3’(R^2^))H 128.2/7.10, C(4’(R^2^))H 129.4/7.20, C(5’(R^2^))H 126.7/7.20 ppm. ^15^N chemical shifts and ^1^J(^15^N, ^1^H) coupling constants in DMSO-d_6:_ N(1) −286.8, N(3)H n.o./10.53 ^1^J(^15^N, ^1^H) 95.2 Hz, N(8)H −286.8/8.37 ^1^J(^15^N, ^1^H) 88.5 Hz, N(1’) −262.9, N(3’) −171.2 ppm. IR (cm^–1^) ν: 3400, 3129, 3030, 2939, 2746, 1761, 1709, 1619, 1597, 1565, 1543, 1497, 1456, 1419, 1352, 1329, 1263, 1234, 1173, 1138, 1127, 1095, 1036, 1005, 946, 872, 851, 768, 749, 702, 681, 650, 621, 594, 535. ESI-MS (pos.) *m*/*z* (%): 805.3 [2·M + Na^+^]^+^ (8), 430.1 [M + K^+^]^+^ (5), 414.1 [M + Na^+^]^+^ (100), 392.1 [M + H^+^]^+^ (22). ESI-MS (neg.) *m*/*z* (%): 390.0 [M – H^+^]^–^ (100). Anal. Calcd for C_21_H_21_N_5_O_3_ (391.16): C 64.44; H 5.41; N 17.89. Found: C 64.63; H 5.70; N 17.89.

5-Butyl-1-{2-[(2-oxo-1-phenyl-2,3-dihydroquinazolin-4(1*H*)-ylidene)amino]ethyl}imidazolidine-2,4-dione (**5d**)

Compound was prepared from **2d** in 17% yield beside **6d** and **7d**. Yellowish solid, mp 247–250 °C (benzene/cyclohexane). ^1^H and ^13^C chemical shifts in DMSO-d_6_: C(2) 156.9, C(4) 174.6, C(5)H 59.9/4.26, C(6)H_2_ 38.8/3.67, C(7)H_2_ 38.6/3.82 and 3.24, C(2’) 154.6, C(4’) 160.6, C(4a’) 109.4, C(5’)H 123.7/8.04, C(6’)H 121.4/7.19, C(7’)H 133.6/7.45, C(8’)H 115.0/6.40, C(8a’) 147.7, C(1’(R^1^)) 138.2, C(2’(R^1^))H 128.3/7.26 C(3’(R^1^))H 129.9/7.58, C(4’(R^1^))H 128.3/7.49, C(1’(R^2^))H_2_ 27.4/1.76 and 1.73, C(2’(R^2^))H_2_ 135.4, C(3’(R^2^)) H_2_ 21.9/1.20, C(4’(R^2^)) H_3_ 13.8/0.79 ppm. ^15^N chemical shifts and ^1^J(^15^N, ^1^H) coupling constants in DMSO-d_6:_ N(1) −286.4, N(3)H n.o./10.78 ^1^J(^15^N, ^1^H) 90.8 Hz, N(8)H −285.0/8.58 ^1^J(^15^N, ^1^H) 92.4 Hz, N(1’) −241.2, N(3’) −171.4 ppm. IR (cm^–1^) ν: 3335, 3063, 2956, 2871, 1771, 1703, 1640, 1599, 1565, 1537, 1492, 1453, 1418, 1390, 1354, 1327, 1263, 1230, 1183, 1156, 1138, 1113, 1086, 1070, 973, 877, 813, 764, 748, 704, 675, 654, 613, 562, 547, 510. ESI-MS (pos.) *m*/*z* (%): 861.4 [2·M + Na^+^]^+^ (8), 458.2 [M + K^+^]^+^ (6), 442.2 [M + Na^+^]^+^ (100), 420.2 [M + H^+^]^+^ (11). ESI-MS (neg.) *m*/*z* (%): 418.0 [M – H^+^]^–^ (100). Anal. Calcd for C_23_H_25_N_5_O_3_ (419.48): C 65.85; H 6.01; N 16.70. Found: C 65.74; H 6.07; N 16.57. Using the excess of KOCN (3 equiv.), 17% of **5d**, 8% of **6d** and 31% of **7d** was obtained. Using the excess of KOCN (3 equiv.), compound **5d** was prepared in 57% yield from **6d** and in 33% yield from **7d**.

5-Benzyl-1-{2-[(2’-oxo-1’-phenyl-1,2-dihydroquinazolin-4-yl)amino]ethyl}imidazolidine-2,4-dione (**5e**)

Compound was prepared from **2e** with 22% yield beside **6e** and **7e**. Colorless solid, mp 181–192 °C (ethyl acetate). After recrystallization from ethanol, the mp increased to 280–283 °C without any change in its IR spectrum. ^1^H and ^13^C chemical shifts in DMSO-d_6_: C(2) 156.6, C(4) 173.8, C(5)H 60.6/4.60, C(6)H_2_ 38.8/3.69, C(7)H_2_ 38.2/3.82 and 3.20, C(2’) 154.5, C(4’) 160.6, C(4a’) 109.4, C(5’)H 123.6/8.02, C(6’)H 121.3/7.21, C(7’)H 133.5/7.49, C(8’)H 115.0/6.41, C(8a’) 143.7, C(1’(R^1^)) 138.2, C(2’(R^1^))H 128.2/7.24, C(3’(R^1^))H 129.9/7.59, C(4’(R^1^))H 128.2/7.50, C(1’(R^2^))H_2_ 30.9/3.20 and 3.05, C(2’(R^2^)) 135.4, C(3’(R^2^))H 128.2/7.14, C(4’(R^2^))H 129.4/7.28, C(5’(R^2^))H 126.7/7.22 ppm. ^15^N chemical shifts and ^1^J(^15^N, ^1^H) coupling constants in DMSO-d_6:_ N(1) −287.6, N(3)H −232.2/10.58 ^1^J(^15^N, ^1^H) 94.6 Hz, N(8)H −285.0/8.56 ^1^J(^15^N, ^1^H) 92.7 Hz, N(1’) −241.1, N(3’) −171.7 ppm. IR (cm^–1^) ν: 3331, 3065, 2936, 1770, 1712, 1653, 1641, 1615, 1600, 1538, 1488, 1454, 1423, 1355, 1330, 1225, 1184, 1156, 1131, 1084, 1030, 775, 753, 707, 675, 622, 541, 509. ESI-MS (pos.) *m*/*z* (%): 929.4 [2·M + Na^+^]^+^ (4), 492.2 [M + K^+^]^+^ (11), 476.2 [M + Na^+^]^+^ (100), 454.2 [M + H^+^]^+^ (16). ESI-MS (neg.) *m*/*z* (%): 452.0 [M – H^+^]^–^ (100). Anal. Calcd for C_26_H_23_N_5_O_3_ (453.49): C 68.86; H 5.11; N 15.44. Found: C 68.67; H 5.56; N 15.22. Using the excess of KOCN (4 equiv.), 50% of **5e**, 6% of **6e** and 29% of **7e** was prepared from **2e**.

8a-Butyl-8-[2-(phenylamino)phenyl]-5,6-dihydroimidazo[1,5-*a*]pyrazine-1,3(2*H*,8a*H*)-dione (**6d**)

Compound was prepared from **2d** with 13% yield. Colorless solid, mp 187–190 °C (benzene). ^1^H and ^13^C chemical shifts in DMSO-d_6_: C(1) 170.6, C(3) 156.2, C(5)H_2_ 46.4/3.82, C(6)H_2_ 33.9/3.82 and 3.15, C(8) 163.1, C(8a) 68.0, C(1’) 128.3, C(2’) 141.7, C(3’)H 117.9/7.10, C(4’)H 128.7/7.19, C(5’)H 119.7/6.86, C(6’)H 129.3/7.10, C(1’(R^1^)) 143.6, C(2’(R^1^))H 118.6/7.02, C(3’(R^1^))H 128.7/7.19, C(4’(R^1^))H 120.3/6.83, C(1’(R^2^))H_2_ 32.7/1.94, C(2’(R^2^))H_2_ 24.7/1.15 and 0.98, C(3’(R^2^)) H_2_ 21.7/1.15, C(4’(R^2^)) H_3_ 13.7/0.74 ppm. ^15^N chemical shifts and ^1^J(^15^N, ^1^H) coupling constants in DMSO-d_6:_ N(2)H n.o./11.01, N(4)H −290.8, N(7) −53.7, N(2’)H −295.9/7.15 ^1^J(^15^N, ^1^H) 90.3 Hz. IR (cm^–1^) ν: 3302, 3046, 2958, 2871, 2732, 1776, 1719, 1640, 1593, 1508, 1455, 1419, 1303, 1127, 1115, 1070, 1021, 912, 890, 859, 756, 699, 678, 627, 578, 534, 499. ESI-MS (pos.) *m*/*z* (%): 399.2 [M + Na^+^]^+^ (12), 377.2 [M + H^+^]^+^ (100). ESI-MS (neg.) *m*/*z* (%): 375.0 [M – H^+^]^–^ (100). Anal. Calcd for C_22_H_24_N_4_O_2_ (376.45): C 70.19; H 6.43; N 14.88. Found: C 70.30; H 6.58; N 14.53.

8a-Benzyl-8-[2-(phenylamino)phenyl]-5,6-dihydroimidazo[1,5-*a*]pyrazine-1,3(2*H*,8a*H*)-dione (**6e**)

Compound was prepared from **2e** with 14% yield. Colorless solid, mp 227–230 °C (benzene). ^1^H and ^13^C chemical shifts in DMSO-d_6_: C(1) 169.9, C(3) 155.8, C(5)H_2_ 46.6/3.93 and 3.86, C(6)H_2_ 34.0/3.78 and 3.42, C(8) 162.9, C(8a) 68.3, C(1’) 128.2, C(2’) 142.1, C(3’)H 117.7/7.12, C(4’)H 128.9/7.22, C(5’)H 120.1/6.93, C(6’)H 129.4/7.22,C(1’(R^1^)) 143.5, C(2’(R^1^))H 119.1/7.02, C(3’(R^1^))H 128.7/7.21, C(4’(R^1^))H 120.5/6.83, C(1’(R^2^))H_2_ 38.5/3.26 and 3.36, C(2’(R^2^)) 133.8, C(3’(R^2^))H 131.2/7.02, C(4’(R^2^))H 128.3/7.20, C(5’(R^2^))H 127.1/7.22 ppm. ^15^N chemical shifts and ^1^J(^15^N, ^1^H) coupling constants in DMSO-d_6:_ N(2)H n.o./10.68, N(4)H −291.4, N(7) −50.6, N(2’)H −295.6/7.37 ^1^J(^15^N, ^1^H) 90.4 Hz. IR (cm^–1^) ν: 3323, 3032, 2932, 2731, 1770, 1720, 1645, 1595, 1510, 1496, 1478, 1454, 1411, 1302, 1137, 1067, 1039, 929, 753, 700, 689, 663, 597, 560, 535, 491. ESI-MS (pos.) *m*/*z* (%): 433.2 [M + Na^+^]^+^ (7), 411.2 [M + H^+^]^+^ (100). ESI-MS (neg.) *m*/*z* (%): 409.0 [M – H^+^]^–^ (100). Anal. Calcd for C_25_H_22_N_4_O_2_ (410.47): C 73.15; H 5.40; N 13.65. Found: C 73.12; H 5.55; N 13.81. Using an excess of KOCN (3 equiv.), 6% of **6e**, 29% of **7e**, and 30% of **5e** was obtained.

8a-Phenyl-8-[2-(phenylamino)phenyl]-5,6-dihydroimidazo[1,5-*a*]pyrazine-1,3(2*H*,8a*H*)-dione (**6f**)

Compound was prepared from **2f** and KOCN (3 equiv.) with 20% yield beside **8f**. Colorless solid, mp 190–192 °C (ethyl acetate/hexane). ^1^H and ^13^C chemical shifts in DMSO-d_6_: C(1) 170.3, C(3) 155.4, C(5)H_2_ 45.5/4.03 and 3.55, C(6)H_2_ 36.2/3.48 and 3.34, C(8) 165.0, C(8a) 68.9, C(1’) 125.3, C(2’) 138.8, C(3’)H 118.9/7.19, C(4’)H 130.8/7.23, C(5’)H 119.4/6.82, C(6’)H 131.6/7.25,C(1’(R^1^)) 142.8, C(2’(R^1^))H 118.9/7.01, C(3’(R^1^))H 128.9/7.22, C(4’(R^1^))H 120.9/6.89, C(1’(R^2^)) 137.3, C(2’(R^2^))H 126.5/7.39, C(3’(R^2^))H 128.9/7.42, C(4’(R^2^))H 130.0/7.42 ppm. ^15^N chemical shifts and ^1^J(^15^N, ^1^H) coupling constants in DMSO-d_6:_ N(2)H n.o./11.05, N(4)H −285.5, N(7) −58.2, N(2’)H −292.2/8.85 ^1^J(^15^N, ^1^H) 87.2 Hz. IR (cm^–1^) ν: 3181, 3061, 2925, 2849, 2740, 1775, 1718, 1594, 1570, 1497, 1451, 1310, 1220, 1167, 1124, 1070, 1031, 963, 913, 889, 853, 751, 696, 594, 549. ESI-MS (pos.) *m*/*z* (%): 815.2 [2·M + Na^+^]^+^ (4), 419.1 [M + Na^+^]^+^ (18), 397.1 [M + H^+^]^+^ (100). ESI-MS (neg.) *m*/*z* (%): 813.2 [2·M – 2·H + Na^+^]^−^ (33), 395.0 [M – H^+^]^–^ (100). Anal. Calcd for C_24_H_20_N_4_O_2_ (396.43): C 72.71; H 5.08; N 14.13. Found: C 72.29; H 5.06; N 14.08.

4a-Butyl-6-methyl-5-oxo-2,3,5,6-tetrahydropyrazino[2,3-*c*]quinoline-4(4a*H*)-carboxamide (**7a**)

Compound was prepared from **2a** and KOCN (1.4 equiv.) with 47% yield. Using an excess of KNCO (4 equiv.), **7a** was prepared with 57% yield. Colorless solid, mp 155–158 °C (ethyl acetate/hexane). ^1^H and ^13^C chemical shifts in DMSO-d_6_: C(2)H_2_ n.o/3.94, C(3)H_2_ 40.1/3.80 and 3.49, CO 159.9, C(4a) n.o., C(5) 168.5, C(6a) 139.3, C(7)H 114.8/7.20, C(8)H 131.7/7.51, C(9)H 122.8/7.13, C(10)H 125.2/7.74, C(10a) 124.6, C(10b) 162.5, C(1’(R^1^))H_3_ 30.3/3.31, C(1’(R^2^))H_2_ 30.3/1.58, C(2’(R^2^))H_2_ 25.2/1.19 and 0.85, C(3’(R^2^))H_2_ 21.7/1.03, C(4’(R^2^))H_3_ 13.7/0.66 ppm. IR (cm^–1^) ν: 3350. 3193, 2954, 2856, 1684, 1644, 1621, 1603, 1461, 1402, 1358, 1310, 1255, 1223, 1138, 1059, 1041, 1008, 992, 966, 941, 862, 750, 727, 699, 676, 595, 559. ESI-MS (pos.) *m*/*z* (%): 651.3 [2·M + Na^+^]^+^ (19), 353.0 [M + K^+^]^+^ (6), 337.1 [M + Na^+^]^+^ (77), 315.1 [M + H^+^]^+^ (100), 272.0 [M + H^+^ – HCNO]^+^ (16). ESI-MS (neg.) *m*/*z* (%): 312.9 [M – H^+^]^–^ (100). Anal. Calcd for C_17_H_22_N_4_O_2_ (314.38): C 64.95; H 7.05; N 17.82. Found: C 64.75; H 7.22; N 17.71.

4a-Benzyl-6-methyl-5-oxo-2,3,5,6-tetrahydropyrazino[2,3-*c*]quinolone-4-(4a*H*)-carbox- amide (**7b**)

Compound was prepared from **2b** with 63% yield beside **5b**. Colorless solid, mp 146–151 °C (ethyl acetate/hexane). ^1^H and ^13^C chemical shifts in DMSO-d_6_: C(2)H_2_ 48.6/3.66, C(3)H_2_ 40.4/3.61 and 2.74, CO 160.3, C(4a) 69.2, C(5) 167.8, C(6a) 139.6, C(7)H 115.1/7.20, C(8)H 131.9/7.53, C(9)H 123.0/7.20, C(10)H 125.9/7.75, C(10a) 122.9, C(10b) 161.1 C(1’(R^1^))H_3_ 30.7/3.35, C(1’(R^2^))H_2_ 39.7/2.60 and 2.32, C(2’(R^2^)) 135.4, C(3’(R^2^))H 129.8/6.91, C(4’(R^2^))H 127.5/7.20, C(5’(R^2^))H 129.8/7.20 ppm. IR (cm^–1^) ν: 3447, 2942, 1686, 1663, 1647, 1602, 1493, 1470, 1430, 1360, 1297, 1270, 1224, 1138, 1059, 1039, 1011, 973, 951, 919, 875, 760, 704, 620, 556, 504. ESI-MS (pos.) *m*/*z* (%): 719.3 [2·M + Na^+^]^+^ (18), 387.1 [M + K^+^]^+^ (12), 371.1 [M + Na^+^]^+^ (85), 349.1 [M + H^+^]^+^ (100), 306.1 [M + H^+^ – HCNO]^+^ (32). Anal. Calcd for C 68.95; H 5.73; N 16.08. Found: C 68.81; H 5.92; N 15.82.

6-Methyl-5-oxo-4a-phenyl-2,3,5,6-tetrahydropyrazino[2,3-*c*]quinoline-4(4a*H*)-carbox- amide (**7c**)

Compound was prepared from **2c** with 32% yield. Using the excess of KOCN (4 equiv.), **7c** was prepared in 68% yield. Yellowish solid, mp 209–211 °C (chloroform). ^1^H and ^13^C chemical shifts in DMSO-d_6_: C(2)H_2_ 50.4/4.21, C(3)H_2_ 40.1/3.48 and 2.77, CO 160.7, C(4a) 68.8, C(5) 171.5, C(6a) 137.5, C(7)H 116.4/6.42, C(8)H 131.4/7.27, C(9)H 124.2/7.17, C(10)H 126.2/7.96, C(10a) 123.1, C(10b) 161.3, C(1’(R^1^)) 139.2, C(2’(R^1^))H 129.2/7.29, C(3’(R^1^))H 130.1/7.50, C(4’(R^1^))H 129.0/7.50, C(1’(R^2^))H_2_ 34.8/3.19 and 1.95, C(2’(R^2^))H_2_ 25.2/1.36 and 1.06, C(3’(R^2^))H_2_ 22.1/1.21, C(4’(R^2^))H_3_ 13.7/0.81 ppm. ^15^N chemical shifts and ^1^J(^15^N, ^1^H) coupling constants in DMSO-d_6:_ N(1) −54.1, N(4) −296.1, NH_2_ −301.9/5.21 ^1^J(^15^N, ^1^H) 83.6 Hz. IR (cm^–1^) ν: 3422, 3352, 3305, 3247, 3197, 3062, 2953, 1689, 1663, 1626, 1601, 1471, 1406, 1361, 1297, 1170, 1127, 1079, 1029, 1010, 933, 903, 886, 821, 762, 696, 633, 554. ESI-MS (pos.) *m*/*z* (%): 691.2 [2·M + Na^+^]^+^ (9), 373.0 [M + K^+^]^+^ (17), 357.0 [M + Na^+^]^+^ (100), 335.1 [M + H^+^]^+^ (84), 292.0 [M + H^+^ – HCNO]^+^ (13). Anal. Calcd for C_19_H_18_N_4_O_2_ (334.37): C 68.25; H 5.43; N 16.76. Found: C 68.21; H 5.50; N 16.79.

4a-Butyl-5-oxo-6-phenyl-2,3,5,6-tetrahydropyrazino[2,3-*c*]quinoline-4(4a*H*)-carboxamide(**7d**)

Compound was prepared from **2d** with 38% yield beside **5d** and **6d**. Colorless solid, mp 144–152 °C (benzene/hexane). ^1^H and ^13^C chemical shifts in DMSO-d_6_: C(2)H_2_ 50.4/4.21, C(3)H_2_ 40.1/3.48 and 2.77, CO 160.7, C(4a) 68.8, C(5) 171.5, C(6a) 137.5, C(7)H 116.4/6.42, C(8)H 131.4/7.27, C(9)H 124.2/7.17, C(10)H 126.2/7.96, C(10a) 123.1, C(10b) 161.3, C(1’(R^1^)) 139.2, C(2’(R^1^))H 129.2/7.29, C(3’(R^1^))H 130.1/7.50, C(4’(R^1^))H 129.0/7.50, C(1’(R^2^))H_2_ 34.8/3.19 and 1.95, C(2’(R^2^))H_2_ 25.2/1.36 and 1.06, C(3’(R^2^))H_2_ 22.1/1.21, C(4’(R^2^))H_3_ 13.7/0.81 ppm. ^15^N chemical shifts and ^1^J(^15^N, ^1^H) coupling constants in DMSO-d_6:_ N(1) −54.1, N(4) −296.1, NH_2_ −301.9/5.21 ^1^J(^15^N, ^1^H) 83.6 Hz. IR (cm^–1^) ν: 3434, 3398, 3215, 2955, 2851, 1700, 1662, 1645, 1607, 1489, 1459, 1428, 1350, 1330, 1313, 1301, 1257, 1217, 1173, 1163, 1131, 1052, 1030, 1009, 955, 877, 801, 769, 756, 701, 679, 646, 603, 570, 511, 490. ESI-MS (pos.) *m*/*z* (%): 775.4 [2·M + Na^+^]^+^ (8), 415.2 [M + K^+^]^+^ (10), 399.2 [M + Na^+^]^+^ (84), 377.2 [M + H^+^]^+^ (100). ESI-MS (neg.) *m*/*z* (%): 375.0 [M – H^+^]^–^ (100). Anal. Calcd for C_22_H_24_N_4_O_2_ (376.45): C 70.19; H 6.43; N 14.89. Found: C 70.51; H 6.41; N 14.52.

4a-Benzyl-5-oxo-6-phenyl-2,3,5,6-tetrahydropyrazino[2,3-*c*]quinoline-4(4a*H*)-carbox- amide (**7e**)

Compound was prepared from **2e** with 49% yield beside **5e** and **6e**. Colorless solid, mp 197–200 °C (benzene/cyclohexane). ^1^H and ^13^C chemical shifts in DMSO-d_6_: C(2)H_2_ 48.5/3.66, C(3)H_2_ 40.4/3.74 and 2.74, CO 160.3, C(4a) 69.2, C(5) 167.7, C(6a) 138.4, C(7)H 115.9/6.24, C(8)H 131.5/7.32, C(9)H 123.0/7.19, C(10)H 127.0/7.84, C(10a) 122.5, C(10b) 161.4, C(1’(R^1^)) 139.2, C(2’(R^1^))H 129.2 and 128.2/7.19, C(3’(R^1^))H 130.1/7.56, C(4’(R^1^))H 129.0/7.50, C(1’(R^2^))H_2_ 34.8/3.19 and 1.95, C(2’(R^2^))H_2_ 25.2/1.36 and 1.06, C(3’(R^2^))H_2_ 22.1/1.21, C(4’(R^2^))H_3_ 13.7/0.81 ppm. IR (cm^–1^) ν: 3427, 3196, 3063, 2938, 2850, 1703, 1666, 1602, 1492, 1460, 1420, 1359, 1336, 1310, 1298, 1267, 1217, 1179, 1074, 1046, 1032, 877, 845, 792, 754, 704, 641, 606, 575, 560, 544, 497. ESI-MS (pos.) *m*/*z* (%): 843.4 [2·M + Na^+^]^+^ (6), 449.1 [M + K^+^]^+^ (16), 433.2 [M + Na^+^]^+^ (85), 411.2 [M + H^+^]^+^ (100), 368.2 [M + H^+^ – HCNO]^+^ (6). ESI-MS (neg.) *m*/*z* (%): 409.0 [M – H^+^]^–^ (100). Anal. Calcd for C_25_H_22_N_4_O_2_ (410.46): C 73.15; H 5.40; N 13.65. Found: C 73.28; H 5.92; N 13.51.

*N*-[2-(2,4-Dioxo-5-phenylimidazolidin-1-yl)ethyl]-2-(phenylamino)benzamide (**8f**)

Compound was prepared from **2f** with 18% yield beside **6f**. Colorless solid, mp 160–165 °C (benzene). ^1^H and ^13^C chemical shifts in DMSO-d_6_: C(2) 158.8, C(4) 172.9, C(5)H_2_ 64.3/2.22, C(6)H_2_ 36.6/3.38 and 3.31, C(7)H_2_ 39.6/3.77, C(9) 169.1, C(10) 119.0, C(11) 144.0, C(12)H 114.9/7.29, C(13)H 131.9/7.32, C(14)H 118.1/6.83, C(15) 128.9/7.57, C(1’(R^2^)) 141.5, C(2’(R^2^))H 119.4/7.15, C(3’(R^2^))H 129.4/7.30, C(4’(R^2^))H 121.7/6.97, C(1’(R^2^)) 133.8, C(2’(R^2^))H 127.6/7.16, C(3’(R^2^))H 129.1/7.36, C(4’(R^2^))H 128.8/7.36 ppm. ^15^N chemical shifts and ^1^J(^15^N, ^1^H) coupling constants in DMSO-d_6:_ N(1) −284.5, N(3)H −235.5./11.03 ^1^J(^15^N, ^1^H) 94.6 Hz, N(8)H −269.0/9.44 ^1^J(^15^N, ^1^H) 91.5 Hz, N(11)H −292.2/8.68 ^1^J(^15^N, ^1^H) 89.5 Hz. IR (cm^–1^) ν: 3390, 3353, 3228, 3068, 2939, 2740, 1775, 1722, 1629, 1589, 1512, 1447, 1421, 1383, 1329, 1310, 1282, 1222, 1167, 1157, 1120, 1078, 1053, 1027, 963, 942, 895, 841, 751, 701, 628, 580, 516. ESI-MS (pos.) *m*/*z* (%): 851.3 [2·M + Na^+^]^+^ (11), 453.2 [M + K^+^]^+^ (26), 437.2 [M + Na^+^]^+^ (100), 415.2 [M + H^+^]^+^ (31). ESI-MS (neg.) *m*/*z* (%): 413.0 [M – H^+^]^–^ (100). Anal. Calcd for C_24_H_22_N_4_O_3_ (414.46): C 69.55; H 5.35; N 13.52. Found: C 69.99; H 5.78; N 13.17.

### 3.5. CDK and ABL Inhibition Assay

CDK2/cyclin E and ABL1 activity was assayed as previously described [42,43]. Briefly, the kinase was assayed with [γ-^33^P]ATP and suitable peptide substrates in a reaction buffer (60 mM HEPES-NaOH, pH 7.5, 3 mM MgCl_2_, 3 mM MnCl_2_, 3 μM Na-orthovanadate, 1.2 mM DTT, 2.5 μg/50 μL PEG_20.000_). The reactions were stopped by adding 5 µL of 3% aq. H_3_PO_4_. Aliquots were spotted onto P-81 phosphocellulose, washed with 0.5% aq. H_3_PO_4_ and air-dried. Kinase inhibition was quantified using an FLA-7000 digital image analyzer. The concentration of the test compound required to reduce kinase activity by 50% was determined from a dose-response curves and reported as the IC_50_ value.

### 3.6. In Vitro Cytotoxicity

Cell lines K562 and MV4;11 were obtained from the European Collection of Cell Cultures. The cell lines were cultivated in Dulbecco’s Modified Eagle medium supplemented with 10% fetal bovine serum, penicillin (100 U/mL), and streptomycin (100 μg/mL) at 37 °C in 5% CO_2_. For the viability assays, cells were seeded into 96-well plates (5000 cells per well), and after the preincubation period, were treated in triplicate with six different doses of each compound for 72 h. After treatment, a resazurin (Sigma-Aldrich) solution was added for four hours, and the fluorescence of resorufin formed in live cells was measured at 544 nm/590 nm (excitation/emission) using a Fluoroskan Ascent microplate reader (Labsystems). The IC_50_ value, the drug concentration that was lethal for 50% of the cells, was calculated from the dose–response curve.

## 4. Conclusions

In conclusion, the tetrahydropyrazino[2,3-*c*]quinolin-5(6*H*)-ones **2** react with isocyanic acid to give (2-oxo-2,3-dihydroquinazolin-4(*1H*)-ylidene)-amino)ethyl) imidazolidine-2,4-diones **5**, 2-(phenylamino)phenyl)-5,6-dihydroimidazo[1,5-*a*]pyrazine-diones **6**, and 5-oxo-tetrahydropyrazinoquinoline-4-carboxamides **7**. The molecular structures of the isolated compounds were suggested according to ^1^H, ^13^C and ^15^N NMR and electrospray-ionization mass spectrometry experiments. The structures of compounds **5d**, **7a**, and **7b** were proved using X-ray analysis of crystalline material. Moreover, we proposed a mechanism for the molecular rearrangement of starting compounds **2** providing two hitherto unknown hydantoin-based derivatives **5** and **6**. Retro-Claisen condensation seems to be a key step in the formation of the corresponding compounds. The presented work extends the set of compounds containing a hydantoin structural motif and offers a new approach for their synthesis. According to the previously described anticancer activity of several hydantoin-based derivatives [28], we decided to screen compounds **5**, **6**, and **7** for antiproliferative activity by using two cancer cell lines, K-562 and MV4;11. The inhibitory potency of these compounds for two types of protein kinases (CDK2/cyclin E and ABL1) was also assayed. However, no biological activity was observed for the tested molecules.

## Figures and Tables

**Table 1:** Results of the reactions of compounds **2**, **6** and **7**.

Starting Compound	Molar Ratio of 2 to KOCN	Product, (Yield, %)			
		5	6	7	8
**2a**	1:1.6			**7a** (47)	
**2a**	1:4.0			**7a** (57)	
**2b**	1:1.6	**5b** (18)		**7b** (63)	
**2c**	1:1.6			**7c** (32)	
**2c**	1:4.0			**7c** (68)	
**2d**	1:1.6	**5d** (17)	**6d** (13)	**7d** (68)	
**2d**	1:3.0	**5d** (17)	**6d** (8)	**7d** (31)	
**2e**	1:1.6	**5e** (22)	**6e** (14)	**7e** (49)	
**2e**	1:4.0	**5e** (50)	**6e** (6)	**7e** (29)	
**2f**	1:3.0		**6f** (20)		**8f** (18)
**6d**	1:3.0	**5d** (57)			
**7d**	1:3.0	**5d** (33)			

## Data Availability

Not applicable.

## Supplementary Materials

The following are available online at https://www.mdpi.com/article/10.3390/ijms23105481/s1.
