# Supplementary material for: Molecular Rearrangement of Pyrazino[2,3-c]quinolin-5(6H)-ones during Their Reaction with Isocyanic Acid
Source: Int J Mol Sci. 2022 May 13;23(10):5481. doi: 10.3390/ijms23105481 (PMC9143794; doi:10.3390/ijms23105481)
Supplement: Supplementary file 1 [file ijms-23-05481-s001.zip › ijms-1703532-supplementary.pdf]

Molecular Rearrangement of Pyrazino[2,3-*c*]quinolin-5(6*H*)-ones During Their Reaction with Isocyanic Acid

Antonín Klásek [a], Antonín Lyčka [b], Filip Křemen [a], Aleš Růžička [c] and Michal Rouchal [a,*]

[a)] Department of Chemistry, Faculty of Technology, Tomas Bata University in Zlín, Vavrečkova 5669, 760 01 Zlín, Czech Republic; (e-mail: rouchal@utb.cz)

[b)] University of Hradec Králové, Faculty of Science, Rokitanského 62, 530 03 Hradec Králové, Czech Republic

[c)] Department of General and Inorganic Chemistry, Faculty of Chemical Technology, University of Pardubice, Studentská 573, 532 10 Pardubice, Czech Republic

SUPPORTING INFORMATION

Table of Contents

^{1}H, ^{13}C and ^{15}N NMR spectra of compounds **2**, **3**, **4**, **5**, **6, 7, 8** S2

Crystal data and structure refinement of compounds **5d**, **7a, 7b** S35

ESI-MS spectra of compounds **2**, **3**, **4**, **5**, **6, 7, 8** S53

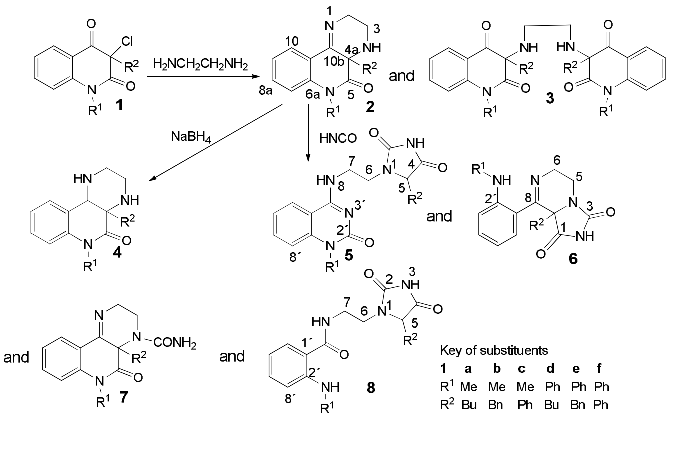

^{1}H, ^{13}C and ^{15}N NMR spectra of compounds 2, 3, 4, 5, 6, 7, 8

Table S1: ^{1}H, ^{13}C and ^{15}N chemical shifts of compounds **2** in DMSO-d$_6$.

Position	2a		2b		2c		2d		2e		2f	
	δ(H)	δ(C)	δ(H)	δ(C)	δ(H)	δ(C)	δ(H)	δ(C)	δ(H)	δ(C)	δ(H)	δ(C)
1	-	-	-	-52.7[a]	-	-	-	-	-	-	-	-53.0[a]
2	3.82	50.2	3.64	49.8	3.96	49.9	3.88	50.2	3.73	49.8	3.97	49.9
	3.49		3.02		3.81		3.57		3.10		3.94	
3	2.90	39.2	2.88	38.8	2.71	37.8	2.93	39.3	2.95	38.6	2.72	37.5
	2.44		2.45		2.65		2.54		2.56		2.60	
4	3.13	-	3.02	-351.7[a]	-	-	3.17	-	-	-	3.32	-339.3[a]
4a	-	63.2	-	64.1	-	65.6	-	63.5	-	64.4	-	66.1
5	-	171.7	-	170.8	-	169.2	-	171.8	-	170.9	-	169.4
6	-	-	-	-257.6[a]	-	-	-	-	-	-	-	-234.3[a]
6a	-	139.1	-	139.1	-	138.8	-	139.9	-	140.0	-	139.7
7	7.21	114.9	7.12	115.1	7.12	115.1	6.26	115.9	6.34	116.2	6.09	116.0
8	7.49	131.4	7.24	131.6	7.43	131.5	7.29	131.1	7.35	131.2	7.16	131.2
9	7.18	123.3	7.19	123.3	7.14	123.4	7.13	123.4	7.19	123.5	7.08	123.5
10	7.65	125.1	7.64	125.3	7.81	125.1	7.74	125.3	7.73	125.6	7.83	125.5
10a	-	123.9	-	123.8	-	124.2	-	123.3	-	123.4	-	123.7
10b	-	162.7	-	161.7	-	160.6	-	162.4	-	161.5	-	160.2
1′(R^1)	3.33	30.1	3.35	30.1	3.39	30.1	-	139.1	-	137.9	-	137.8
2′(R^1)	-	-	-	-	-	-	7.25[b]	b	b	b	7.20[b]	b
3′(R^1)	-	-	-	-	-	-	7.57	130.2	7.58	130.1	7.56	130.3
4′(R^1)	-	-	-	-	-	-	7.50	128.7	7.51	128.7	7.52	129.5
1′(R^2)	1.45	39.0	2.75	44.9	-	139.9	1.73	39.0	3.06	44.7	-	139.4
	1.31		2.68				1.59		2.91			
2′(R^2)	1.31	25.4	-	135.2	7.18	126.9	1.38	25.4	-	135.2	7.26	127.0
	1.05						1.15					
3′(R^2)	1.05	22.0	6.94	130.4	7.29	128.5	1.15	22.0	7.10	130.6	7.35	129.5
4′(R^2)	0.69	13.8	7.16	127.4	7.24	127.9	0.77	13.9	7.22	127.5	7.28	128.7
5′(R^2)	-	-	7.16	126.8	-	-	-	-	7.22	126.9	-	-

[a] δ(^{15}N) [b] broad overlapped signals

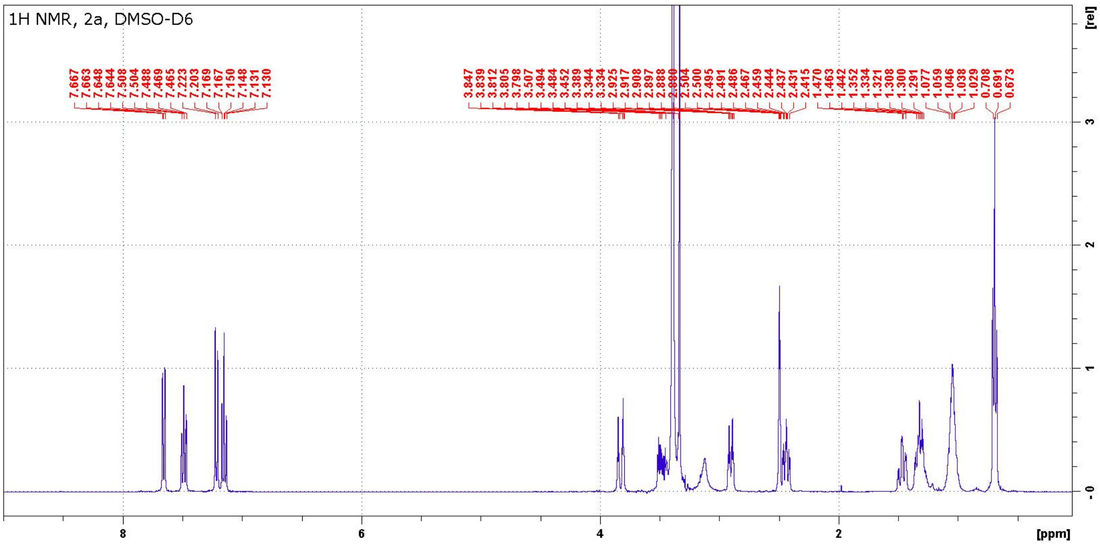

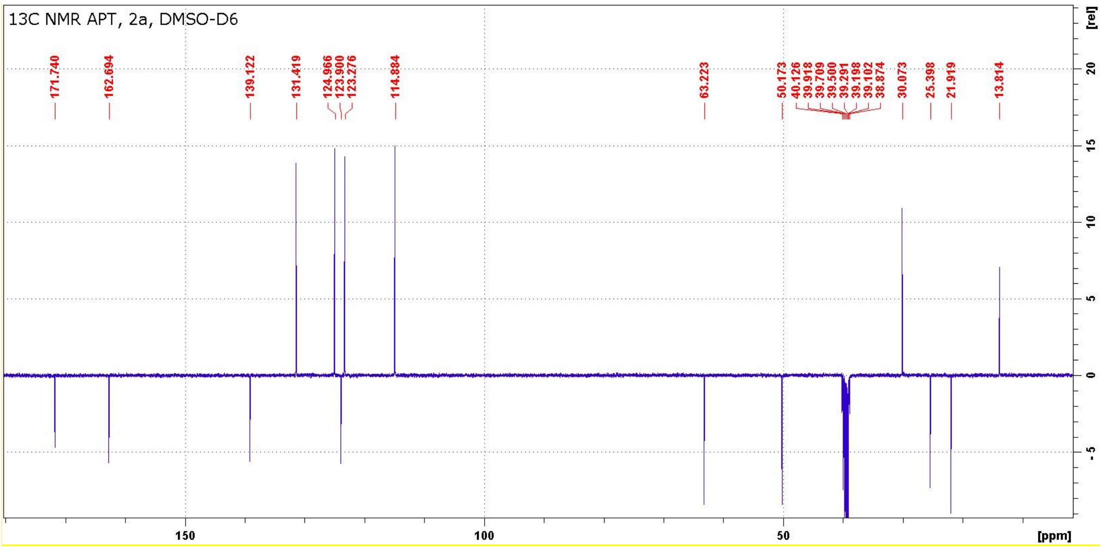

Figure S1: ^{1}H and ^{13}C NMR spectra of compound **2a**.

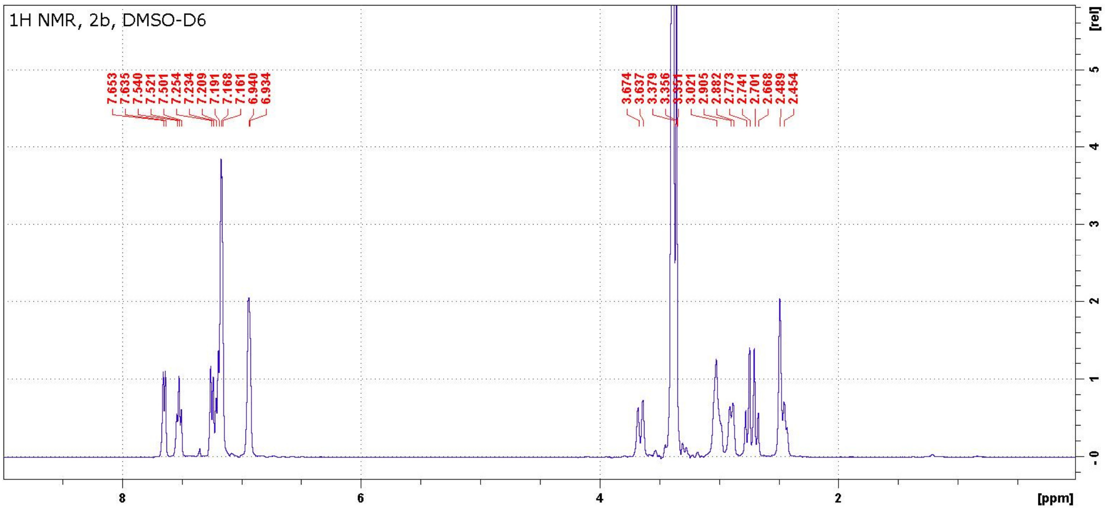

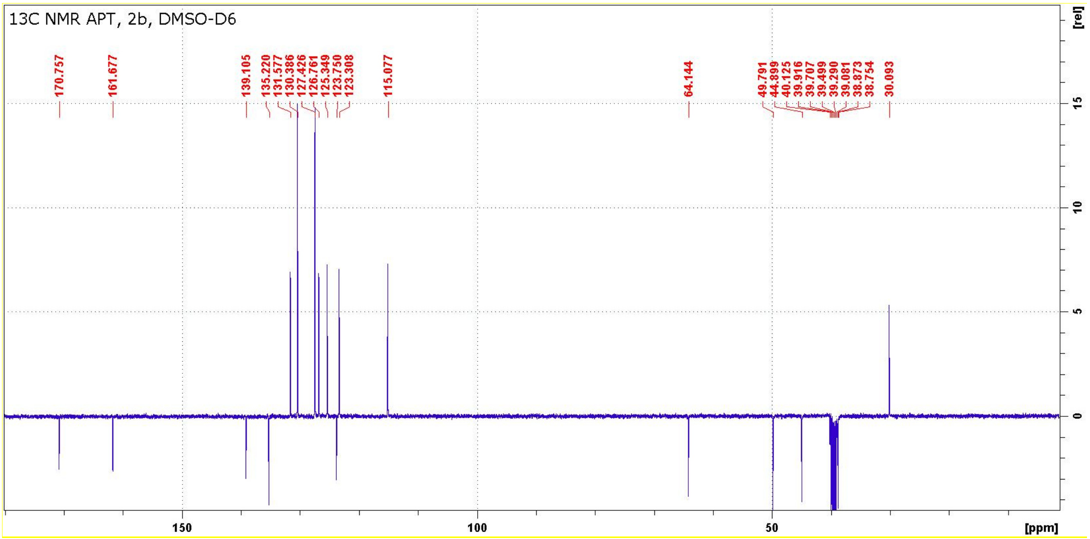

Figure S2: ^{1}H and ^{13}C NMR spectra of compound **2b**.

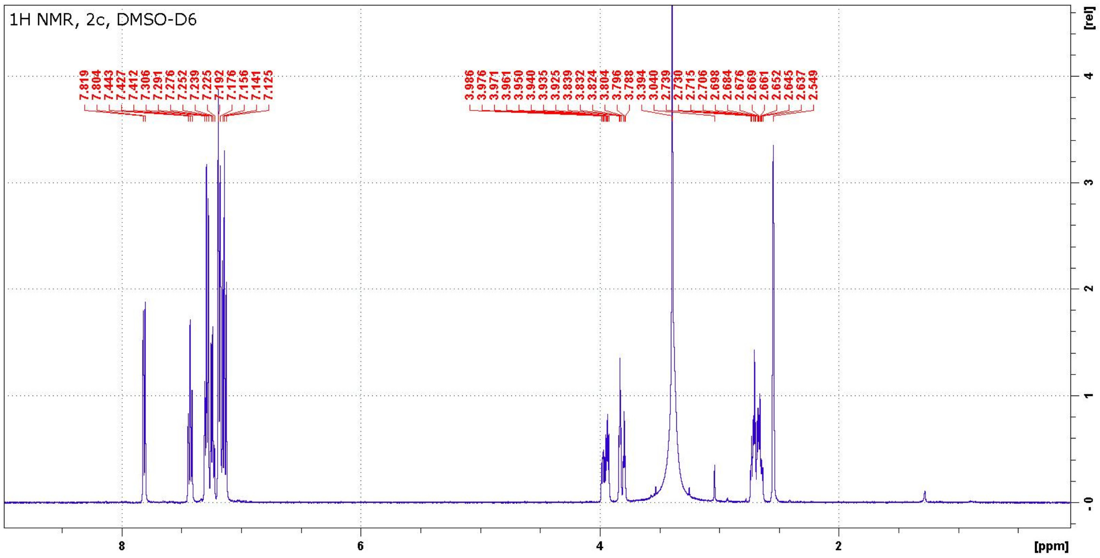

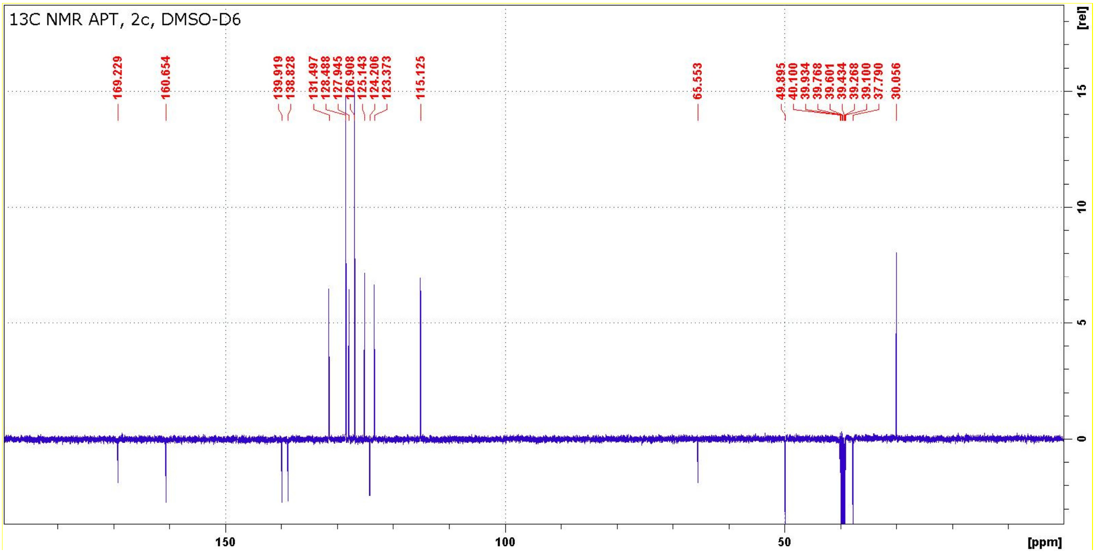

Figure S3: ^{1}H and ^{13}C NMR spectra of compound **2c**.

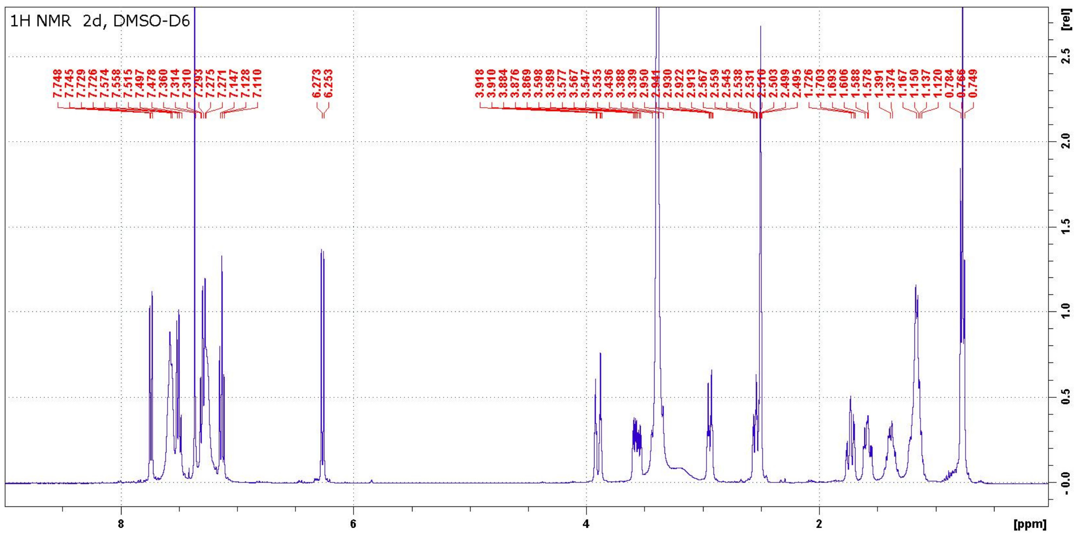

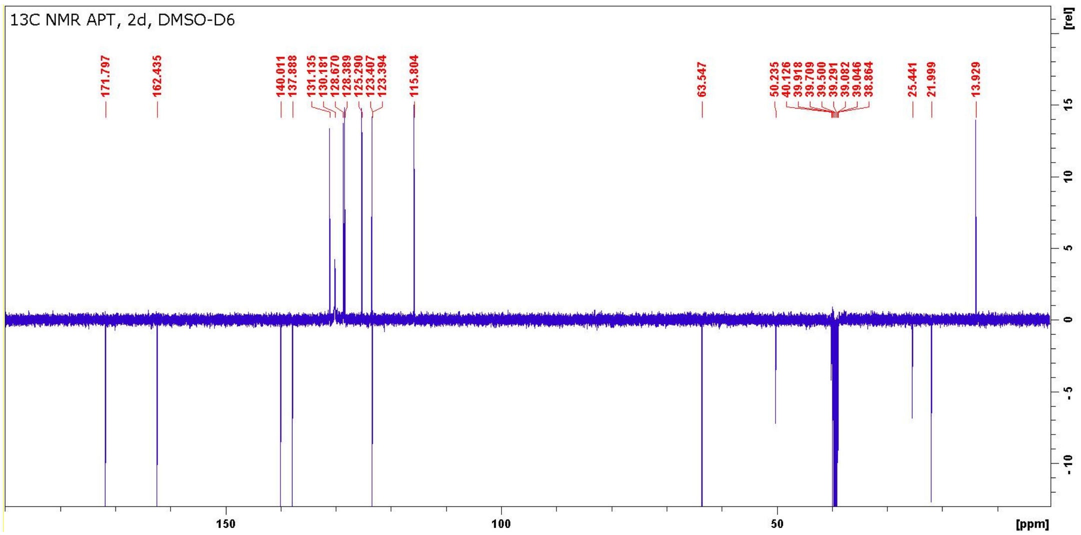

Figure S4: ^{1}H and ^{13}C NMR spectra of compound **2d**.

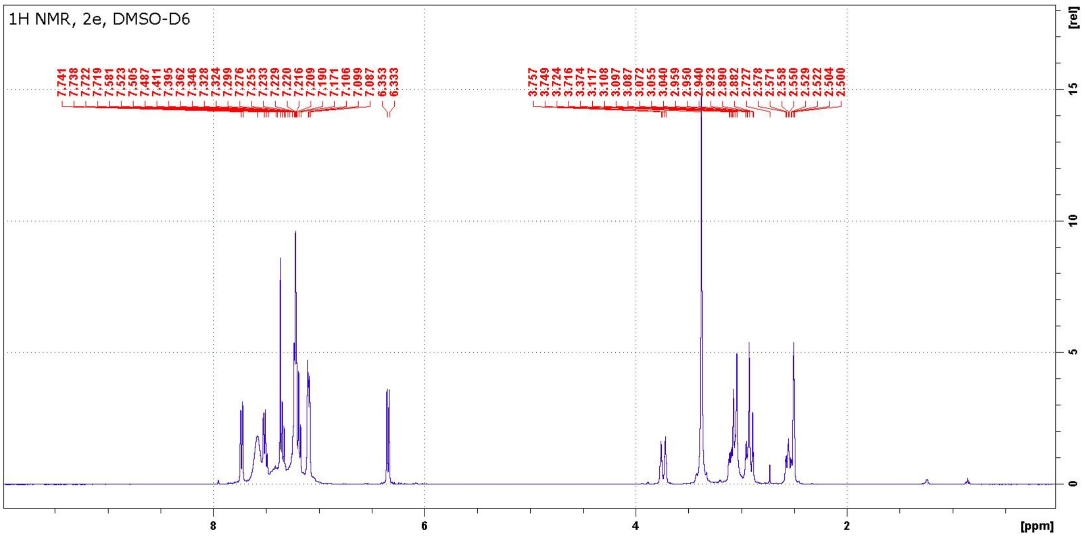

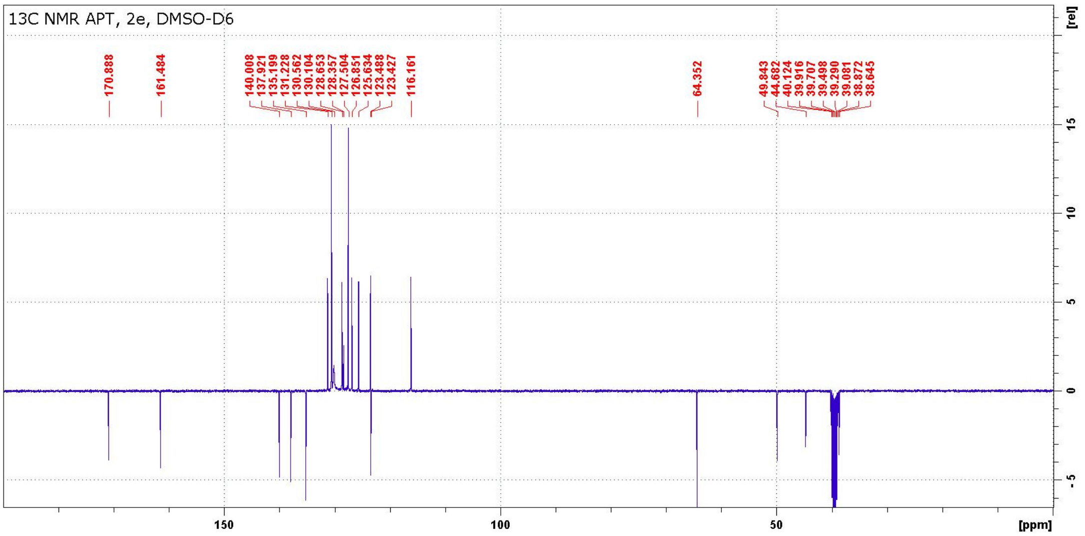

Figure S5: ^{1}H and ^{13}C NMR spectra of compound **2e**.

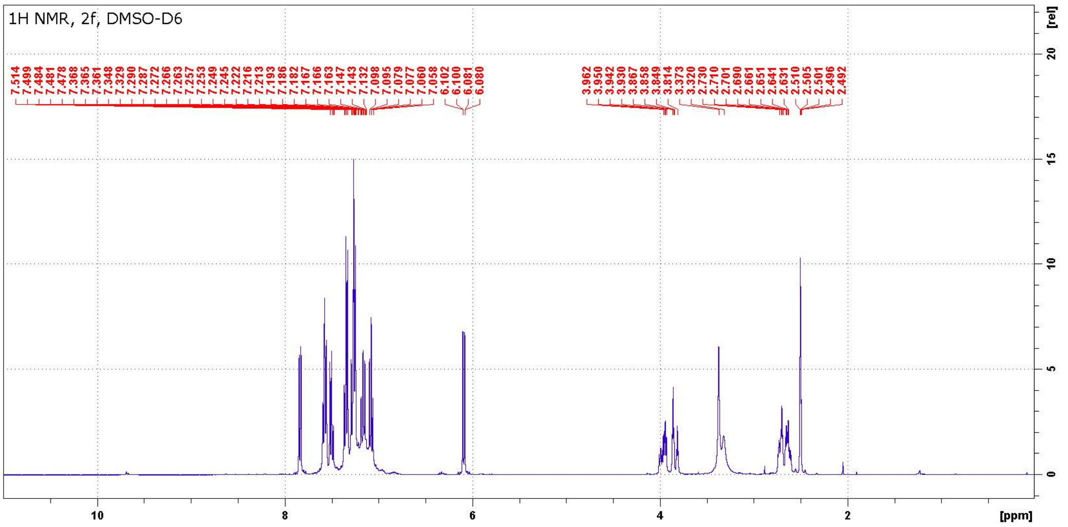

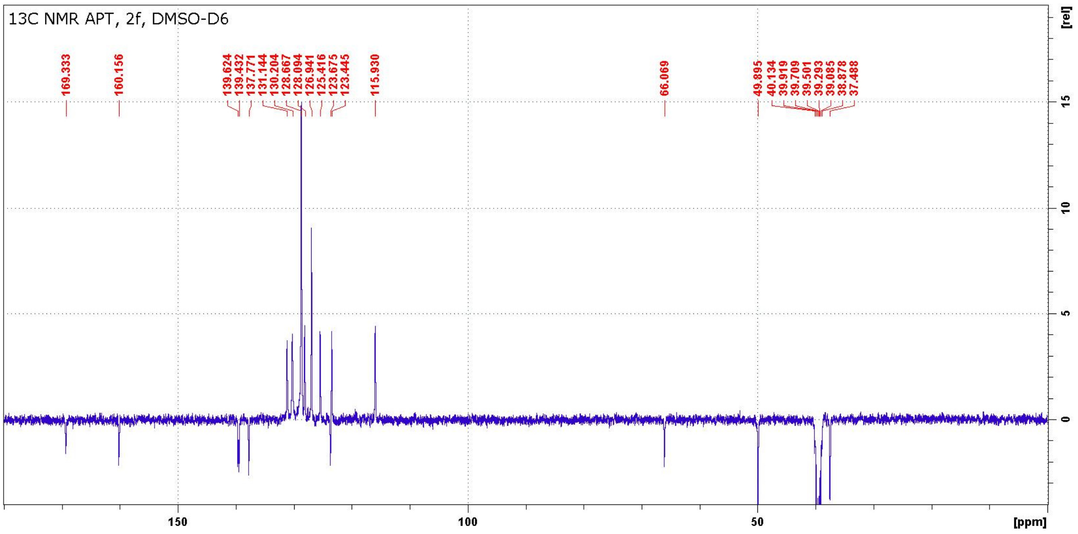

Figure S6: ^{1}H and ^{13}C NMR spectra of compound **2f**.

Table S2: ^{1}H and ^{13}C chemical shifts of diastereoisomers of compound **3c** and **3f** in DMSO-d_6.

Position	3c		3f	
	δ(H)	*δ*(C)	*δ*(H)	*δ*(C)
2	-	171.32, 171.23	-	171.33, 171.26
3	-	76.98, 76.96	-	77.23, 77.19
4	-	193.27, 192.72	-	193.10, 192.64
4a	-	120.71, 120.60	-	120.50, 120.44
5	7.75	127.48, 127.39	a	a
6	7.17	123.29, 123.27	a	123.48, 123.40
7	7.69	136.41, 136.39	7.46	135.93
	7.39	115.99, 115.96	6.32	116.59, 116.55
8a	-	142.3	-	142.0
NH	2.60	-	-	-
CH_2	2.59, 2.48	45.38, 45.17	2.58, 2.51	45.33, 45.17
1´(R^1)	3.53, 3.50	30.03, 30.00	-	139.63, 139.43
2´(R^1)	-	-	a	a
3´(R^1)	-	-	a	a
4´(R^1)	-	-	a	a
1´(R^2)	-	137.91, 137.86	-	137.78, 137.44
2´(R^2)	7.31	126.69, 126.64	a	a
3´(R^2)	7.28	128.84, 128.79	a	a
4´(R^2)	7.28	128.66, 128.62	a	a

[a] very low soluble compound, broadened overlapped signals, 7.12–7.82/126.9–131.2

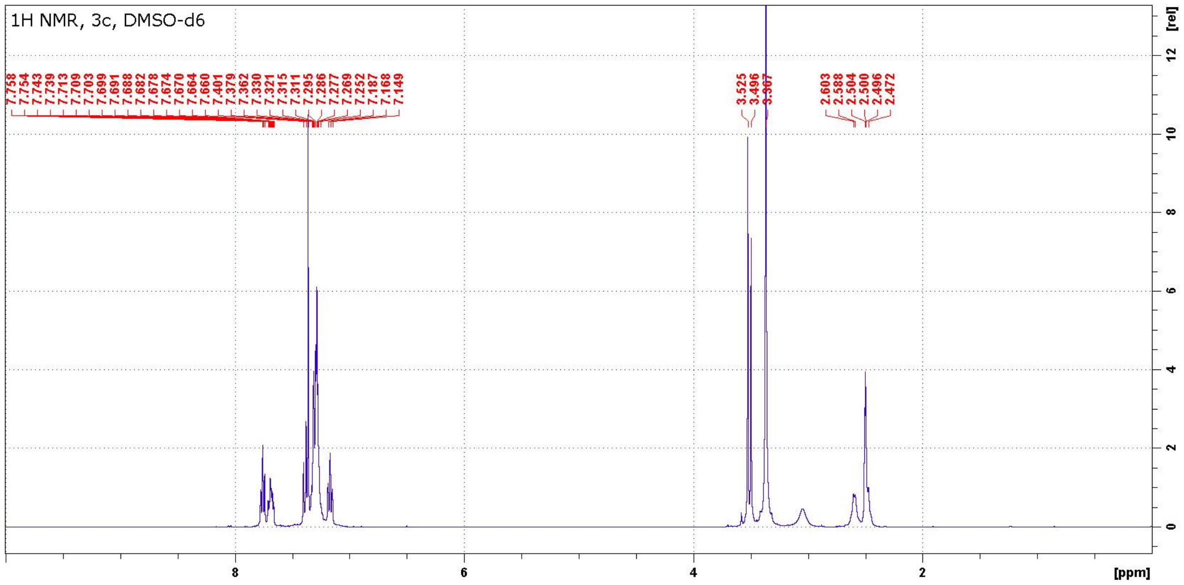

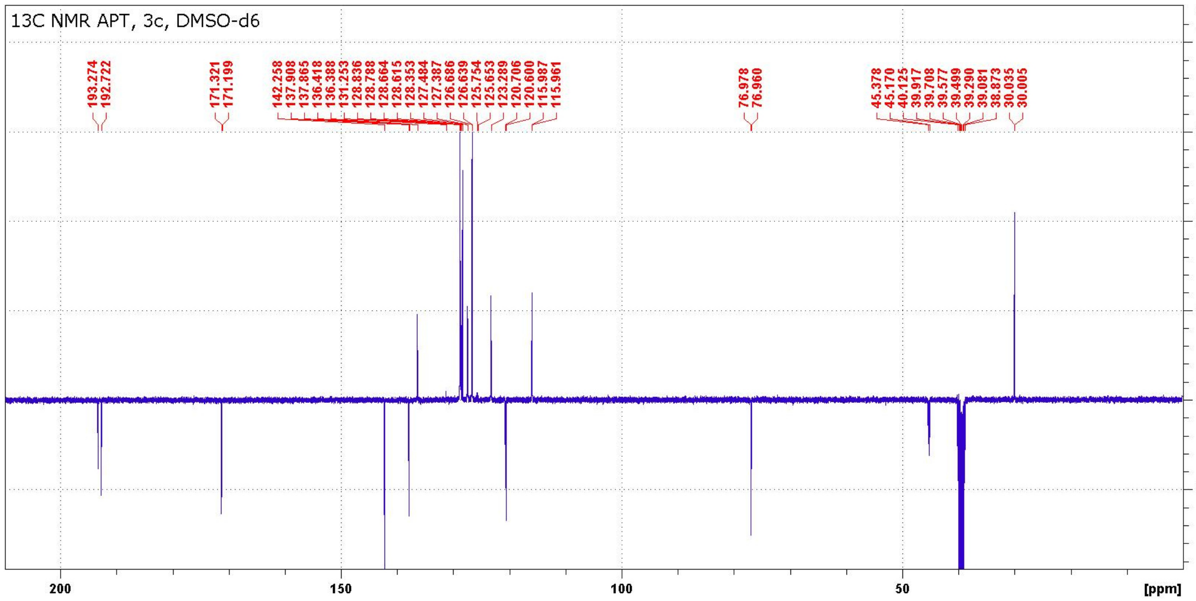

Figure S7: ^{1}H and ^{13}C NMR spectra of compound **3c**.

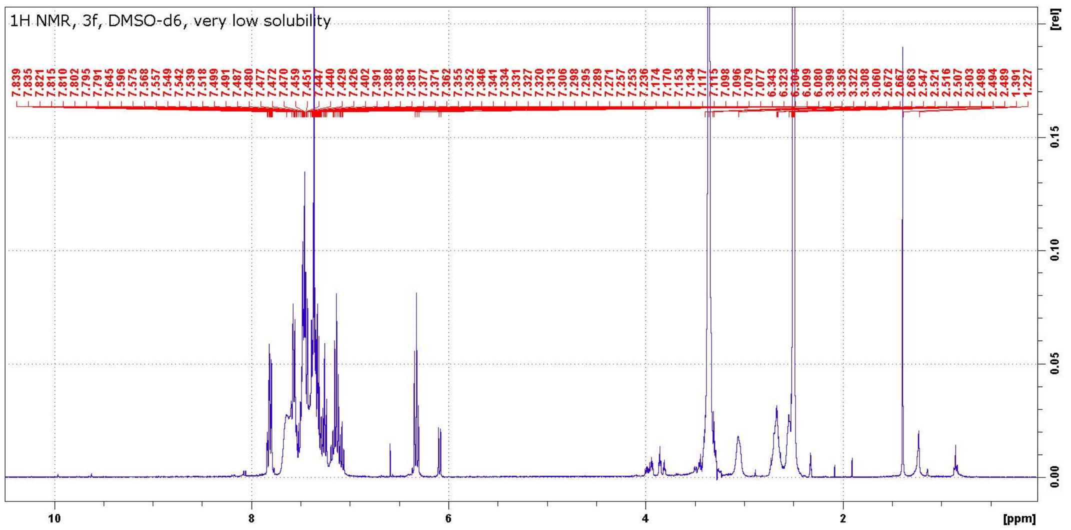

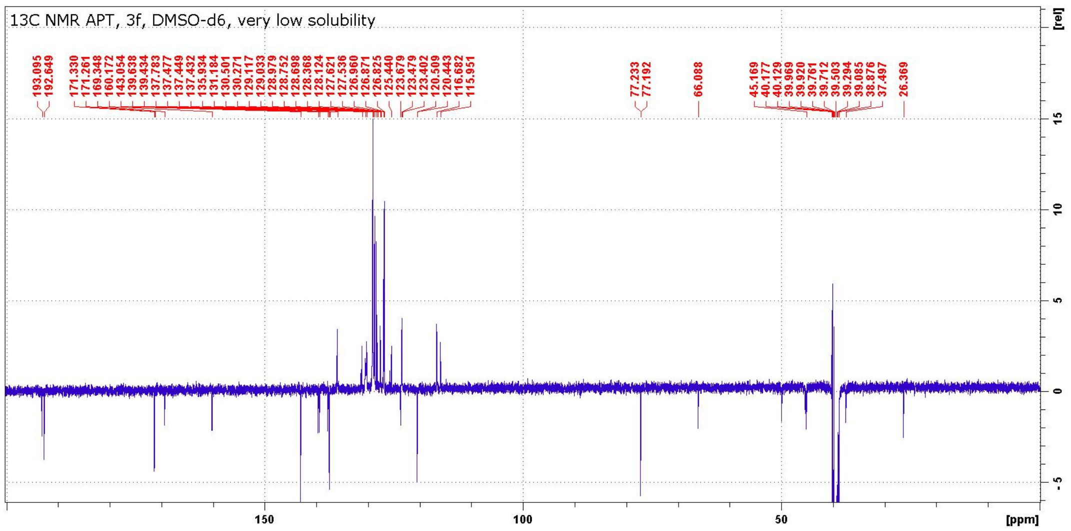

Figure S8: ^{1}H and ^{13}C NMR spectra of compound **3f**.

Table S3: ^{1}H, ^{13}C and ^{15}N chemical shifts of compounds **4** in DMSO-d_6.

Position	4a		4b		4c		4d		4e		4f	
	δ(H)	δ(C)	δ(H)	δ(C)	δ(H)	δ(C)	δ(H)	δ(C)	δ(H)	δ(C)	δ(H)	δ(C)
1	-	-	a	-	-	-	-	-344.6[b]	-		-	
2	2.90	45.4	3.11	45.5	2.99	46.1	2.96	45.4	3.08	45.5	3.09	45.5
	2.68		3.03		2.87		2.74		2.82		2.96	
3	2.65	39.9	2.77	40.1	2.60	40.4	2.73	39.7	3.15	40.4	2.70	40.1
	2.61		2.71		2.32		2.66		2.74		2.43	
4	-	-	a	-	-	-	-	-354.2[b]	-	-	-	-
4a	-	56.9	-	58.6	-	59.7	-	57.5	-	58.8	-	60.3
5	-	171.6	-	170.4	-	170.4	-	171.8	-	170.4	-	170.2
6	-	-	-	-	-	-	-	-235.0[b]	-	-	-	-
6a	-	138.1	-	138.2	-	138.0	-	138.6	-	138.5	-	138.2
7	7.06	114.1	7.13	114.3	6.95	114.7	6.15	115.5	6.29	116.1	6.01	115.8
8	7.27	127.6	7.37	127.8	7.20	127.4	7.07	127.4	7.14	127.5	6.98	127.1
9	7.27	123.4	7.32	123.5	7.13	123.1	7.04	123.9	7.14	123.8	7.11	123.3
10	7.06	122.7	7.13	122.8	7.49	122.8	7.35	123.0	7.44	123.1	7.53	123.2
10a	-	127.4	-	127.3	-	128.3	-	127.1	-	127.4	-	127.8
10b	3.87	58.2	3.98	58.4	4.25	58.0	4.15	58.2	4.27	58.6	4.55	57.5
1´(R^1)	3.24	29.3	3.24	29.3	3.23	29.8	-	139.2	-	139.2	-	138.2
2´(R^1)	-	-	-	-	-	-	7.15	129.2	7.16	129.1	7.08	128.7
3´(R^1)	-	-	-	-	-	-	7.54	130.0	7.52	129.8	7.52	129.9
4´(R^1)	-	-	-	-	-	-	7.44	128.2	7.43	128.0	7.43	128.2
1´(R^2)	1.96	22.6	3.21	29.7	-	137.4	2.08	22.7	3.36	29.8	-	136.9
	0.57		2.04				0.89		2.27			
2´(R^2)	1.14	24.1	-	136.7	7.46	129.1	1.26	24.3	-	136.7	7.64	129.3
	0.86						1.02					
3´(R^2)	1.05	22.4	6.80	129.7	7.05	127.3	1.18	22.5	7.04	129.9	7.16	127.5
4´(R^2)	0.73	14.0	7.15	127.9	7.05	126.7	0.80	14.1	7.26	128.1	7.16	127.1
5´(R^2)	-	-	7.15	126.1	-	-	-	-	7.16	126.2	-	-

[a] δ(NH) = 2.92 and 2.12; [b] δ(^{15}N)

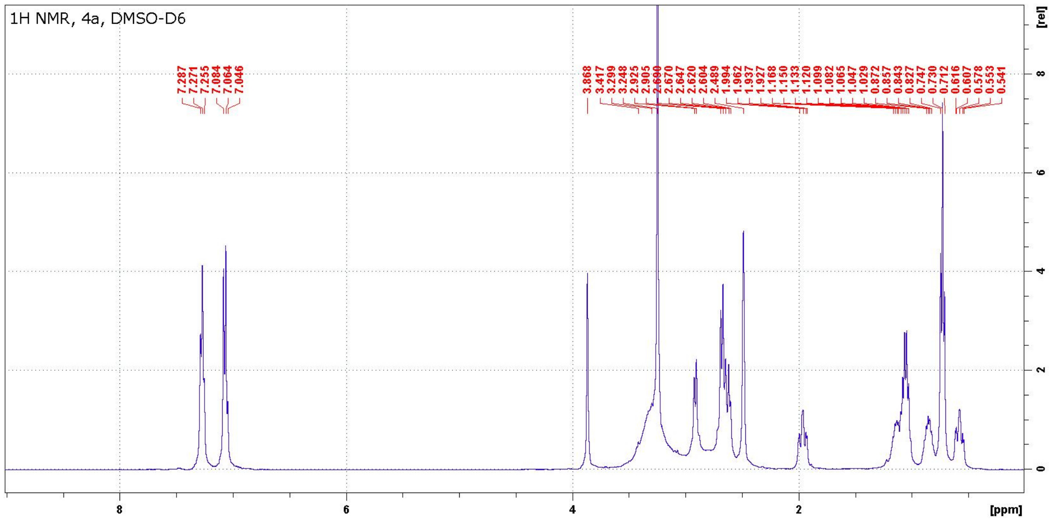

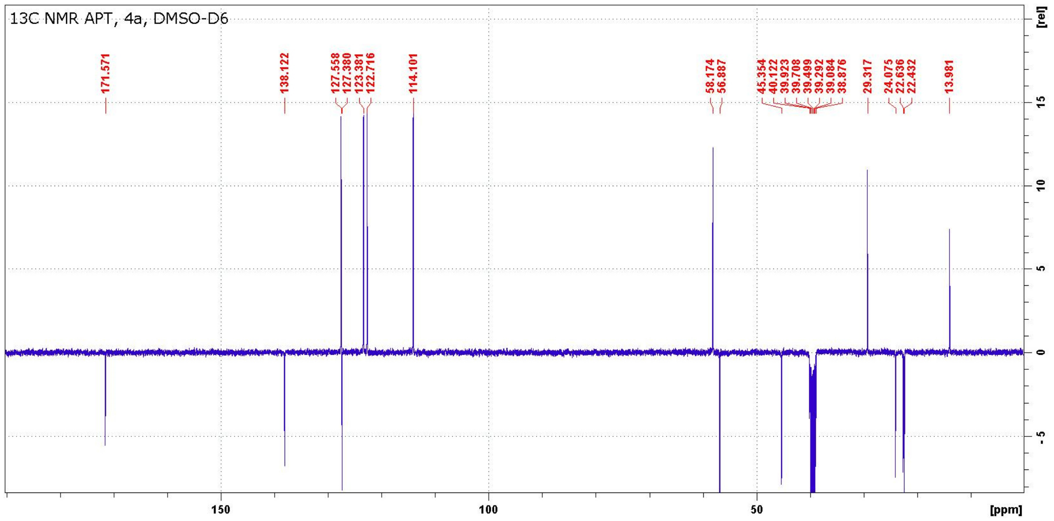

Figure S9: ^{1}H and ^{13}C NMR spectra of compound **4a**.

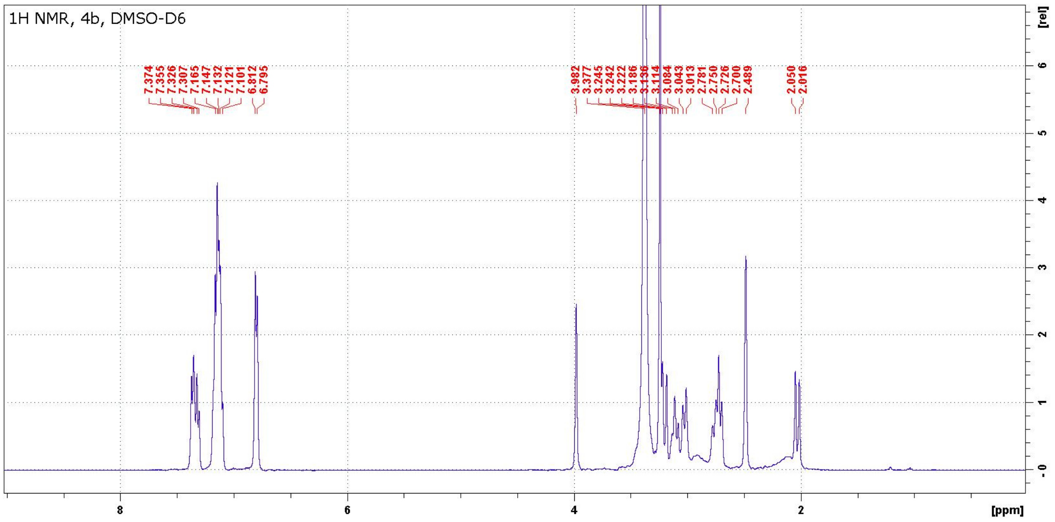

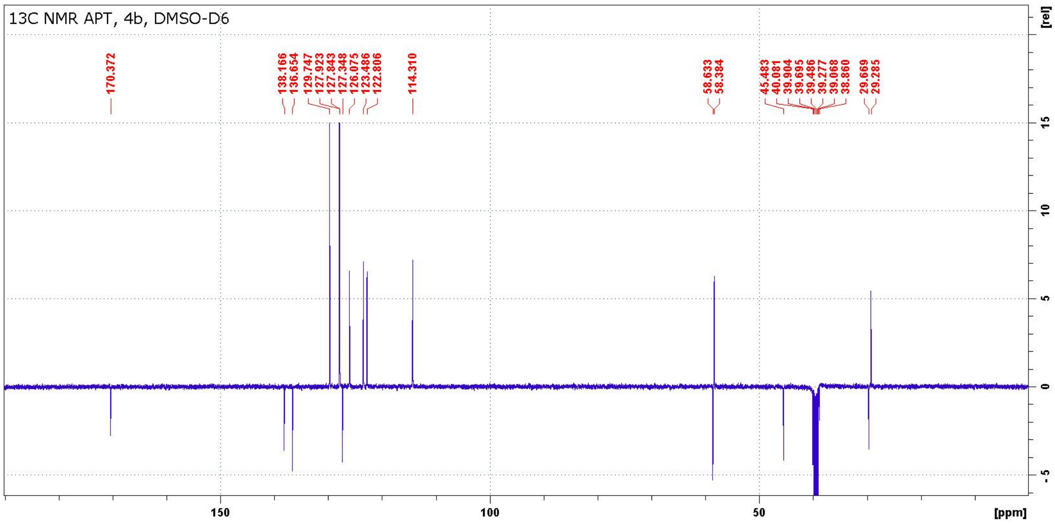

Figure S10: ^{1}H and ^{13}C NMR spectra of compound **4b**.

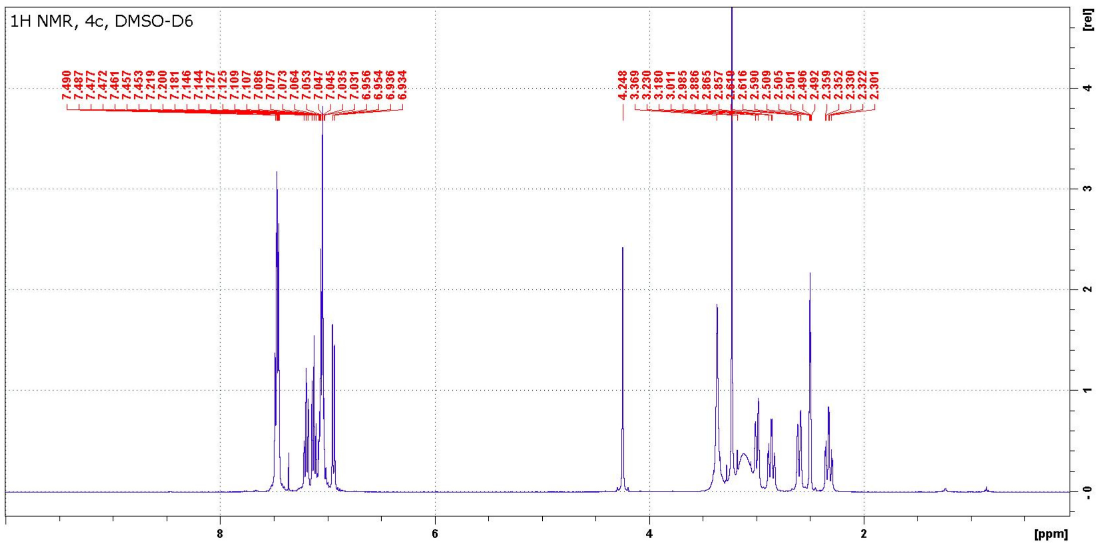

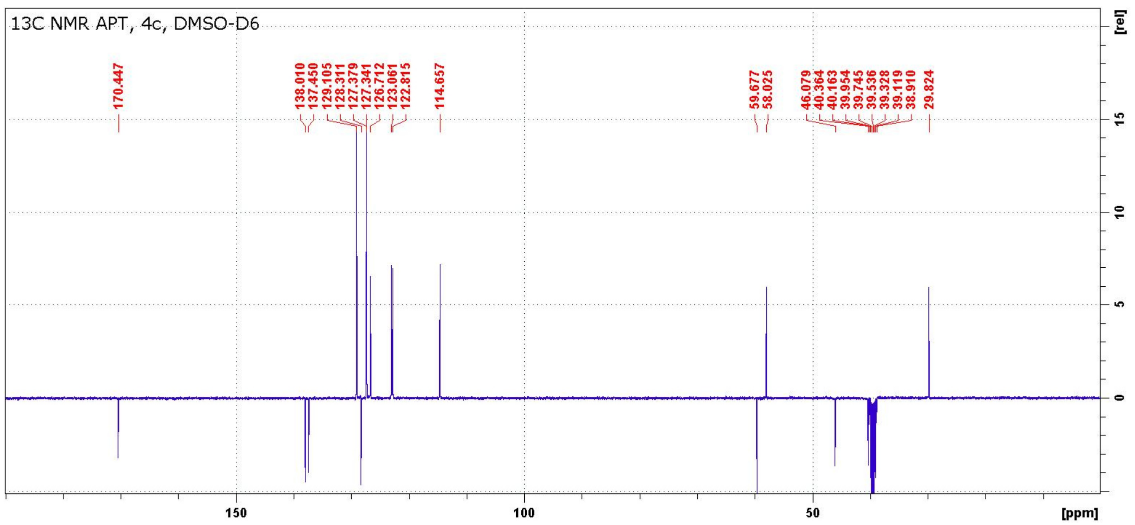

Figure S11: ^{1}H and ^{13}C NMR spectra of compound **4c**.

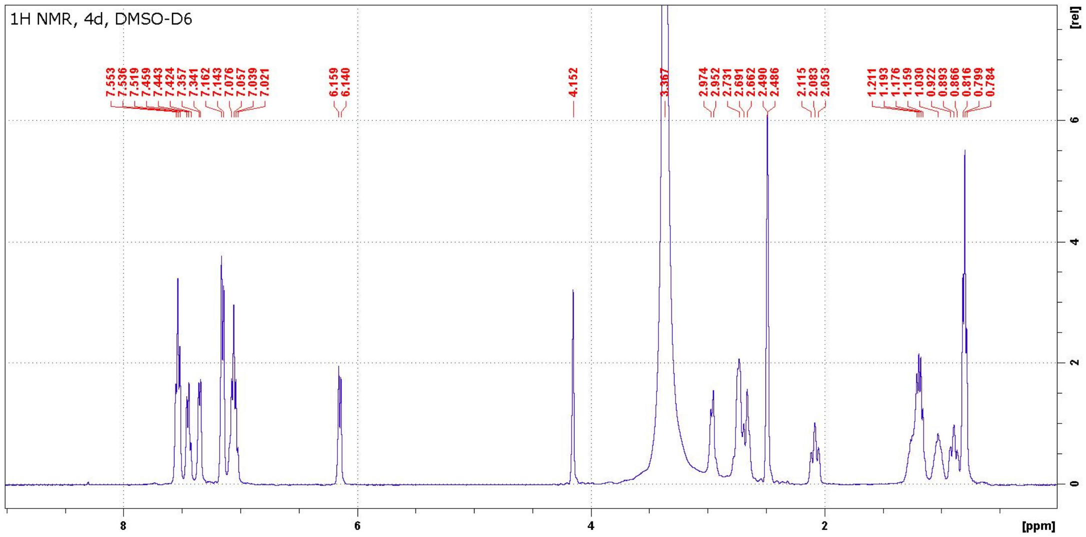

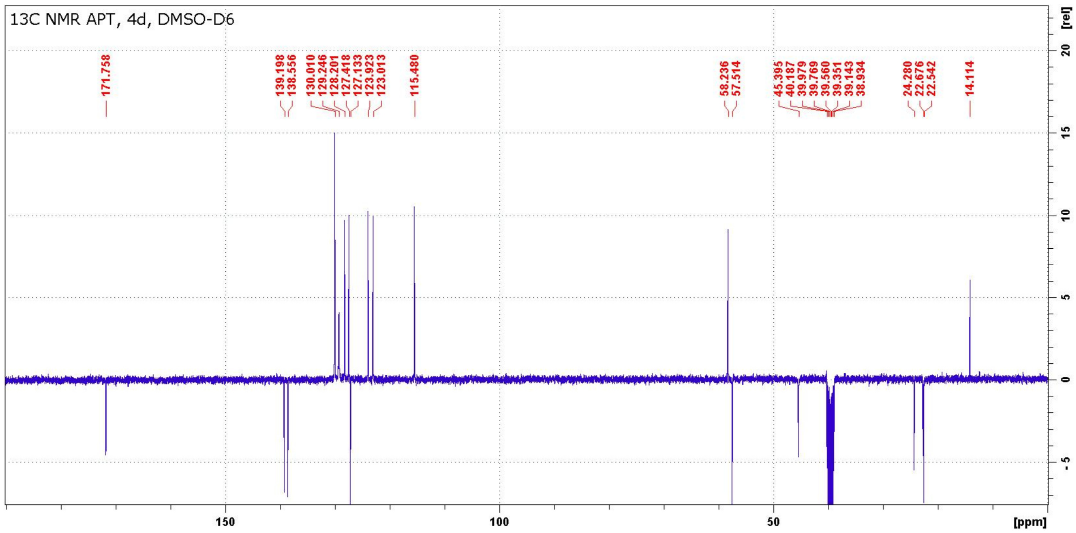

Figure S12: ^{1}H and ^{13}C NMR spectra of compound **4d**.

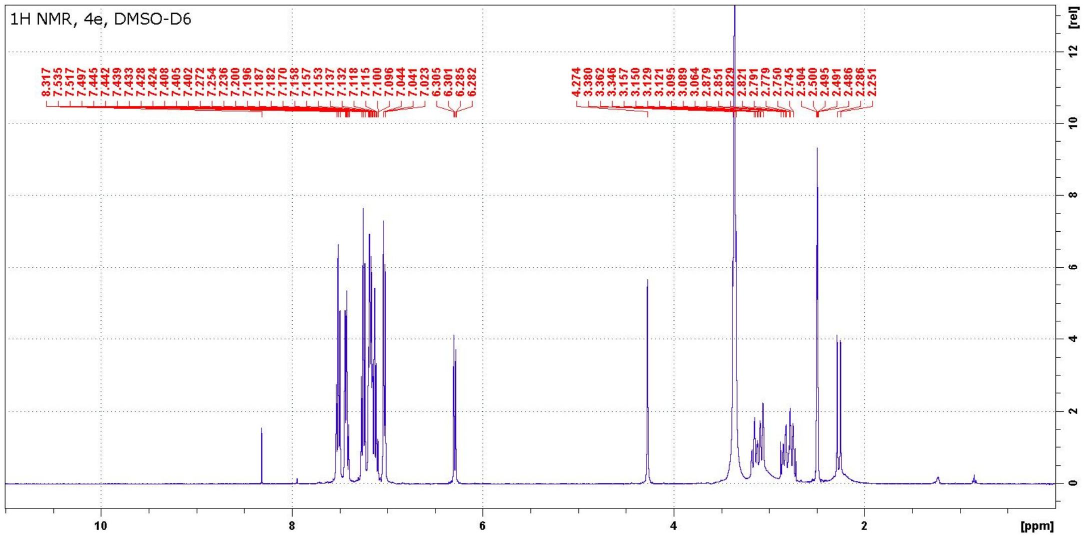

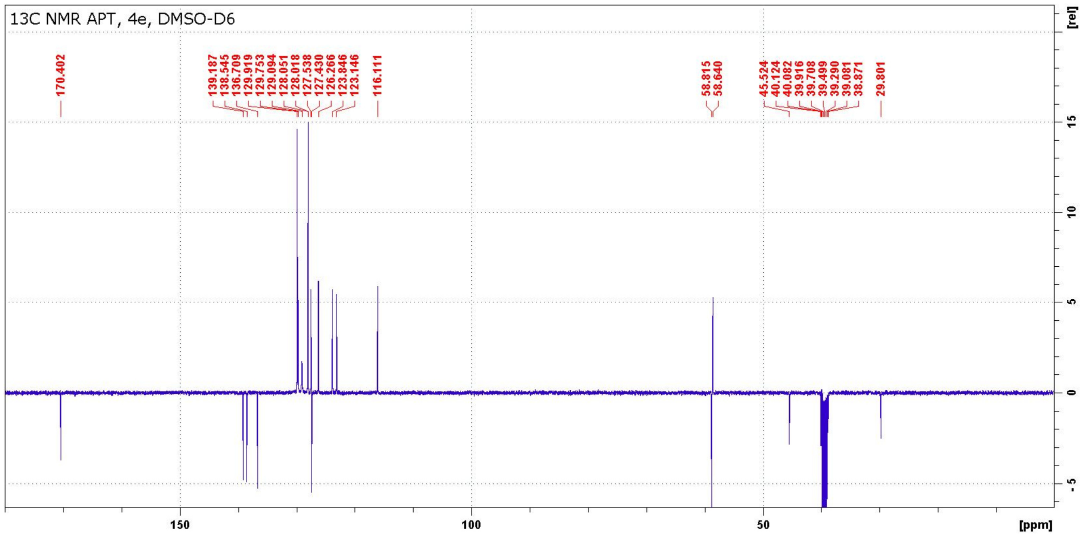

Figure S13: ^{1}H and ^{13}C NMR spectra of compound **4e**.

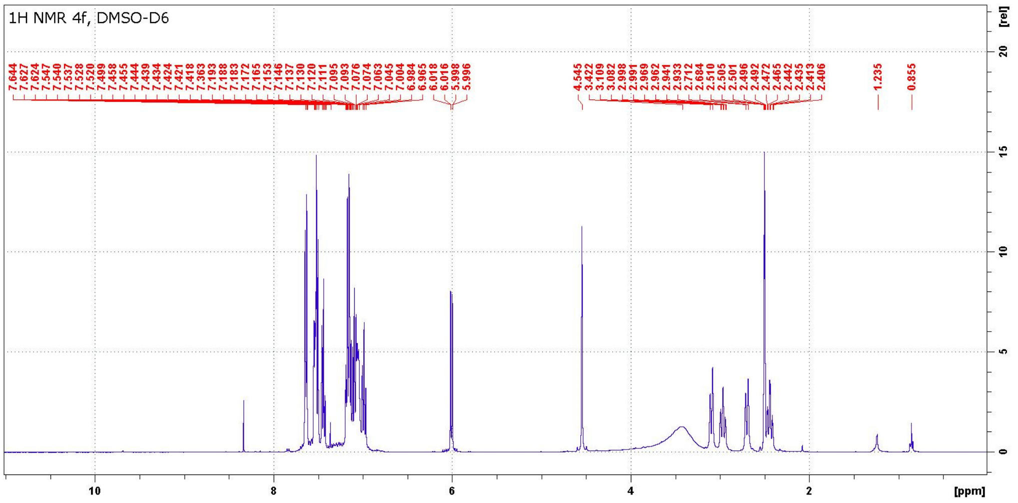

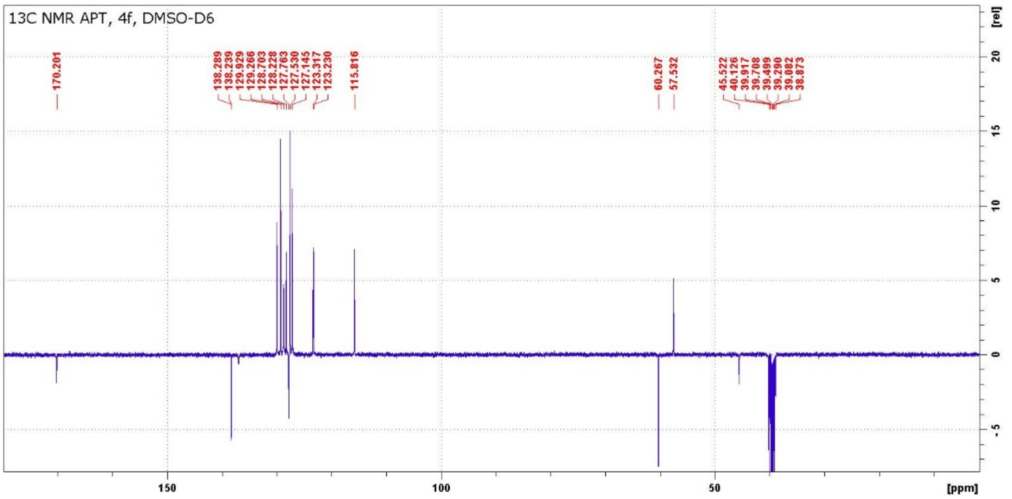

Figure S15: ^{1}H and ^{13}C NMR spectra of compound **4f**.

Table S4: ^{1}H, ^{13}C and ^{15}N chemical shifts and $^{1}J(^{15}N, ^{1}H)$ coupling constants of compounds **5** in DMSO-d$_6$.

Position	**5b**		**5d**		**5e**	
	δ(H)	δ(C)	δ(H)	δ(C)	δ(H)	δ(C)
1	-	-286.8[a]	-	-286.4[a]	-	-287.6[a]
2	-	156.5	-	156.92	-	156.6
3	10.53	n.o.	10.78	n.o.	10.58	232.2[a]
		95.2[b]		90.8[b]		94.6[b]
4	-	173.8	-	174.6	-	173.8
5	4.55	60.6	4.26	59.9	4.60	60.6
6	3.61	38.7	3.67	38.8	3.69	38.8
7	3.85	38.1	3.82	38.6	3.82	38.2
	3.17		3.24		3.20	
8	8.37	-286.8[a]	8.58	-285.0[a]	8.56	-285.0[a]
		88.5[b]		92.4[b]		92.7[b]
1′	-	-262.9[a]	-	-241.2[a]	-	-241.1[a]
2′	-	155.2	-	154.6	-	154.5
3′	-	-171.2[a]	-	-171.4[a]	-	-171.7[a]
4′	-	160.0	-	160.6	-	160.6
4a′	-	109.8	-	109.4	-	109.4
5′	7.95	123.7	8.04	123.7	8.02	123.6
6′	7.20	121.0	7.19	121.4	7.21	121.3
7′	7.67	133.9	7.45	133.6	7.49	133.5
8′	7.34	114.4	6.40	115.0	6.41	115.0
8a′	-	142.8	-	143.7	-	143.7
1′(R^1)	3.43	30.0	-	138.2	-	138.2
2′(R^1)	-	-	7.26	128.3	7.24	128.2
3′(R^1)	-	-	7.58	129.9	7.59	129.9
4′(R^1)	-	-	7.49	128.3	7.50	128.2
1′(R^2)	3.14	33.4	1.76	27.4	3.20	30.9
	3.01		1.73		3.05	
2′(R^2)	-	135.4	1.20	25.0	-	135.4
			1.04			
3′(R^2)	7.10	128.2	1.20	21.9	7.14	128.2
4′(R^2)	7.20	129.4	0.79	13.8	7.28	129.4
5(R^2)	7.20	126.7	-	-	7.22	126.7

[a] $\delta(^{15}N)$; [b] $^{1}J(^{15}N, ^{1}H)$

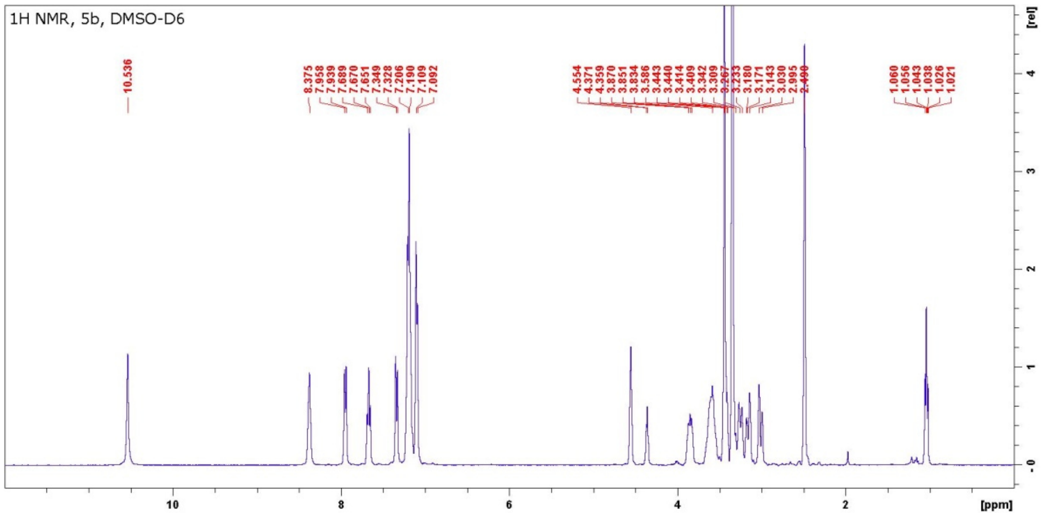

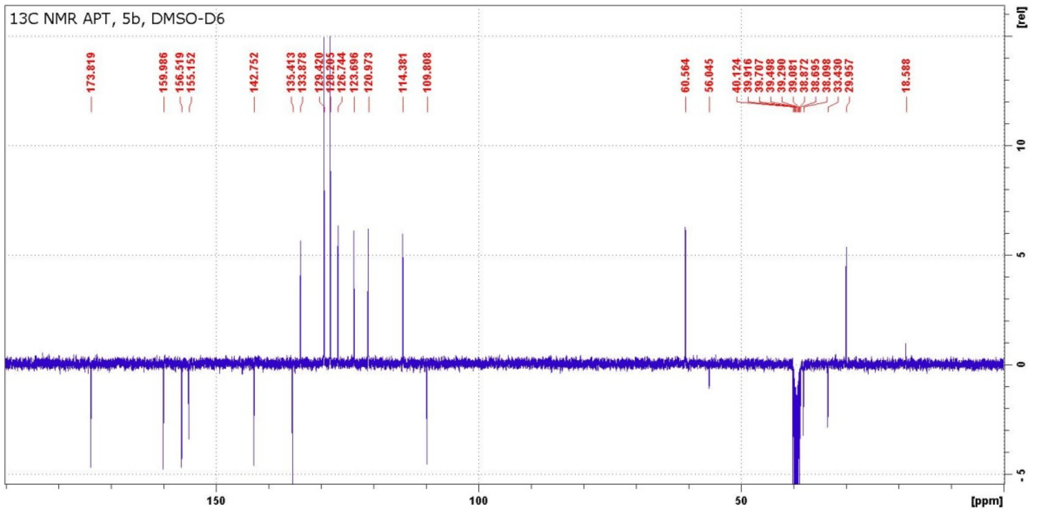

Figure S16: ^{1}H and ^{13}C NMR spectra of compound **5b**.

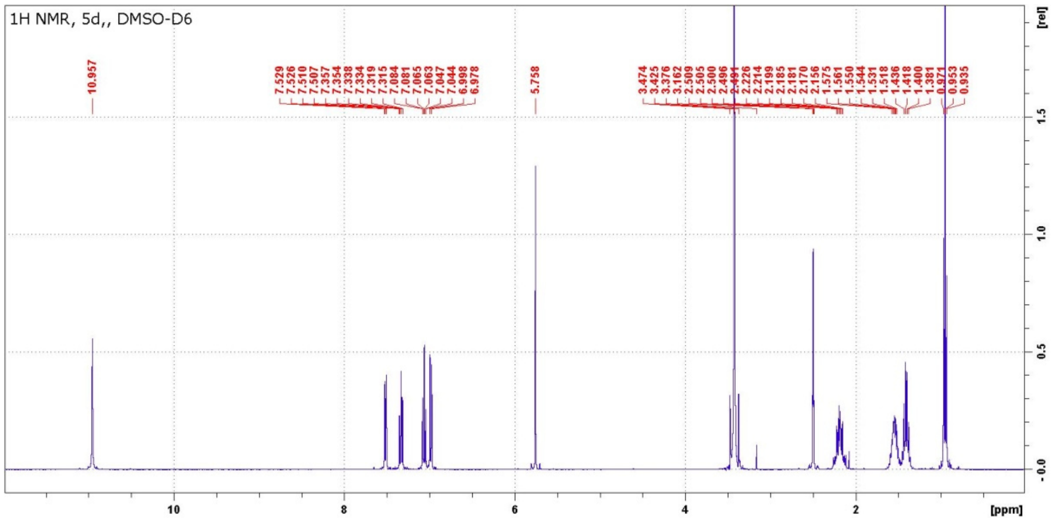

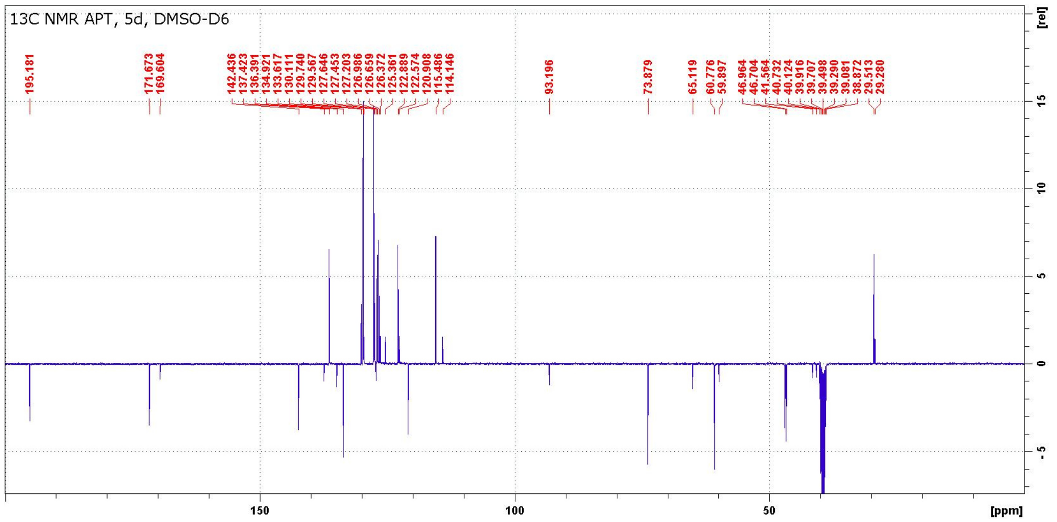

Figure S17: ^{1}H and ^{13}C NMR spectra of compound **5d**.

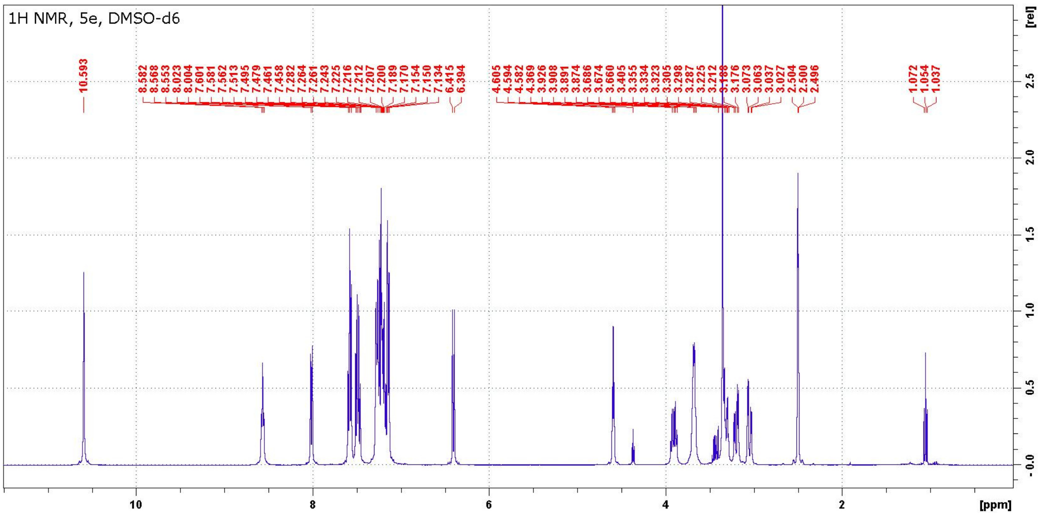

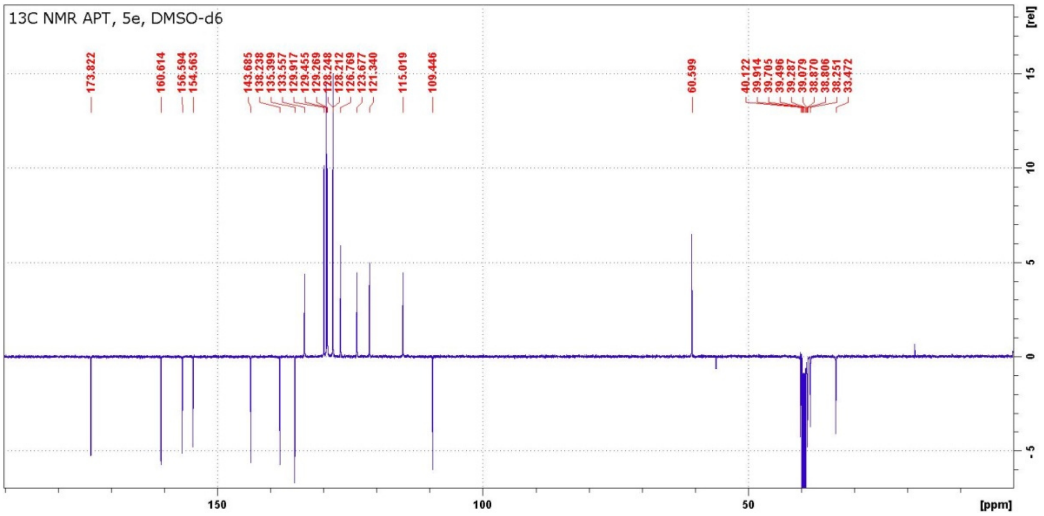

Figure S18: ^{1}H and ^{13}C NMR spectra of compound **5e**.

Table S5: ^{1}H, ^{13}C and ^{15}N chemical shifts of compounds **6** in DMSO-d$_6$

Position	**6d**		**6e**		**6f**	
	δ(H)	δ(C)	δ(H)	δ(C)	δ(H)	δ(C)
1	-	170.6	-	169.9	-	170.3
2	11.01	n.o.	10.68	n.o.	11.05	n.o.
3	-	156.2	-	155.8	-	155.4
4	-	-290.8[a]	-	-291.4[a]	-	-285.5[a]
5	3.82	46.4	3.93	46.6	4.03	45.5
			3.86		3.55	
6	3.82	33.9	3.78	34.0	3.48	36.2
	3.15		3.42		3.34	
7	-	-53.7[a]	-	-50.6[a]	-	-58.2[a]
8	-	163.1	-	162.9	-	165.0
8a	-	68.0	-	68.3	-	68.9
1′	-	128.3	-	128.2	-	125.3
2′	-	141.7	-	142.1	-	138.8
3′	7.10	117.9	7.12	117.7	7.19	118.9
4′	7.19	128.7	7.22	128.9	7.23	130.8
5′	6.86	119.7	6.93	120.1	6.82	119.4
6′	7.10	129.3	7.22	129.4	7.25	131.6
2′	7.15	-295.9[a]	7.37	-295.6[a]	8.85	-292.2[a]
		90.3[b]		90.4[b]		87.2[b]
1′(R^1)	-	143.6	-	143.5	-	142.8
2′(R^1)	7.02	118.6	7.02	119.1	7.01	118.9
3′(R^1)	7.19	128.7	7.21	128.7	7.22	128.9
4′(R^1)	6.83	120.3	6.83	120.5	6.89	120.9
1′(R^2)	1.94	32.7	3.26	38.5	-	137.3
			3.36			
2′(R^2)	1.15	24.7	-	133.8	7.39	126.5
	0.98					
3′(R^2)	1.15	21.7	7.02	131.2	7.42	128.9
4′(R^2)	0.74	13.7	7.20	128.3	7.42	130.0
5′(R^2)	-	-	7.22	127.1	-	-

[a] $\delta(^{15}N)$; [b] $^1J(^{15}N, ^1H)$; n.o. = not observed

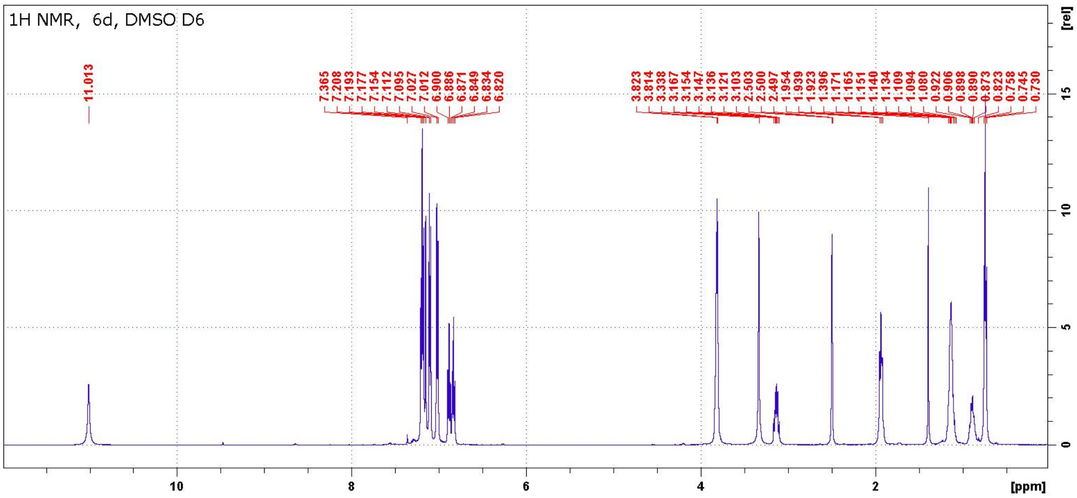

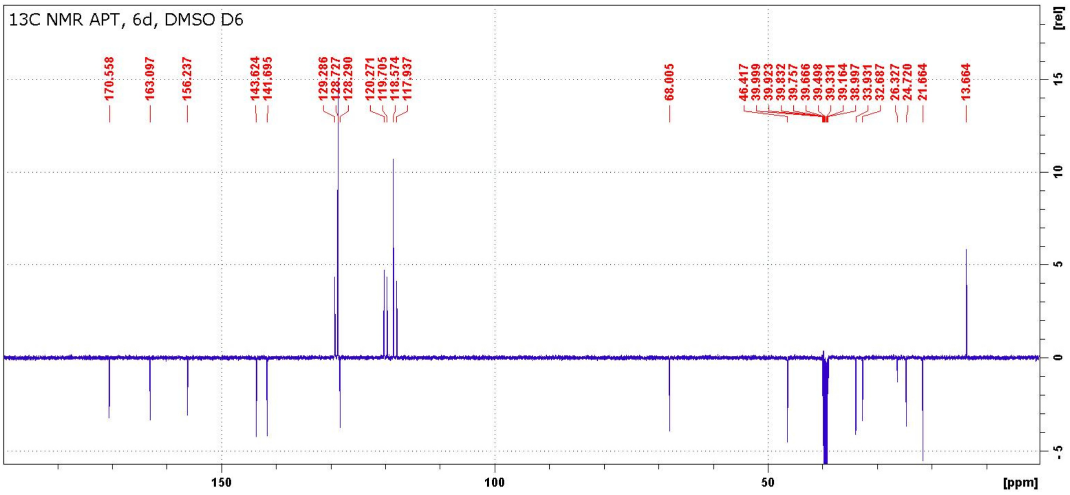

Figure S19: ^{1}H and ^{13}C NMR spectra of compound **6d**.

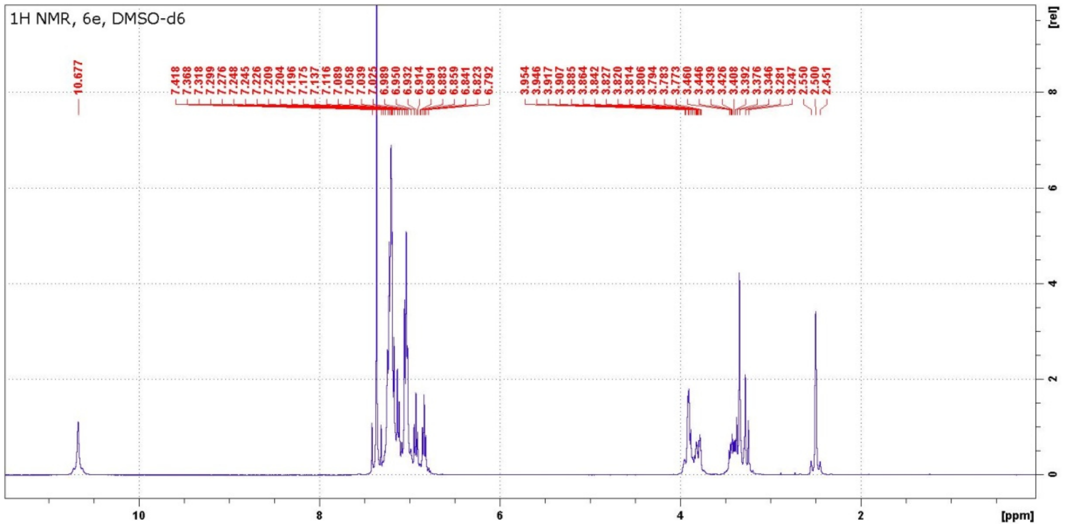

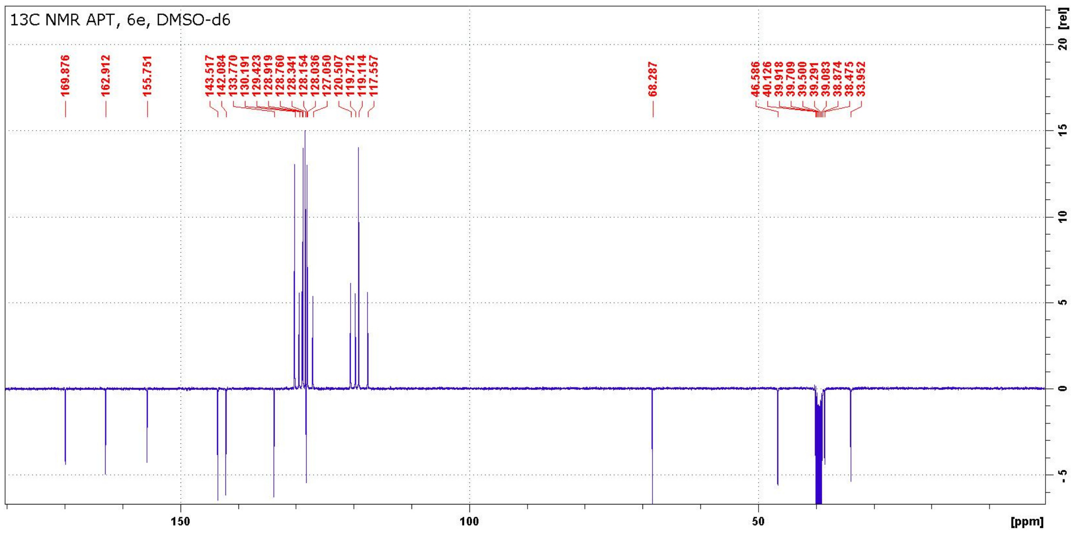

Figure S20: ^{1}H and ^{13}C NMR spectra of compound **6e**.

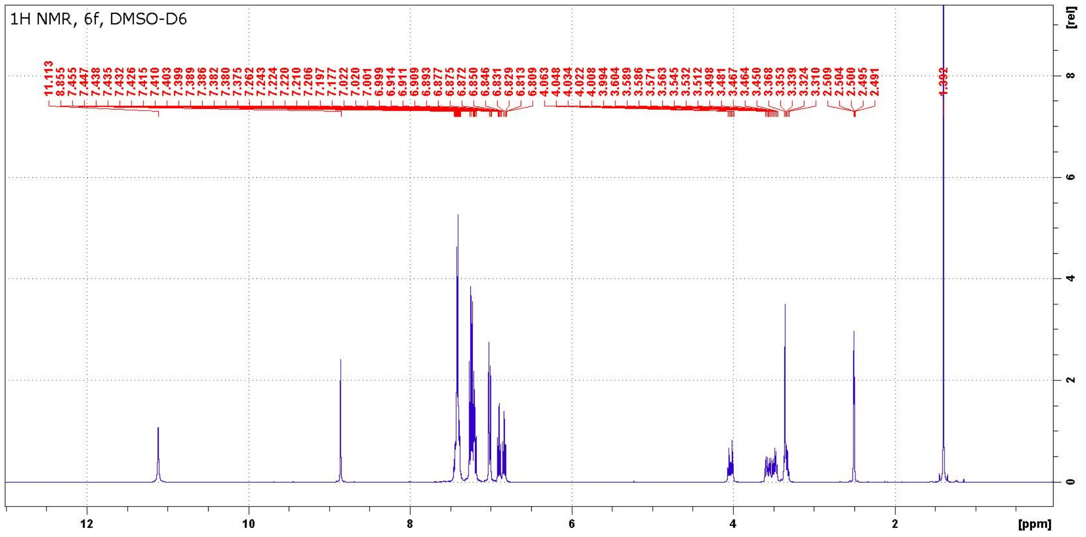

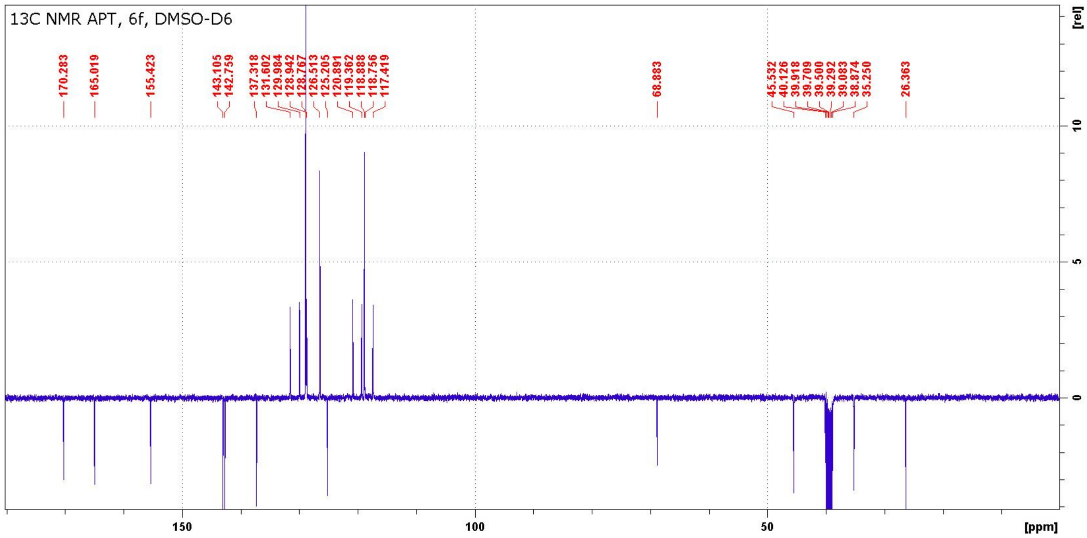

Figure S21: ^{1}H and ^{13}C NMR spectra of compound **6f**.

Table S6: ^{1}H, ^{13}C and ^{15}N chemical shifts and $^{1}J(^{15}N, ^{1}H)$ coupling constants of compounds **7** in DMSO-d_6 or in $CDCl_3$.

Position	7a		7b		7c[a]		7d[a]		7e	
	δ(H)	δ(C)	δ(H)	δ(C)	δ(H)	δ(C)	δ(H)	δ(C)	δ(H)	δ(C)
1	-		-	-	-	-62.7[b]	-	-54.1[b]	-	-
2	3.94	n.o.	3.66	48.6	4.70	50.5	4.21	50.4	3.66	48.5
					2.83		3.69	-		
3	3.80	40.1	3.61	40.4	4.27	39.7	3.48	40.1	3.74	40.4
	3.49		2.74		4.00		2.77	-	2.74	
4	-		-	-	-	-288.5[b]	-	-296.1[b]	-	-
C=O	-	159.9	-	160.3	-	159.7	-	160.7	-	160.3
NH_2	6.12	-	6.22	-	4.53	-300.9[b, c]	5.21	-301.9[b,d]	6.24	-
4a	-	n.o.	-	69.2	-	71.3	-	68.8	-	69.2
5	-	168.5	-	167.8	-	167.6	-	171.5	-	167.7
6	-		-	-	-	-252.1[b]	-	-234.8[b]	-	-
6a	-	139.3	-	139.6	-	138.2	-	137.5	-	138.4
7	7.20	114.8	7.20	115.1	7.01	114.4	6.42	116.4	6.24	115.9
8	7.51	131.7	7.53	131.9	7.33	131.4	7.27	131.4	7.32	131.5
9	7.13	122.8	7.20	123.0	6.98	124.1	7.17	124.2	7.19	123.0
10	7.74	125.2	7.75	125.9	7.51	126.1	7.96	126.2	7.84	127.0
10a	-	124.6	-	122.9	-	124.6	-	123.1	-	122.5
10b	-	162.5	-	161.1	-	161.1	-	161.3	-	161.4
1′(R^1)	3.31	30.3	3.35	30.7	3.59	30.8	-	139.2	-	140.3
2′(R^1)	-	-	-	-	-	-	7.29	129.2	7.25	128.3
							7.19	128.2		
3′(R^1)	-	-	-	-	-	-	7.56	130.1	7.52	130.1
4′(R^1)	-	-	-	-	-	-	7.50	129.0	7.43	128.5
1′(R^2)	1.58	30.3	2.60,	39.7	-	137.7	3.19	34.8	3.05	39.8
			2.32				1.95	-	2.99	
2′(R^2)	1.19	25.2	-	135.4	7.20	126.3	1.36	25.2	-	135.3
	0.85						1.06	-		
3′(R^2)	1.03	21.7	6.91	129.8	7.20	129.4	1.21	22.1	6.99	129.5
4′(R^2)	0.66	13.7	7.20	127.5	7.20	129.1	0.81	13.7	7.20	127.6
5(R^2)	-	-	7.20	129.8	-	-	.	-	7.20	129.8

[a] in $CDCl_3$; [b] δ(^{15}N); [c] $^{1}J(^{15}N, ^{1}H)$ = 86.4 Hz; [d] $^{1}J(^{15}N, ^{1}H)$ = 83.6 Hz; n.o. = not observed

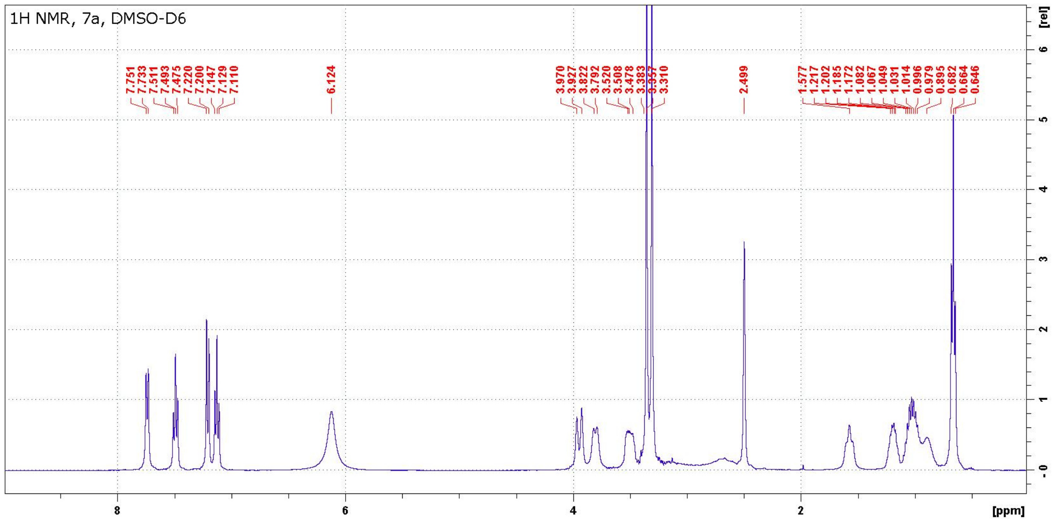

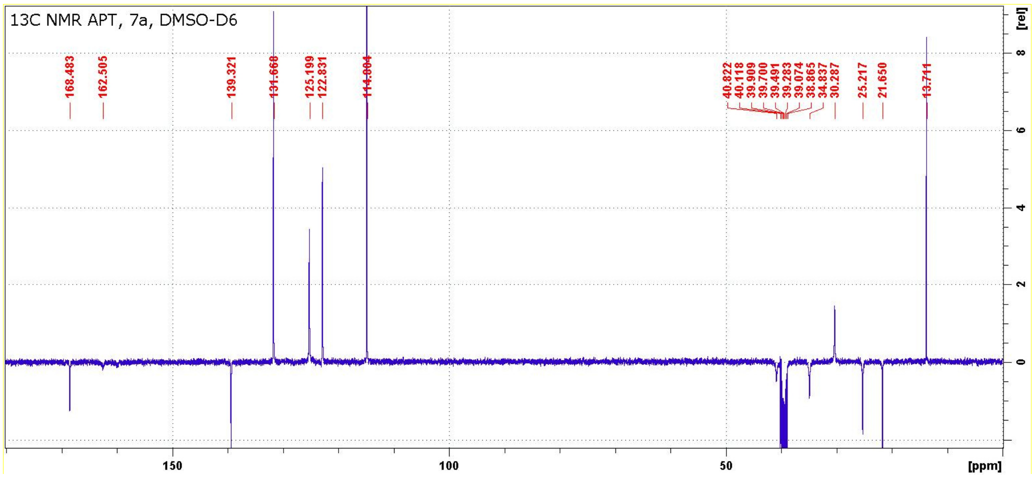

Figure S22: ^{1}H and ^{13}C NMR spectra of compound **7a**.

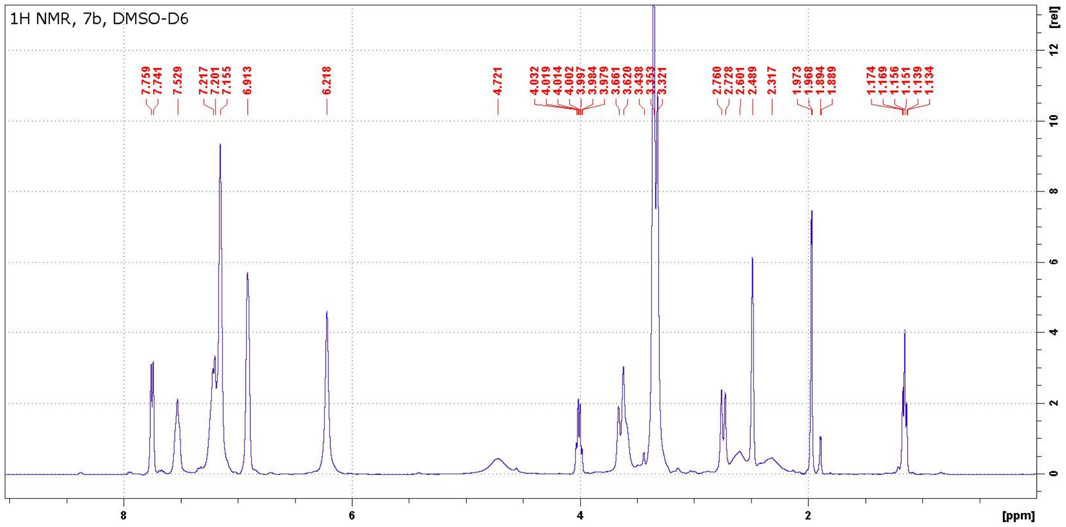

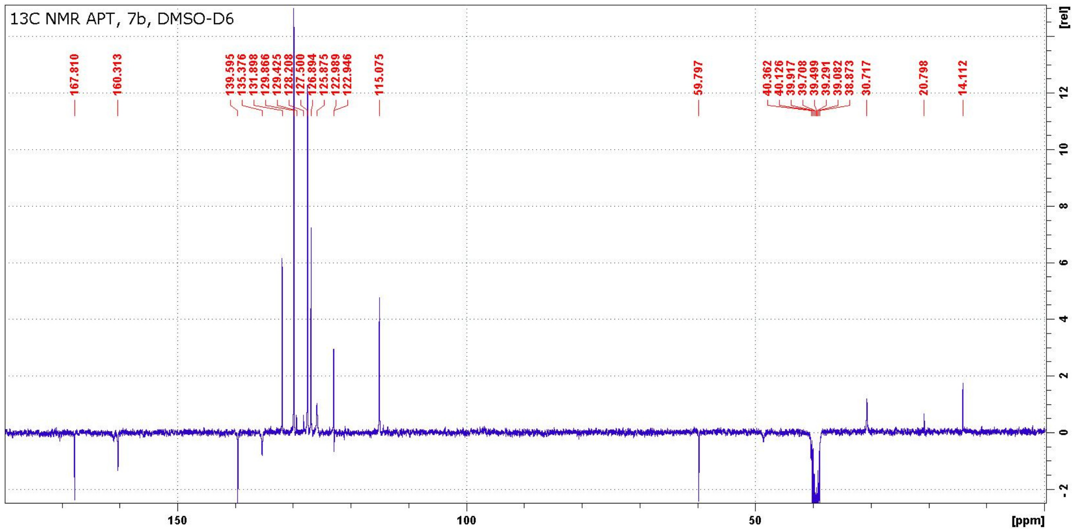

Figure S23: ^{1}H and ^{13}C NMR spectra of compound **7b** (contains ethanol).

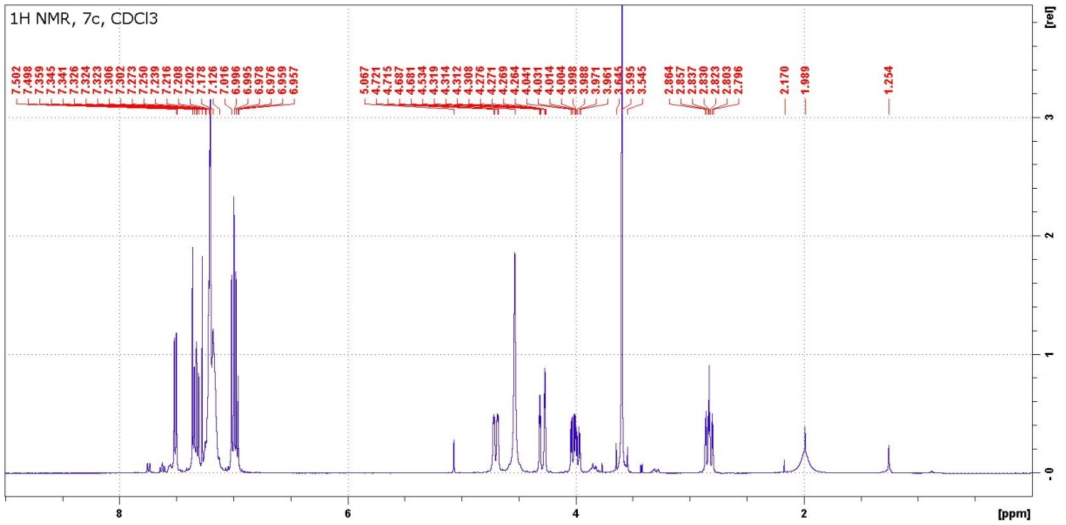

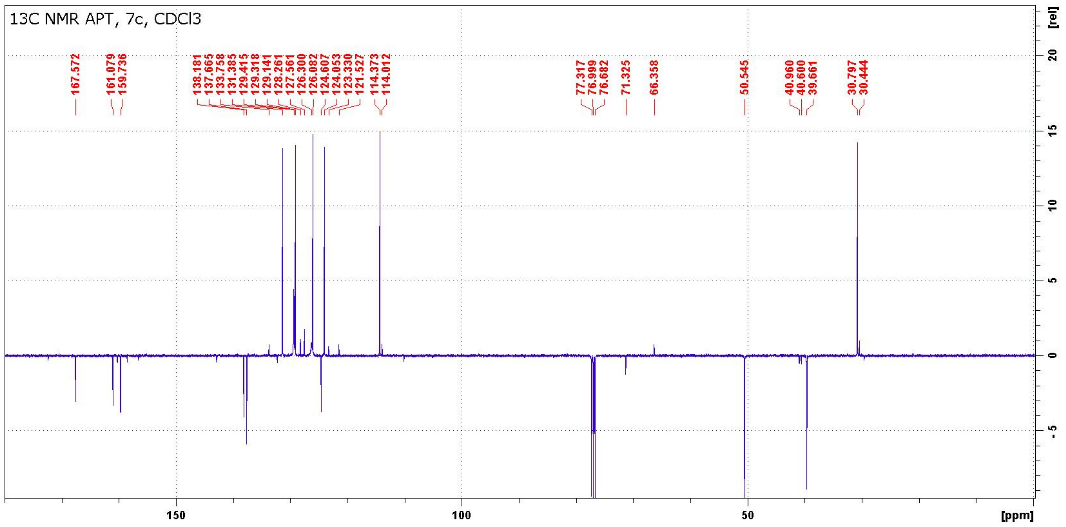

Figure S24: ^{1}H and ^{13}C NMR spectra of compound **7c**.

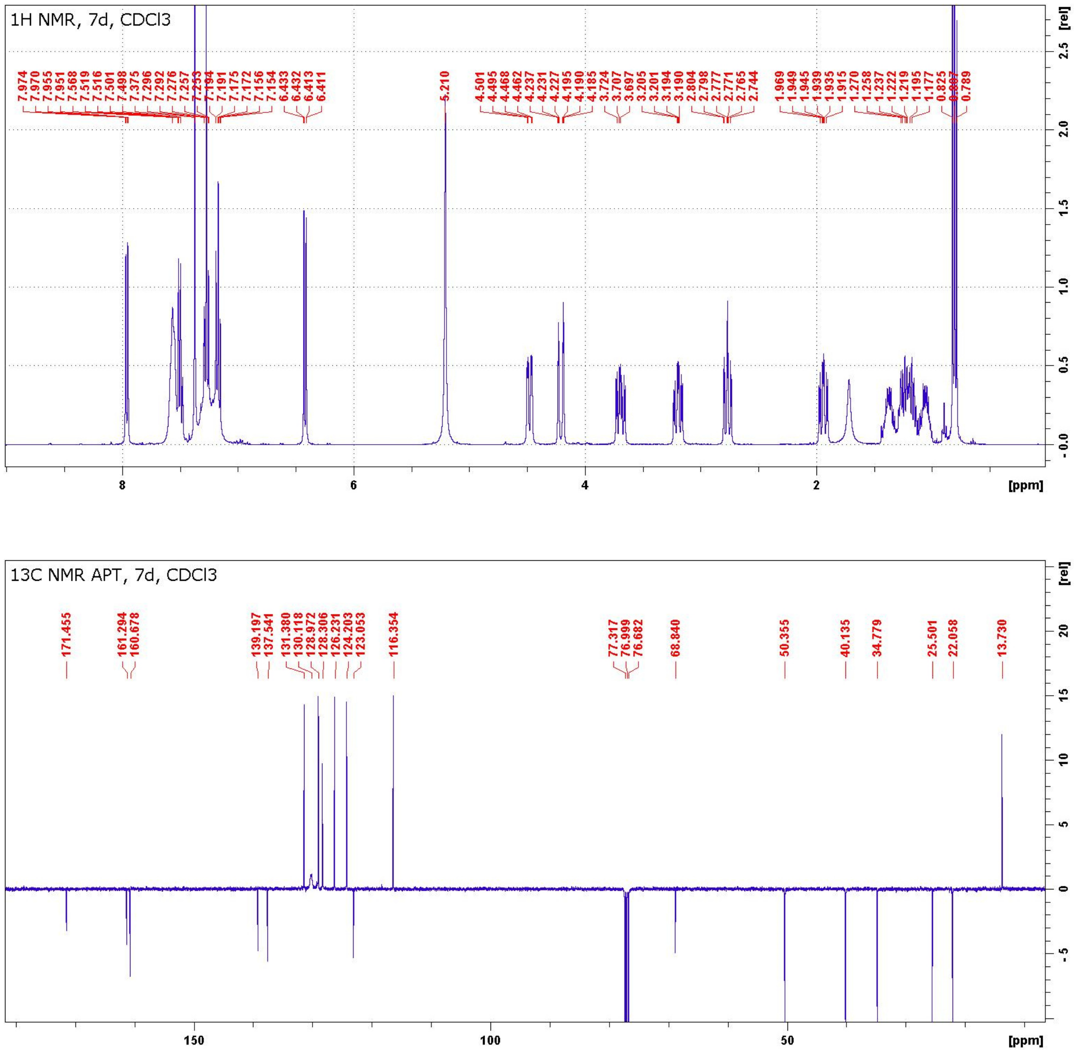

Figure S25: ^{1}H and ^{13}C NMR spectra of compound **7d**.

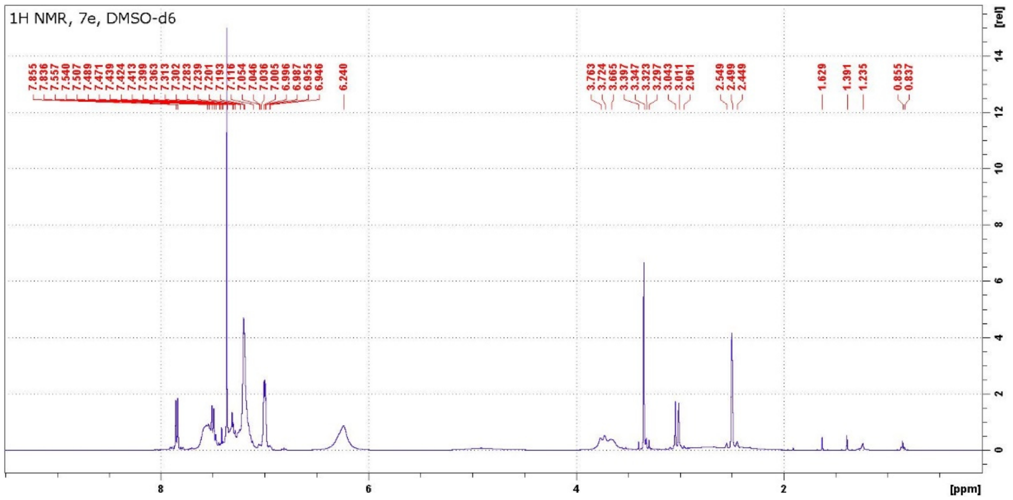

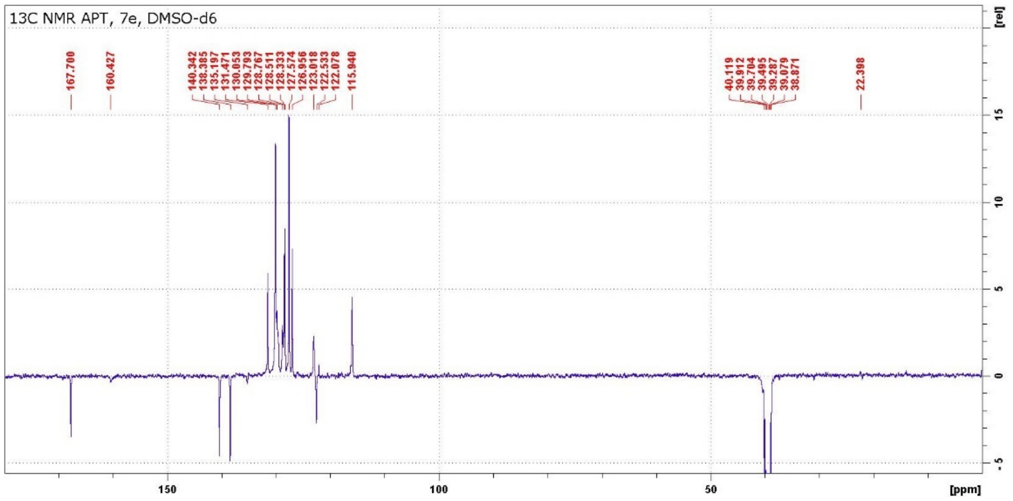

Figure S26: ^{1}H and ^{13}C NMR spectra of compound **7e**.

Comment: Signals of C(4a) in DMSO-d_6 are very broad and they are visible after application of line broadening (lb = 10-20z) as very broad singlets while in $CDCl_3$ solutions, these resonances (compounds **7c** and **7d**) are easily detectable.

Table S7: ^{1}H, ^{13}C and ^{15}N chemical shifts and $^{1}J(^{15}N, ^{1}H)$ coupling constants of compound **8f** in DMSO-d$_6$.

Position	8f		Position	8f	
	δ(H)	δ(C)		δ(H)	δ(C)
**1**	-	-284.5[a]	11-NH	8.68	-292.2[a,d]
**2**	-	158.8	12	7.29	114.9
**3**	11.03	-235.5[a,b]	13	7.32	131.9
**4**	-	172.9	14	6.83	118.1
**5**	5.22	64.3	15	7.57	128.9
**6**	3.38	36.6	1´(R^1)	-	141.5
	3.31	-	2´(R^1)	7.15	119.4
**7**	3.77	39.6	3´(R^1)	7.30	1294
	2.78	-	4´(R^1)	6.97	121.7
**8**	9.44	-269.0[a,c]	1´(R^2)	-	133.8
**9**	-	169.1	2´(R^2)	7.26	127.6
**10**	-	119.0	3´(R^2)	7.36	129.1
**11**	-	144.0	4´(R^2)	7.36	128.8

[a] δ(^{15}N); [b] $^{1}J(^{15}N, ^{1}H)$ = 94.6 Hz; [c] $^{1}J(^{15}N, ^{1}H)$ = 91.5 Hz; [d] $^{1}J(^{15}N, ^{1}H)$ = 89.5 Hz

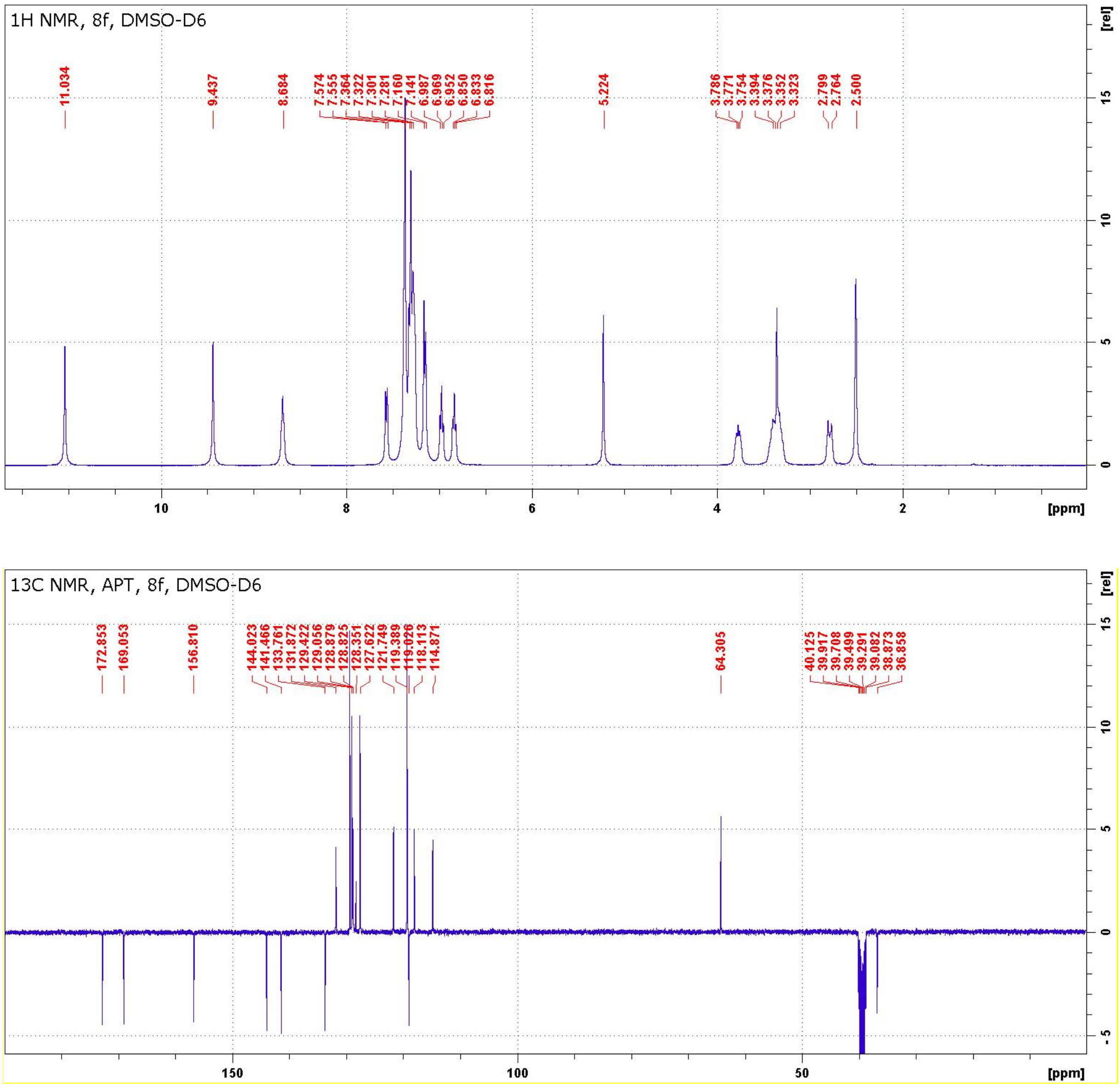

Figure S27: ^{1}H and ^{13}C NMR spectra of compound **8f**.

Crystal data and structure refinement of compounds 5d, 7a, 7b

X-ray crystallography-experimental details

Full-sets of diffraction data for **5d** (yellow) and **7a** (colourless) were collected at 150(2)K with a Bruker D8-Venture diffractometer equipped with Cu (Cu/K_α radiation; λ =1.54178 Å) or Mo (Mo/K_α radiation; λ = 0.71073 Å) microfocus X-ray (IμS) sources, Photon CMOS detector and Oxford Cryosystems cooling device was used for data collection. The frames were integrated with the Bruker SAINT software package using a narrow-frame algorithm. Data were corrected for absorption effects using the Multi-Scan method (SADABS). Obtained data were treated by XT-version 2018/1 and SHELXL-2017/1 software implemented in APEX3 v2016.5-0 (Bruker AXS) system.[1] Hydrogen atoms were mostly localized on a difference Fourier map, however to ensure uniformity of treatment of crystal, all hydrogen atoms were recalculated into idealized positions (riding model) and assigned temperature factors $H_{iso}(H)$ = 1.2 U_{eq} (pivot atom) or of 1.5U_{eq} (methyl). H atoms in methyl, methylene and hydrogen atoms in aromatic rings were placed with C-H distances of 0.98, 0.97 and 0.95Å, respectively, and hydrogen atom of N-H or O-H (H3 fixed in appropriate position) group refined freely. Crystallographic data for structural analysis have been deposited with the Cambridge Crystallographic Data Centre, CCDC nos. 2047078, 2047079 and 2055718, respectively. Copies of this information may be obtained free of charge from The Director, CCDC, 12 Union Road, Cambridge CB2 1EY, UK (fax: +44-1223-336033; e-mail: deposit@ccdc.cam.ac.uk or www: http://www.ccdc.cam.ac.uk).

[1] G. M. Sheldrick, Crystal structure refinement with SHELXL; Acta Cryst. (2015). C71, 3-8 https://doi.org/10.1107/S2053229614024218]

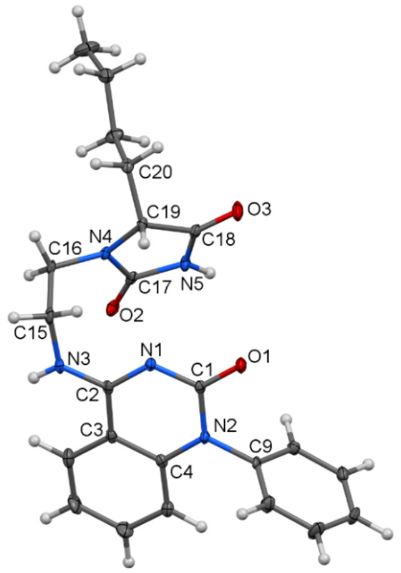

Figure S28: Molecular structure of **5d** – ORTEP view, 40% probability level.

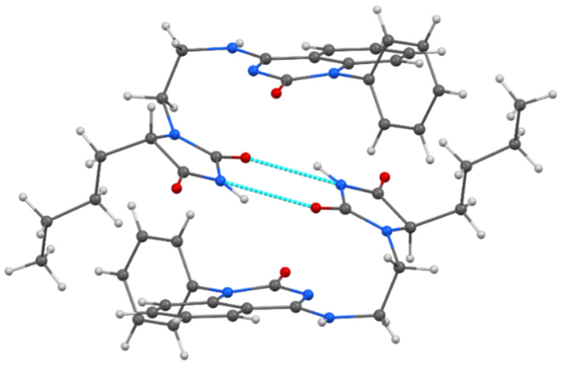

Figure S29: Molecular structure of **5d** – H-bonding representation.

Table S8: Experimental details for **5d**.

Crystal data	
Chemical formula	$C_{23}H_{25}N_5O_3$
M_r	419.48
Crystal system, space group	Monoclinic, $P2_1/c$
Temperature (K)	150
a, *b*, *c* (Å)	15.5947 (11), 9.8133 (7), 14.8551 (12)
β (°)	114.200 (2)
V (Å^3)	2073.6 (3)
Z	4
Radiation type	Mo $K\alpha$
μ (mm^{-1})	0.09
Crystal size (mm)	0.59 × 0.31 × 0.06
**Data collection**	
Diffractometer	Bruker D8 - Venture
Absorption correction	Multi-scan<br>*SADABS2016*/2 - Bruker AXS area detector scaling and absorption correction
T_{min}, T_{max}	0.640, 0.746
No. of measured, independent and observed [$I > 2\sigma(I)$] reflections	45891, 4767, 3859
R_{int}	0.101
$(\sin \theta/\lambda)_{max}$ (Å^{-1})	0.651
**Refinement**	
$R[F^2 > 2\sigma(F^2)]$, $wR(F^2)$, S	0.083, 0.163, 1.25
No. of reflections	4767
No. of parameters	285
No. of restraints	243
H-atom treatment	H atoms treated by a mixture of independent and constrained refinement
$\Delta\rho_{max}$, $\Delta\rho_{min}$ (e Å^{-3})	0.37, −0.41

Computer programs: Bruker Instrument Service vV6.2.3, *APEX3* v2016.5-0 (Bruker AXS), *SAINT* V8.37A (Bruker AXS Inc., 2015), XT, VERSION 2014/5, *SHELXL2014*/7 (Sheldrick, 2014), *PLATON* (Spek, 2009).

Table S9: Geometric parameters (Å, º) for **5d**.

O1-C1	1.232 (3)	C10-C11	1.382 (4)
N1-C2	1.328 (3)	C10-H10	0.9500
N1-C1	1.368 (3)	C11-C12	1.386 (4)
C1-N2	1.398 (3)	C11-H11	0.9500
N2-C4	1.395 (3)	C12-C13	1.379 (4)
N2-C9	1.446 (3)	C12-H12	0.9500
O2-C17	1.226 (3)	C13-C14	1.385 (4)
C2-N3	1.334 (3)	C13-H13	0.9500
C2-C3	1.458 (3)	C14-H14	0.9500
O3-C18	1.209 (3)	C15-C16	1.520 (4)
N3-C15	1.459 (3)	C15-H15A	0.9900
N3-H3	0.80 (3)	C15-H15B	0.9900
C3-C5	1.399 (3)	C16-H16A	0.9900
C3-C4	1.401 (3)	C16-H16B	0.9900
C4-C8	1.398 (4)	C18-C19	1.528 (3)
N4-C17	1.347 (3)	C19-C20	1.531 (3)
N4-C16	1.456 (3)	C19-H19	1.0000
N4-C19	1.462 (3)	C20-C21	1.512 (4)
C5-C6	1.375 (4)	C20-H20A	0.9900
C5-H5	0.9500	C20-H20B	0.9900
N5-C18	1.366 (3)	C21-C22	1.524 (4)
N5-C17	1.391 (3)	C21-H21A	0.9900
N5-H5A	0.8800	C21-H21B	0.9900
C6-C7	1.390 (4)	C22-C23	1.511 (4)
C6-H6	0.9500	C22-H22A	0.9900
C7-C8	1.380 (4)	C22-H22B	0.9900
C7-H7	0.9500	C23-H23A	0.9800
C8-H8	0.9500	C23-H23B	0.9800
C9-C10	1.381 (4)	C23-H23C	0.9800
C9-C14	1.388 (3)		
C2-N1-C1	120.6 (2)	C13-C14-C9	119.2 (2)
O1-C1-N1	122.0 (2)	C13-C14-H14	120.4
O1-C1-N2	118.9 (2)	C9-C14-H14	120.4
N1-C1-N2	119.1 (2)	N3-C15-C16	111.6 (2)
C4-N2-C1	121.8 (2)	N3-C15-H15A	109.3
C4-N2-C9	118.7 (2)	C16-C15-H15A	109.3
C1-N2-C9	119.28 (19)	N3-C15-H15B	109.3
N1-C2-N3	118.9 (2)	C16-C15-H15B	109.3
N1-C2-C3	122.4 (2)	H15A-C15-H15B	108.0
N3-C2-C3	118.7 (2)	N4-C16-C15	113.4 (2)
C2-N3-C15	125.4 (2)	N4-C16-H16A	108.9
C2-N3-H3	119 (2)	C15-C16-H16A	108.9
C15-N3-H3	115 (2)	N4-C16-H16B	108.9
C5-C3-C4	119.2 (2)	C15-C16-H16B	108.9
C5-C3-C2	124.2 (2)	H16A-C16-H16B	107.7
C4-C3-C2	116.6 (2)	O2-C17-N4	127.7 (2)

N2-C4-C8	121.6 (2)	O2-C17-N5	124.4 (2)
N2-C4-C3	118.7 (2)	N4-C17-N5	107.9 (2)
C8-C4-C3	119.7 (2)	O3-C18-N5	127.4 (2)
C17-N4-C16	123.3 (2)	O3-C18-C19	125.9 (2)
C17-N4-C19	111.79 (19)	N5-C18-C19	106.67 (19)
C16-N4-C19	121.7 (2)	N4-C19-C18	101.41 (19)
C6-C5-C3	120.7 (2)	N4-C19-C20	113.99 (19)
C6-C5-H5	119.7	C18-C19-C20	112.6 (2)
C3-C5-H5	119.7	N4-C19-H19	109.5
C18-N5-C17	111.8 (2)	C18-C19-H19	109.5
C18-N5-H5A	124.1	C20-C19-H19	109.5
C17-N5-H5A	124.1	C21-C20-C19	113.5 (2)
C5-C6-C7	119.8 (2)	C21-C20-H20A	108.9
C5-C6-H6	120.1	C19-C20-H20A	108.9
C7-C6-H6	120.1	C21-C20-H20B	108.9
C8-C7-C6	120.7 (3)	C19-C20-H20B	108.9
C8-C7-H7	119.7	H20A-C20-H20B	107.7
C6-C7-H7	119.7	C20-C21-C22	114.8 (2)
C7-C8-C4	119.8 (2)	C20-C21-H21A	108.6
C7-C8-H8	120.1	C22-C21-H21A	108.6
C4-C8-H8	120.1	C20-C21-H21B	108.6
C10-C9-C14	120.7 (2)	C22-C21-H21B	108.6
C10-C9-N2	119.4 (2)	H21A-C21-H21B	107.6
C14-C9-N2	119.9 (2)	C23-C22-C21	112.6 (2)
C9-C10-C11	119.5 (3)	C23-C22-H22A	109.1
C9-C10-H10	120.2	C21-C22-H22A	109.1
C11-C10-H10	120.2	C23-C22-H22B	109.1
C10-C11-C12	120.3 (3)	C21-C22-H22B	109.1
C10-C11-H11	119.8	H22A-C22-H22B	107.8
C12-C11-H11	119.8	C22-C23-H23A	109.5
C13-C12-C11	119.8 (2)	C22-C23-H23B	109.5
C13-C12-H12	120.1	H23A-C23-H23B	109.5
C11-C12-H12	120.1	C22-C23-H23C	109.5
C12-C13-C14	120.5 (2)	H23A-C23-H23C	109.5
C12-C13-H13	119.7	H23B-C23-H23C	109.5
C14-C13-H13	119.7		

Table 10: Hydrogen-bond geometry (Å, º) for **5d**.

D—H···*A*	*D*—H	H···*A*	*D*···*A*	*D*—H···*A*
N3—H3···O1[i]	0.80 (3)	2.29 (3)	3.080 (3)	167 (3)
N5—H5*A*···O2[ii]	0.88	2.07	2.858 (3)	148

Symmetry codes: (i) *x*, −*y*+1/2, *z*−1/2; (ii) −*x*+1, −*y*+1, −*z*+1.

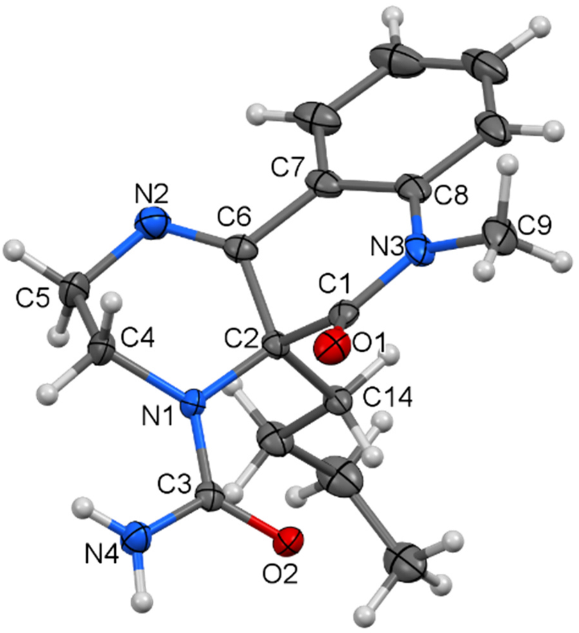

Figure S30: Molecular structure of **7a** – ORTEP view, 40% probability level.

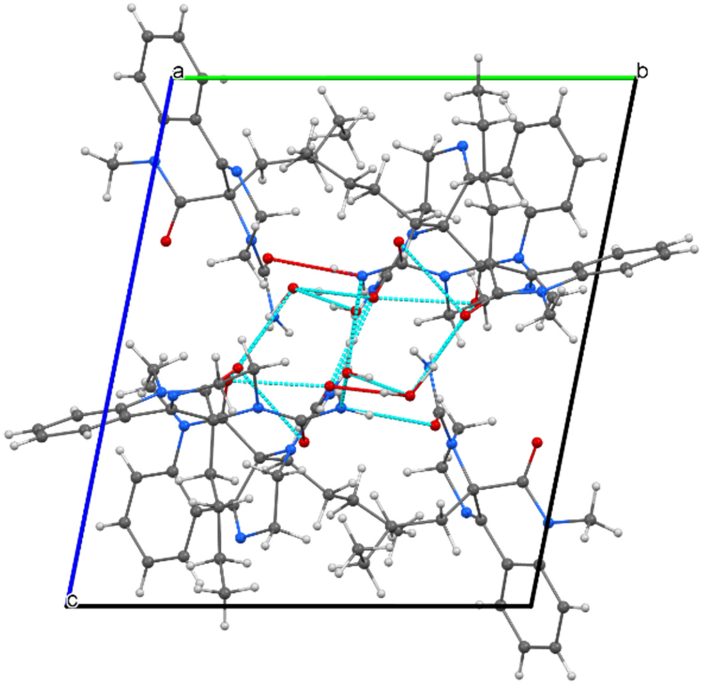

Figure S31: Molecular structure of **7a** – H-bonding representation (left) supramolecular architecture, view along c axis.

Table S11: Experimental details for **7a**.

Crystal data	
Chemical formula	$C_{17}H_{22}N_4O_2 \cdot 0.67(H_2O)$
M_r	326.40C
Crystal system, space group	Triclinic, *P*-1
Temperature (K)	150
a, *b*, *c* (Å)	13.0440 (4), 13.4973 (5), 16.1736 (6)
α, β, γ (°)	95.036 (2), 110.468 (2), 104.772 (2)
V ($Å^3$)	2529.88 (16)
Z	6
Radiation type	Mo *K*α
μ (mm^{-1})	0.09
Crystal size (mm)	0.74 × 0.42 × 0.38
Data collection	
Diffractometer	Bruker D8 - Venture
Absorption correction	Multi-scan<br>*SADABS2016*/2 - Bruker AXS area detector scaling and absorption correction
T_{min}, T_{max}	0.684, 0.746
No. of measured, independent and observed [$I > 2\sigma(I)$] reflections	72323, 11662, 9083
R_{int}	0.049
$(\sin \theta/\lambda)_{max}$ ($Å^{-1}$)	0.652
Refinement	
$R[F^2 > 2\sigma(F^2)]$, $wR(F^2)$, S	0.056, 0.148, 1.04
No. of reflections	11662
No. of parameters	665
No. of restraints	7
H-atom treatment	H atoms treated by a mixture of independent and constrained refinement
$\Delta\rho_{max}$, $\Delta\rho_{min}$ (e $Å^{-3}$)	1.21, −0.53

Computer programs: Bruker Instrument Service vV6.2.3, *APEX3* v2016.5-0 (Bruker AXS), *SAINT* V8.37A (Bruker AXS Inc., 2015), XT, VERSION 2014/5'_computing_structure_refinement '*SHELXL2017*/1 (Sheldrick, 2017), *PLATON* (Spek, 2009).

Table S12: Geometric parameters (Å, º) for **7a**.

O1-C1	1.214 (2)	N3A-C9A	1.468 (2)
C1-N3	1.379 (2)	O2W-H3	0.967 (9)
C1-C2	1.543 (2)	O2W-H5	0.944 (10)
N1-C3	1.381 (2)	C4A-C5A	1.502 (3)
N1-C2	1.460 (2)	C4A-H4AA	0.9900
N1-C4	1.461 (2)	C4A-H4AC	0.9900
O2-C3	1.236 (2)	N4A-H4AD	0.8800
C2-C6	1.524 (3)	N4A-H4AE	0.8800
C2-C14	1.563 (2)	N3B-C8B	1.415 (2)
N2-C6	1.264 (2)	N3B-C9B	1.467 (2)
N2-C5	1.459 (3)	C3B-N4B	1.335 (2)
C3-N4	1.350 (2)	C5A-H5AA	0.9900
N3-C8	1.409 (3)	C5A-H5AC	0.9900
N3-C9	1.463 (3)	N4B-H4BA	0.8800
C4-C5	1.506 (3)	N4B-H4BB	0.8800
C4-H4A	0.9900	C4B-C5B	1.507 (3)
C4-H4AB	0.9900	C4B-H4BC	0.9900
N1B-C3B	1.378 (2)	C4B-H4BD	0.9900
N1B-C2B	1.461 (2)	C6A-C7A	1.475 (3)
N1B-C4B	1.461 (2)	C5B-H5BA	0.9900
O1B-C1B	1.220 (2)	C5B-H5BB	0.9900
C1B-N3B	1.364 (2)	C7A-C10A	1.387 (3)
C1B-C2B	1.548 (2)	C7A-C8A	1.404 (3)
C1A-O1A	1.219 (2)	C6B-C7B	1.476 (2)
C1A-N3A	1.355 (3)	C8A-C13A	1.398 (3)
C1A-C2A	1.549 (3)	C7B-C10B	1.387 (3)
N1A-C3A	1.379 (2)	C7B-C8B	1.400 (2)
N1A-C2A	1.458 (3)	C9A-H9AA	0.9800
N1A-C4A	1.468 (3)	C9A-H9AB	0.9800
N4-H4'	0.85 (3)	C9A-H9AC	0.9800
N4-H4	0.87 (3)	C8B-C13B	1.394 (3)
C5-H5A	0.9900	C10A-C11A	1.386 (3)
C5-H5AB	0.9900	C10A-H10A	0.9500
C6-C7	1.484 (3)	C9B-H9BA	0.9800
C7-C10	1.391 (3)	C9B-H9BB	0.9800
C7-C8	1.398 (3)	C9B-H9BC	0.9800
C8-C13	1.400 (3)	C11A-C12A	1.381 (3)
C9-H9A	0.9800	C11A-H11A	0.9500
C9-H9B	0.9800	C10B-C11B	1.383 (3)
C9-H9C	0.9800	C10B-H10B	0.9500
N2B-C6B	1.267 (2)	C12A-C13A	1.377 (3)
N2B-C5B	1.455 (2)	C12A-H12A	0.9500
O2B-C3B	1.247 (2)	C11B-C12B	1.376 (3)
C2B-C6B	1.527 (2)	C11B-H11B	0.9500
C2B-C14B	1.558 (2)	C13A-H13A	0.9500
C2A-C6A	1.532 (2)	C12B-C13B	1.384 (3)

C2A-C14A	1.559 (3)	C12B-H12B	0.9500
N2A-C6A	1.273 (2)	C14A-C15A	1.519 (3)
N2A-C5A	1.459 (3)	C14A-H14C	0.9900
O2A-C3A	1.229 (2)	C14A-H14D	0.9900
C10-C11	1.389 (3)	C13B-H13B	0.9500
C10-H10	0.9500	C15A-C16A	1.521 (3)
C11-C12	1.380 (4)	C15A-H15C	0.9900
C11-H11	0.9500	C15A-H15D	0.9900
C13-C12	1.375 (4)	C14B-C15B	1.515 (2)
C13-H13	0.9500	C14B-H14E	0.9900
C14-C15	1.519 (2)	C14B-H14F	0.9900
C14-H14A	0.9900	C16A-C17A	1.519 (3)
C14-H14B	0.9900	C16A-H16C	0.9900
C15-C16	1.529 (3)	C16A-H16D	0.9900
C15-H15A	0.9900	C15B-C16B	1.520 (3)
C15-H15B	0.9900	C15B-H15E	0.9900
C16-C17	1.499 (4)	C15B-H15F	0.9900
C16-H16A	0.9900	C17A-H17D	0.9800
C16-H16B	0.9900	C17A-H17E	0.9800
C17-H17A	0.9800	C17A-H17F	0.9800
C17-H17B	0.9800	C17B-C16B	1.508 (3)
C17-H17C	0.9800	C17B-H17G	0.9800
O1W-H1	1.0669 (17)	C17B-H17H	0.9800
O1W-H2	0.967 (10)	C17B-H17I	0.9800
C12-H12	0.9500	C16B-H16E	0.9900
C3A-N4A	1.361 (3)	C16B-H16F	0.9900
N3A-C8A	1.417 (3)		
O1-C1-N3	122.99 (17)	C1B-N3B-C9B	117.75 (16)
O1-C1-C2	121.79 (16)	C8B-N3B-C9B	118.90 (16)
N3-C1-C2	115.21 (16)	O2B-C3B-N4B	120.72 (16)
C3-N1-C2	119.96 (14)	O2B-C3B-N1B	121.26 (16)
C3-N1-C4	122.56 (15)	N4B-C3B-N1B	117.88 (15)
C2-N1-C4	114.99 (14)	N2A-C5A-C4A	111.48 (17)
N1-C2-C6	111.79 (14)	N2A-C5A-H5AA	109.3
N1-C2-C1	110.13 (14)	C4A-C5A-H5AA	109.3
C6-C2-C1	106.38 (14)	N2A-C5A-H5AC	109.3
N1-C2-C14	112.54 (13)	C4A-C5A-H5AC	109.3
C6-C2-C14	106.21 (14)	H5AA-C5A-H5AC	108.0
C1-C2-C14	109.55 (14)	C3B-N4B-H4BA	120.0
C6-N2-C5	116.47 (17)	C3B-N4B-H4BB	120.0
O2-C3-N4	120.07 (17)	H4BA-N4B-H4BB	120.0
O2-C3-N1	122.28 (16)	N1B-C4B-C5B	109.07 (15)
N4-C3-N1	117.62 (16)	N1B-C4B-H4BC	109.9
C1-N3-C8	121.41 (16)	C5B-C4B-H4BC	109.9
C1-N3-C9	118.12 (18)	N1B-C4B-H4BD	109.9
C8-N3-C9	119.87 (17)	C5B-C4B-H4BD	109.9
N1-C4-C5	109.62 (16)	H4BC-C4B-H4BD	108.3
N1-C4-H4A	109.7	N2A-C6A-C7A	119.71 (16)

C5-C4-H4A	109.7	N2A-C6A-C2A	127.85 (18)
N1-C4-H4AB	109.7	C7A-C6A-C2A	112.43 (15)
C5-C4-H4AB	109.7	N2B-C5B-C4B	112.37 (15)
H4A-C4-H4AB	108.2	N2B-C5B-H5BA	109.1
C3B-N1B-C2B	120.10 (14)	C4B-C5B-H5BA	109.1
C3B-N1B-C4B	120.14 (15)	N2B-C5B-H5BB	109.1
C2B-N1B-C4B	115.11 (13)	C4B-C5B-H5BB	109.1
O1B-C1B-N3B	122.58 (16)	H5BA-C5B-H5BB	107.9
O1B-C1B-C2B	120.87 (16)	C10A-C7A-C8A	119.13 (18)
N3B-C1B-C2B	116.52 (15)	C10A-C7A-C6A	121.89 (16)
O1A-C1A-N3A	122.70 (18)	C8A-C7A-C6A	118.96 (17)
O1A-C1A-C2A	120.63 (19)	N2B-C6B-C7B	119.19 (16)
N3A-C1A-C2A	116.64 (17)	N2B-C6B-C2B	127.82 (16)
C3A-N1A-C2A	120.97 (16)	C7B-C6B-C2B	112.97 (14)
C3A-N1A-C4A	122.70 (17)	C13A-C8A-C7A	119.28 (18)
C2A-N1A-C4A	113.31 (15)	C13A-C8A-N3A	121.10 (17)
C3-N4-H4'	122.1 (17)	C7A-C8A-N3A	119.58 (18)
C3-N4-H4	114.8 (16)	C10B-C7B-C8B	119.56 (17)
H4'-N4-H4	122 (2)	C10B-C7B-C6B	121.31 (16)
N2-C5-C4	113.11 (16)	C8B-C7B-C6B	119.11 (16)
N2-C5-H5A	109.0	N3A-C9A-H9AA	109.5
C4-C5-H5A	109.0	N3A-C9A-H9AB	109.5
N2-C5-H5AB	109.0	H9AA-C9A-H9AB	109.5
C4-C5-H5AB	109.0	N3A-C9A-H9AC	109.5
H5A-C5-H5AB	107.8	H9AA-C9A-H9AC	109.5
N2-C6-C7	119.95 (18)	H9AB-C9A-H9AC	109.5
N2-C6-C2	128.03 (17)	C13B-C8B-C7B	119.40 (17)
C7-C6-C2	111.95 (16)	C13B-C8B-N3B	120.79 (17)
C10-C7-C8	119.57 (19)	C7B-C8B-N3B	119.75 (16)
C10-C7-C6	120.91 (19)	C11A-C10A-C7A	121.29 (18)
C8-C7-C6	119.51 (18)	C11A-C10A-H10A	119.4
C7-C8-C13	119.4 (2)	C7A-C10A-H10A	119.4
C7-C8-N3	119.83 (17)	N3B-C9B-H9BA	109.5
C13-C8-N3	120.8 (2)	N3B-C9B-H9BB	109.5
N3-C9-H9A	109.5	H9BA-C9B-H9BB	109.5
N3-C9-H9B	109.5	N3B-C9B-H9BC	109.5
H9A-C9-H9B	109.5	H9BA-C9B-H9BC	109.5
N3-C9-H9C	109.5	H9BB-C9B-H9BC	109.5
H9A-C9-H9C	109.5	C12A-C11A-C10A	119.1 (2)
H9B-C9-H9C	109.5	C12A-C11A-H11A	120.5
C6B-N2B-C5B	116.85 (15)	C10A-C11A-H11A	120.5
N1B-C2B-C6B	111.02 (13)	C11B-C10B-C7B	120.77 (18)
N1B-C2B-C1B	109.49 (14)	C11B-C10B-H10B	119.6
C6B-C2B-C1B	107.80 (13)	C7B-C10B-H10B	119.6
N1B-C2B-C14B	113.26 (14)	C13A-C12A-C11A	120.9 (2)
C6B-C2B-C14B	106.08 (14)	C13A-C12A-H12A	119.5
C1B-C2B-C14B	109.01 (13)	C11A-C12A-H12A	119.5
N1A-C2A-C6A	110.41 (15)	C12B-C11B-C10B	119.48 (19)

N1A-C2A-C1A	111.61 (16)	C12B-C11B-H11B	120.3
C6A-C2A-C1A	106.68 (14)	C10B-C11B-H11B	120.3
N1A-C2A-C14A	112.15 (15)	C12A-C13A-C8A	120.25 (19)
C6A-C2A-C14A	107.20 (15)	C12A-C13A-H13A	119.9
C1A-C2A-C14A	108.54 (15)	C8A-C13A-H13A	119.9
C6A-N2A-C5A	117.45 (16)	C11B-C12B-C13B	120.90 (19)
C11-C10-C7	120.5 (2)	C11B-C12B-H12B	119.5
C11-C10-H10	119.7	C13B-C12B-H12B	119.5
C7-C10-H10	119.7	C15A-C14A-C2A	113.95 (16)
C12-C11-C10	119.5 (2)	C15A-C14A-H14C	108.8
C12-C11-H11	120.3	C2A-C14A-H14C	108.8
C10-C11-H11	120.3	C15A-C14A-H14D	108.8
C12-C13-C8	120.1 (2)	C2A-C14A-H14D	108.8
C12-C13-H13	119.9	H14C-C14A-H14D	107.7
C8-C13-H13	119.9	C12B-C13B-C8B	119.88 (19)
C15-C14-C2	113.23 (15)	C12B-C13B-H13B	120.1
C15-C14-H14A	108.9	C8B-C13B-H13B	120.1
C2-C14-H14A	108.9	C14A-C15A-C16A	111.14 (17)
C15-C14-H14B	108.9	C14A-C15A-H15C	109.4
C2-C14-H14B	108.9	C16A-C15A-H15C	109.4
H14A-C14-H14B	107.7	C14A-C15A-H15D	109.4
C14-C15-C16	111.55 (17)	C16A-C15A-H15D	109.4
C14-C15-H15A	109.3	H15C-C15A-H15D	108.0
C16-C15-H15A	109.3	C15B-C14B-C2B	112.86 (14)
C14-C15-H15B	109.3	C15B-C14B-H14E	109.0
C16-C15-H15B	109.3	C2B-C14B-H14E	109.0
H15A-C15-H15B	108.0	C15B-C14B-H14F	109.0
C17-C16-C15	114.17 (19)	C2B-C14B-H14F	109.0
C17-C16-H16A	108.7	H14E-C14B-H14F	107.8
C15-C16-H16A	108.7	C17A-C16A-C15A	113.1 (2)
C17-C16-H16B	108.7	C17A-C16A-H16C	109.0
C15-C16-H16B	108.7	C15A-C16A-H16C	109.0
H16A-C16-H16B	107.6	C17A-C16A-H16D	109.0
C16-C17-H17A	109.5	C15A-C16A-H16D	109.0
C16-C17-H17B	109.5	H16C-C16A-H16D	107.8
H17A-C17-H17B	109.5	C14B-C15B-C16B	112.89 (16)
C16-C17-H17C	109.5	C14B-C15B-H15E	109.0
H17A-C17-H17C	109.5	C16B-C15B-H15E	109.0
H17B-C17-H17C	109.5	C14B-C15B-H15F	109.0
H1-O1W-H2	106 (2)	C16B-C15B-H15F	109.0
C13-C12-C11	120.9 (2)	H15E-C15B-H15F	107.8
C13-C12-H12	119.5	C16A-C17A-H17D	109.5
C11-C12-H12	119.5	C16A-C17A-H17E	109.5
O2A-C3A-N4A	120.36 (17)	H17D-C17A-H17E	109.5
O2A-C3A-N1A	122.88 (19)	C16A-C17A-H17F	109.5
N4A-C3A-N1A	116.76 (17)	H17D-C17A-H17F	109.5
C1A-N3A-C8A	122.24 (16)	H17E-C17A-H17F	109.5
C1A-N3A-C9A	118.57 (17)	C16B-C17B-H17G	109.5

C8A-N3A-C9A	118.51 (17)	C16B-C17B-H17H	109.5
H3-O2W-H5	114.3 (17)	H17G-C17B-H17H	109.5
N1A-C4A-C5A	109.35 (17)	C16B-C17B-H17I	109.5
N1A-C4A-H4AA	109.8	H17G-C17B-H17I	109.5
C5A-C4A-H4AA	109.8	H17H-C17B-H17I	109.5
N1A-C4A-H4AC	109.8	C17B-C16B-C15B	111.76 (19)
C5A-C4A-H4AC	109.8	C17B-C16B-H16E	109.3
H4AA-C4A-H4AC	108.3	C15B-C16B-H16E	109.3
C3A-N4A-H4AD	120.0	C17B-C16B-H16F	109.3
C3A-N4A-H4AE	120.0	C15B-C16B-H16F	109.3
H4AD-N4A-H4AE	120.0	H16E-C16B-H16F	107.9
C1B-N3B-C8B	122.70 (14)		

Table S13: Hydrogen-bond geometry (Å, º) for **7a**.

D—H···*A*	*D*—H	H···*A*	*D*···*A*	*D*—H···*A*
O1*W*—H1···O1*A*	1.07 (1)	1.81 (1)	2.777 (2)	149 (1)
O1*W*—H2···O2*B*	0.97 (1)	1.80 (1)	2.755 (2)	170 (3)
O2*W*—H3···O1*W*	0.97 (1)	1.78 (1)	2.742 (3)	171 (2)
N4—H4′···O1*B*	0.85 (3)	2.31 (3)	3.039 (2)	144 (2)
N4—H4′···O2*B*	0.85 (3)	2.58 (2)	3.079 (2)	119 (2)
N4—H4···O2*W*	0.87 (3)	2.32 (3)	3.170 (3)	164 (2)
C5—H5*AB*···O1*W*^{i}	0.99	2.58	3.362 (3)	135
O2*W*—H5···N4*A*	0.94 (1)	2.00 (3)	2.932 (2)	168 (9)
C9—H9*B*···N2*B*^{ii}	0.98	2.54	3.460 (3)	156
C14—H14*B*···O2	0.99	2.21	2.801 (2)	117
C4*A*—H4*AC*···O2*W*^{iii}	0.99	2.33	3.253 (3)	155
N4*A*—H4*AD*···O2	0.88	2.09	2.966 (2)	173
N4*A*—H4*AE*···O2*W*^{iii}	0.88	2.09	2.942 (2)	162
N4*B*—H4*BA*···O2*B*^{i}	0.88	2.13	2.977 (2)	161
N4*B*—H4*BB*···O2*A*^{i}	0.88	2.27	3.142 (2)	171
C4*B*—H4*BC*···O2*A*^{i}	0.99	2.28	3.084 (2)	138
C9*A*—H9*AA*···N2*A*^{iv}	0.98	2.52	3.482 (3)	166
C9*A*—H9*AB*···O1^{v}	0.98	2.60	3.496 (3)	151
C9*A*—H9*AC*···O1*B*^{i}	0.98	2.60	3.276 (2)	126
C13*A*—H13*A*···O1^{v}	0.95	2.35	3.202 (2)	149
C14*A*—H14*D*···O2*A*	0.99	2.24	2.848 (3)	118
C14*B*—H14*E*···O2*B*	0.99	2.31	2.869 (2)	114

Symmetry codes: (i) $-x+1, -y+1, -z+1$; (ii) $-x+1, -y+2, -z+1$; (iii) $-x, -y+1, -z+1$; (iv) $-x, -y, -z+1$; (v) $x, y-1, z$.

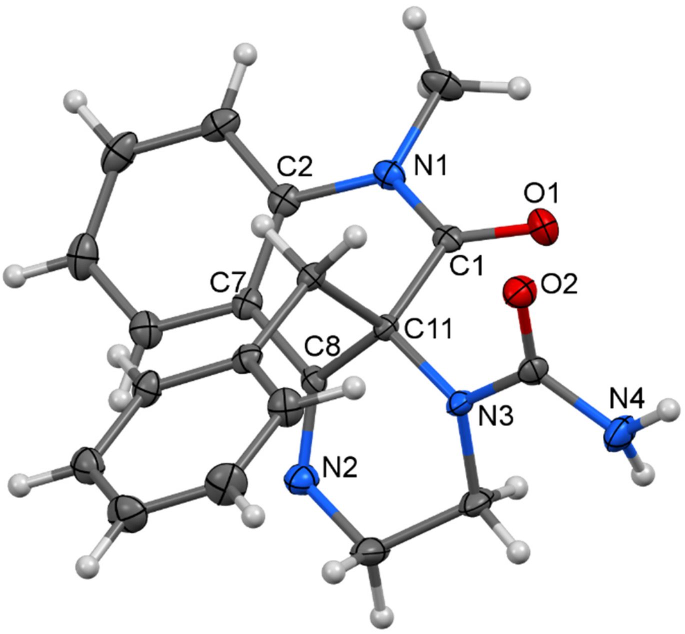

Figure S32: Molecular structure of **7b** – ORTEP view, 40% probability level

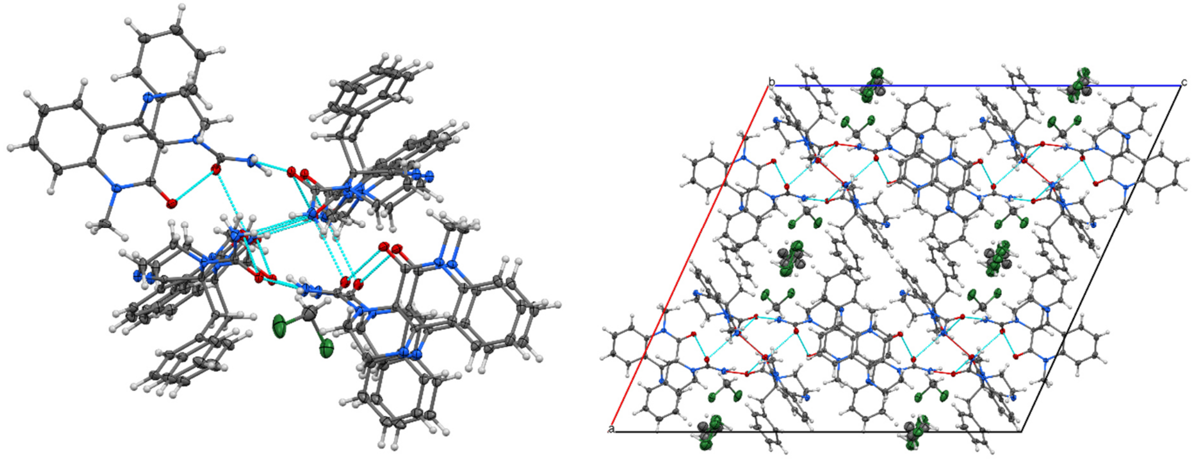

Figure S33: Molecular structure of **7b** – H-bonding representation (left) supramolecular architecture, view along b axis

Table S14: Experimental details for **7b**.

Crystal data	
Chemical formula	$2(C_{20}H_{20}N_4O_2)\cdot 1.5(CH_2Cl_2)$
M_r	824.19
Crystal system, space group	Monoclinic, *C*2/*c*
Temperature (K)	150
a, *b*, *c* (Å)	28.5456 (10), 9.6974 (3), 30.9033 (11)
β (°)	114.810 (1)
V (Å^3)	7765.0 (5)
Z	8
Radiation type	Mo *K*α
μ (mm^{-1})	0.29
Crystal size (mm)	0.59 × 0.43 × 0.13
**Data collection**	
Diffractometer	Bruker D8 - Venture
Absorption correction	Multi-scan *SADABS2016*/2 - Bruker AXS area detector scaling and absorption correction
No. of measured, independent and observed [$I > 2\sigma(I)$] reflections	90897, 8953, 7534
R_{int}	0.040
$(\sin \theta/\lambda)_{max}$ (Å^{-1})	0.651
**Refinement**	
$R[F^2 > 2\sigma(F^2)]$, $wR(F^2)$, S	0.054, 0.239, 1.06
No. of reflections	8953
No. of parameters	545
No. of restraints	20
H-atom treatment	H atoms treated by a mixture of independent and constrained refinement
$\Delta\rho_{max}$, $\Delta\rho_{min}$ (e Å^{-3})	0.96, −1.04

Computer programs: Bruker Instrument Service vV6.2.3, *APEX3* v2016.5-0 (Bruker AXS), *SAINT* V8.37A (Bruker AXS Inc., 2015), XT, VERSION 2014/5'_computing_structure_refinement '*SHELXL2017*/1 (Sheldrick, 2017), *PLATON* (Spek, 2009).

Table S15: Geometric parameters (Å, º) for **7b**.

Cl1—C21	1.756 (3)	C18—H18	0.9500
O1—C1	1.214 (2)	C19—C20	1.396 (3)
N1—C1	1.365 (2)	C19—H19	0.9500
N1—C2	1.418 (2)	C20—H20	0.9500
N1—C12	1.471 (2)	C21—H21A	0.9900
C1—C11	1.559 (2)	C21—H21B	0.9900
Cl2—C21	1.754 (3)	C101—C111	1.547 (2)
O2—C13	1.240 (2)	C102—C107	1.398 (2)
N2—C8	1.269 (2)	C102—C103	1.400 (2)
N2—C9	1.461 (3)	C103—C104	1.382 (3)
C2—C3	1.391 (3)	C103—H103	0.9500
C2—C7	1.399 (3)	C104—C105	1.383 (3)
O3—C101	1.232 (2)	C104—H104	0.9500
N3—C13	1.373 (2)	C105—C106	1.381 (3)
N3—C11	1.455 (2)	C105—H105	0.9500
N3—C10	1.459 (2)	C106—C107	1.391 (2)
C3—C4	1.385 (3)	C106—H106	0.9500
C3—H3	0.9500	C107—C108	1.482 (2)
N4—C13	1.362 (2)	C108—C111	1.535 (2)
N4—H4A	0.96 (3)	C109—C110	1.509 (3)
N4—H4B	0.86 (3)	C109—H10C	0.9900
O4—C113	1.227 (2)	C109—H10D	0.9900
C4—C5	1.392 (3)	C110—H11A	0.9900
C4—H4	0.9500	C110—H11B	0.9900
N5—C101	1.361 (2)	C111—C114	1.577 (2)
N5—C102	1.414 (2)	C112—H11C	0.9800
N5—C112	1.469 (2)	C112—H11D	0.9800
C5—C6	1.373 (3)	C112—H11E	0.9800
C5—H5	0.9500	C114—C115	1.508 (2)
N6—C108	1.267 (2)	C114—H11F	0.9900
N6—C109	1.460 (2)	C114—H11G	0.9900
C6—C7	1.395 (2)	C115—C120	1.389 (3)
C6—H6	0.9500	C115—C116	1.394 (3)
N7—C113	1.385 (2)	C116—C117	1.385 (3)
N7—C111	1.454 (2)	C116—H116	0.9500
N7—C110	1.464 (2)	C117—C118	1.381 (3)
C7—C8	1.479 (3)	C117—H117	0.9500
N8—C113	1.359 (2)	C118—C119	1.376 (3)
N8—H8A	0.82 (3)	C118—H118	0.9500
N8—H8B	0.86 (3)	C119—C120	1.389 (3)

C8—C11	1.531 (2)	C119—H119	0.9500
C9—C10	1.512 (3)	C120—H120	0.9500
C9—H9A	0.9900	C22—C22^{i}	1.12 (3)
C9—H9B	0.9900	C22—Cl3^{i}	1.124 (18)
C10—H10A	0.9900	C22—Cl4	1.730 (12)
C10—H10B	0.9900	C22—Cl3	1.748 (12)
C11—C14	1.566 (2)	C22—H22A	0.979 (9)
C12—H12A	0.9800	C22—H22B	0.977 (9)
C12—H12B	0.9800	Cl3—Cl3^{i}	1.95 (4)
C12—H12C	0.9800	Cl3—H22D^{i}	0.83 (5)
C14—C15	1.508 (2)	C22A—C22A^{i}	1.53 (3)
C14—H14A	0.9900	C22A—Cl3A^{i}	1.56 (2)
C14—H14B	0.9900	C22A—Cl3A	1.675 (12)
C17—C18	1.384 (3)	C22A—Cl4A	1.799 (12)
C17—C16	1.388 (3)	C22A—Cl4A^{i}	1.876 (15)
C17—H17	0.9500	C22A—H22C	1.011 (9)
C16—C15	1.391 (3)	C22A—H22D	1.001 (9)
C16—H16	0.9500	Cl4A—Cl4A^{i}	1.183 (10)
C15—C20	1.391 (3)	Cl3A—Cl3A^{i}	2.22 (4)
C18—C19	1.381 (3)		

C1—N1—C2	122.67 (15)	C105—C104—H104	119.5
C1—N1—C12	118.44 (16)	C106—C105—C104	119.89 (18)
C2—N1—C12	118.22 (16)	C106—C105—H105	120.1
O1—C1—N1	122.82 (16)	C104—C105—H105	120.1
O1—C1—C11	121.20 (16)	C105—C106—C107	120.20 (18)
N1—C1—C11	115.96 (14)	C105—C106—H106	119.9
C8—N2—C9	116.80 (16)	C107—C106—H106	119.9
C3—C2—C7	120.16 (17)	C106—C107—C102	119.89 (17)
C3—C2—N1	120.69 (17)	C106—C107—C108	121.33 (16)
C7—C2—N1	119.12 (16)	C102—C107—C108	118.78 (15)
C13—N3—C11	120.39 (14)	N6—C108—C107	118.96 (15)
C13—N3—C10	120.42 (15)	N6—C108—C111	127.97 (16)
C11—N3—C10	116.41 (14)	C107—C108—C111	112.94 (14)
C4—C3—C2	119.48 (18)	N6—C109—C110	112.22 (15)
C4—C3—H3	120.3	N6—C109—H10C	109.2
C2—C3—H3	120.3	C110—C109—H10C	109.2
C13—N4—H4A	114.9 (15)	N6—C109—H10D	109.2
C13—N4—H4B	121.8 (18)	C110—C109—H10D	109.2
H4A—N4—H4B	119 (2)	H10C—C109—H10D	107.9
C3—C4—C5	120.84 (19)	N7—C110—C109	109.04 (15)
C3—C4—H4	119.6	N7—C110—H11A	109.9

C5—C4—H4	119.6	C109—C110—H11A	109.9
C101—N5—C102	122.35 (15)	N7—C110—H11B	109.9
C101—N5—C112	117.74 (15)	C109—C110—H11B	109.9
C102—N5—C112	119.67 (15)	H11A—C110—H11B	108.3
C6—C5—C4	119.31 (19)	N7—C111—C108	111.07 (13)
C6—C5—H5	120.3	N7—C111—C101	109.63 (13)
C4—C5—H5	120.3	C108—C111—C101	108.03 (13)
C108—N6—C109	115.97 (15)	N7—C111—C114	112.52 (13)
C5—C6—C7	121.12 (19)	C108—C111—C114	106.11 (13)
C5—C6—H6	119.4	C101—C111—C114	109.33 (13)
C7—C6—H6	119.4	N5—C112—H11C	109.5
C113—N7—C111	121.24 (14)	N5—C112—H11D	109.5
C113—N7—C110	120.73 (14)	H11C—C112—H11D	109.5
C111—N7—C110	115.88 (14)	N5—C112—H11E	109.5
C6—C7—C2	119.03 (17)	H11C—C112—H11E	109.5
C6—C7—C8	121.70 (16)	H11D—C112—H11E	109.5
C2—C7—C8	119.24 (16)	O4—C113—N8	120.98 (17)
C113—N8—H8A	115.6 (17)	O4—C113—N7	123.45 (16)
C113—N8—H8B	116.9 (16)	N8—C113—N7	115.36 (16)
H8A—N8—H8B	117 (2)	C115—C114—C111	112.53 (13)
N2—C8—C7	119.61 (16)	C115—C114—H11F	109.1
N2—C8—C11	127.62 (17)	C111—C114—H11F	109.1
C7—C8—C11	112.53 (14)	C115—C114—H11G	109.1
N2—C9—C10	112.45 (16)	C111—C114—H11G	109.1
N2—C9—H9A	109.1	H11F—C114—H11G	107.8
C10—C9—H9A	109.1	C120—C115—C116	117.81 (17)
N2—C9—H9B	109.1	C120—C115—C114	120.78 (16)
C10—C9—H9B	109.1	C116—C115—C114	121.41 (16)
H9A—C9—H9B	107.8	C117—C116—C115	121.07 (18)
N3—C10—C9	109.91 (15)	C117—C116—H116	119.5
N3—C10—H10A	109.7	C115—C116—H116	119.5
C9—C10—H10A	109.7	C118—C117—C116	120.15 (19)
N3—C10—H10B	109.7	C118—C117—H117	119.9
C9—C10—H10B	109.7	C116—C117—H117	119.9
H10A—C10—H10B	108.2	C119—C118—C117	119.68 (19)
N3—C11—C8	111.49 (13)	C119—C118—H118	120.2
N3—C11—C1	109.79 (13)	C117—C118—H118	120.2
C8—C11—C1	106.80 (14)	C118—C119—C120	120.2 (2)
N3—C11—C14	112.20 (14)	C118—C119—H119	119.9
C8—C11—C14	106.97 (13)	C120—C119—H119	119.9
C1—C11—C14	109.40 (13)	C115—C120—C119	121.11 (19)

O2—C13—N4	120.00 (17)
O2—C13—N3	123.24 (16)
N4—C13—N3	116.61 (17)
N1—C12—H12A	109.5
N1—C12—H12B	109.5
H12A—C12—H12B	109.5
N1—C12—H12C	109.5
H12A—C12—H12C	109.5
H12B—C12—H12C	109.5
C15—C14—C11	112.02 (14)
C15—C14—H14A	109.2
C11—C14—H14A	109.2
C15—C14—H14B	109.2
C11—C14—H14B	109.2
H14A—C14—H14B	107.9
C18—C17—C16	120.02 (19)
C18—C17—H17	120.0
C16—C17—H17	120.0
C17—C16—C15	121.21 (18)
C17—C16—H16	119.4
C15—C16—H16	119.4
C16—C15—C20	118.19 (17)
C16—C15—C14	120.85 (16)
C20—C15—C14	120.95 (16)
C19—C18—C17	119.70 (18)
C19—C18—H18	120.2
C17—C18—H18	120.2
C18—C19—C20	120.1 (2)
C18—C19—H19	119.9
C20—C19—H19	119.9
C15—C20—C19	120.73 (19)
C15—C20—H20	119.6
C19—C20—H20	119.6
Cl2—C21—Cl1	111.09 (17)
Cl2—C21—H21A	109.4
Cl1—C21—H21A	109.4
Cl2—C21—H21B	109.4
Cl1—C21—H21B	109.4
H21A—C21—H21B	108.0
O3—C101—N5	121.62 (15)
O3—C101—C111	120.34 (15)
C115—C120—H120	119.4
C119—C120—H120	119.4
C22^{i}—C22—Cl3^{i}	102.1 (18)
C22^{i}—C22—Cl4	71.1 (5)
Cl3^{i}—C22—Cl4	149.9 (18)
C22^{i}—C22—Cl3	39.0 (9)
Cl3^{i}—C22—Cl3	82 (2)
Cl4—C22—Cl3	105.1 (7)
C22^{i}—C22—H22A	104.3 (17)
Cl3^{i}—C22—H22A	41 (2)
Cl4—C22—H22A	110.5 (12)
Cl3—C22—H22A	109.4 (12)
C22^{i}—C22—H22B	140.1 (18)
Cl3^{i}—C22—H22B	93 (2)
Cl4—C22—H22B	110.8 (12)
Cl3—C22—H22B	109.5 (12)
H22A—C22—H22B	111.3 (13)
C22—Cl4—C22^{i}	37.9 (10)
C22^{i}—Cl3—C22	38.9 (14)
C22^{i}—Cl3—Cl3^{i}	62.8 (17)
C22—Cl3—Cl3^{i}	34.9 (8)
C22^{i}—Cl3—H22A^{i}	59.0 (10)
C22—Cl3—H22A^{i}	71 (2)
Cl3^{i}—Cl3—H22A^{i}	106 (3)
C22^{i}—Cl3—H22D^{i}	90.2 (18)
C22—Cl3—H22D^{i}	71.6 (10)
Cl3^{i}—Cl3—H22D^{i}	93.0 (16)
H22A^{i}—Cl3—H22D^{i}	48 (3)
C22A^{i}—C22A—Cl3A^{i}	65.7 (11)
C22A^{i}—C22A—Cl3A	57.8 (9)
Cl3A^{i}—C22A—Cl3A	86.6 (19)
C22A^{i}—C22A—Cl4A	68.0 (8)
Cl3A^{i}—C22A—Cl4A	96.5 (11)
Cl3A—C22A—Cl4A	118.4 (7)
C22A^{i}—C22A—H22C	153.1 (17)
Cl3A^{i}—C22A—H22C	141 (2)
Cl3A—C22A—H22C	111.6 (11)
Cl4A—C22A—H22C	103.3 (11)
Cl4A^{i}—C22A—H22C	94.4 (15)
C22A^{i}—C22A—H22D	101.4 (17)
Cl3A^{i}—C22A—H22D	37 (2)

N5—C101—C111	118.04 (14)	Cl3A—C22A—H22D	112.6 (11)
C107—C102—C103	119.53 (17)	Cl4A—C22A—H22D	104.2 (11)
C107—C102—N5	119.50 (15)	Cl4A^{i}—C22A—H22D	140.9 (13)
C103—C102—N5	120.95 (16)	H22C—C22A—H22D	105.5 (12)
C104—C103—C102	119.53 (18)	Cl4A^{i}—Cl4A—C22A	74.8 (7)
C104—C103—H103	120.2	C22A^{i}—Cl3A—C22A	56.5 (12)
C102—C103—H103	120.2	C22A^{i}—Cl3A—Cl3A^{i}	49.0 (11)
C103—C104—C105	120.90 (18)	C22A—Cl3A—Cl3A^{i}	44.5 (10)
C103—C104—H104	119.5	H22A^{i}—Cl3A—H22D^{i}	41 (3)

Symmetry code: (i) −*x*+1, *y*, −*z*+1/2.

Table S16: Hydrogen-bond geometry (Å, º) for **7b**.

D—H···*A*	*D*—H	H···*A*	*D*···*A*	*D*—H···*A*
N4—H4*A*···O3	0.96 (3)	2.51 (3)	3.240 (2)	132.6 (19)
N4—H4*B*···O4	0.86 (3)	2.14 (3)	2.847 (2)	139 (2)
C10—H10$^{A\cdots Cl4A}$b	0.99	2.88	3.675 (5)	138
C14—H14*A*···O2	0.99	2.25	2.876 (2)	120
C21—H21*A*···O2	0.99	2.59	3.476 (3)	148
C114—H11*G*···O4	0.99	2.34	2.899 (2)	115
N8—H8*A*···O3^{i}	0.82 (3)	2.25 (3)	3.051 (2)	164 (2)
N8—H8*B*···O2^{i}	0.86 (3)	2.17 (3)	3.020 (2)	170 (2)
C112—H11*C*···O1^{ii}	0.98	2.37	3.305 (2)	159
C110—H11*B*···O1^{i}	0.99	2.39	3.260 (2)	147

Symmetry codes: (i) −*x*+3/2, *y*+1/2, −*z*+1/2; (ii) −*x*+3/2, *y*−1/2, −*z*+1/2.

ESI-MS spectra of compounds 2, 3, 4, 5, 6, 7, 8

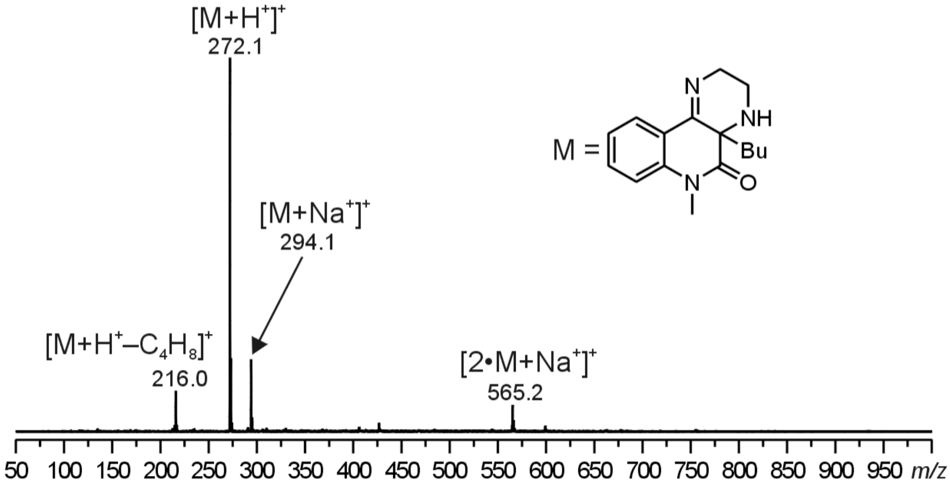

Figure S34: The positive-ion ESI mass spectra of compound **2a**. The assignments for observed signals are shown in the brackets.

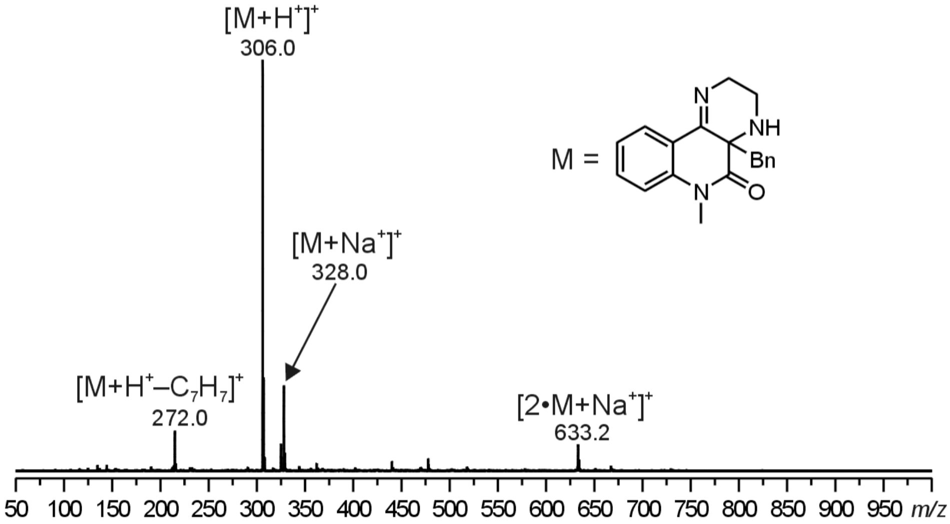

Figure S35: The positive-ion ESI mass spectra of compound **2b**. The assignments for observed signals are shown in the brackets.

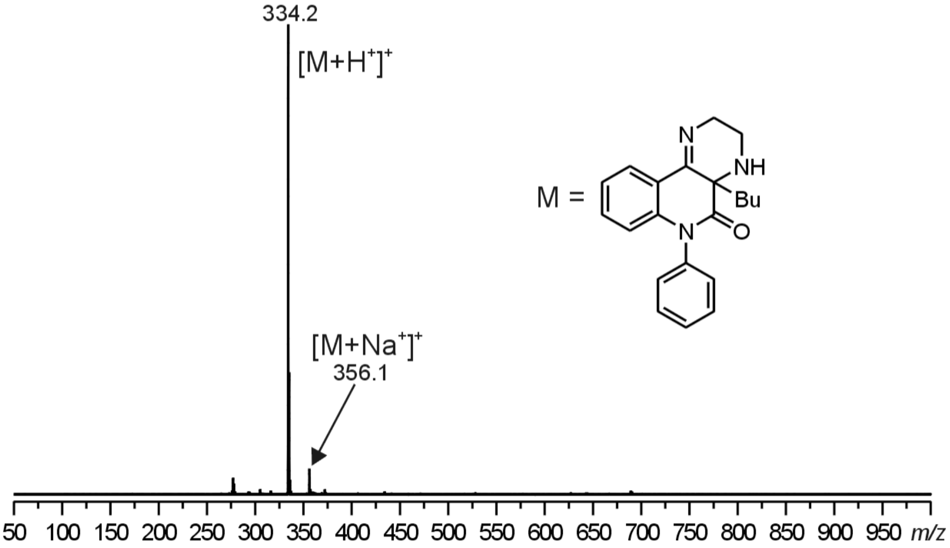

Figure S36: The positive-ion ESI mass spectra of compound **2d**. The assignments for observed signals are shown in the brackets.

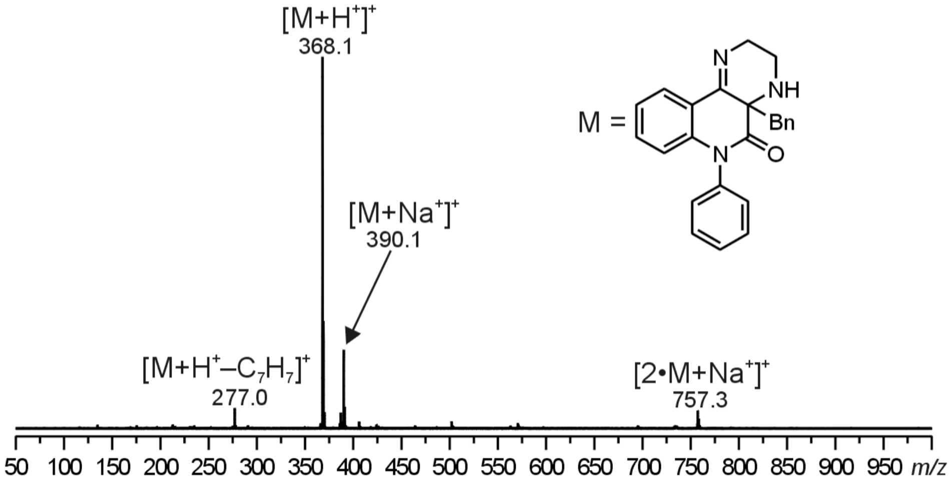

Figure S37: The positive-ion ESI mass spectra of compound **2e**. The assignments for observed signals are shown in the brackets.

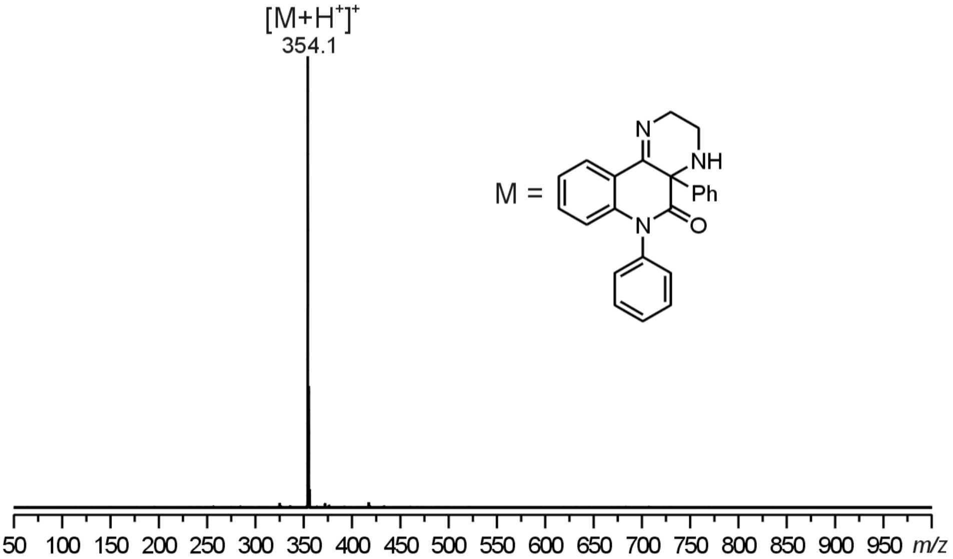

Figure S38: The positive-ion ESI mass spectra of compound **2f**. The assignments for observed signals are shown in the brackets.

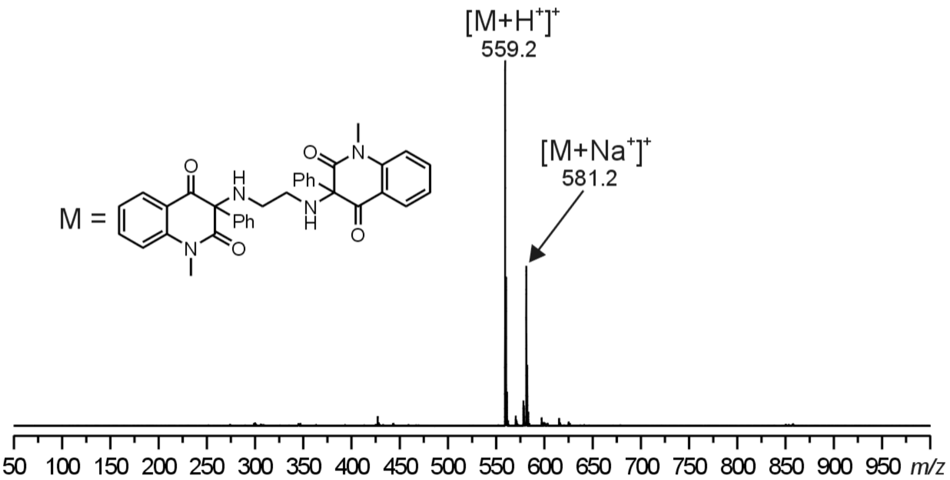

Figure S39: The positive-ion ESI mass spectra of compound **3c**. The assignments for observed signals are shown in the brackets.

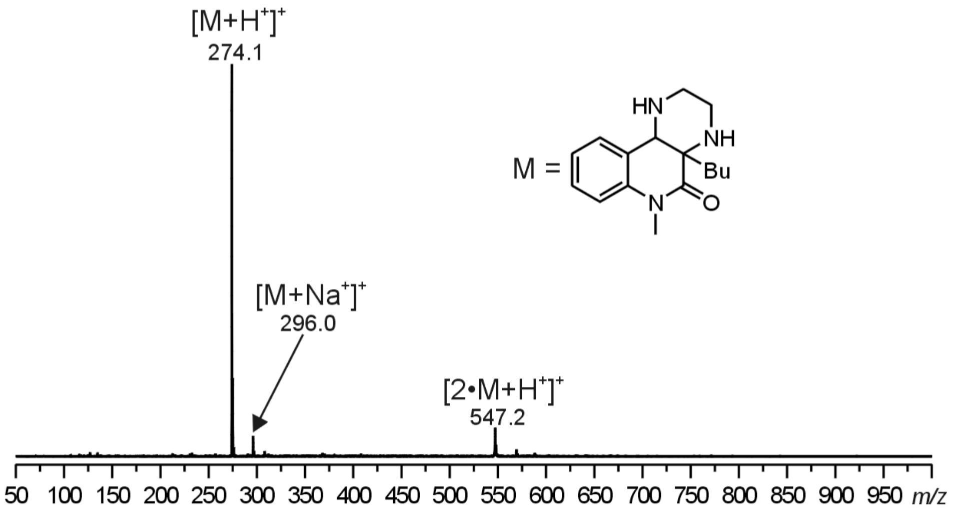

Figure S40: The positive-ion ESI mass spectra of compound **4a**. The assignments for observed signals are shown in the brackets.

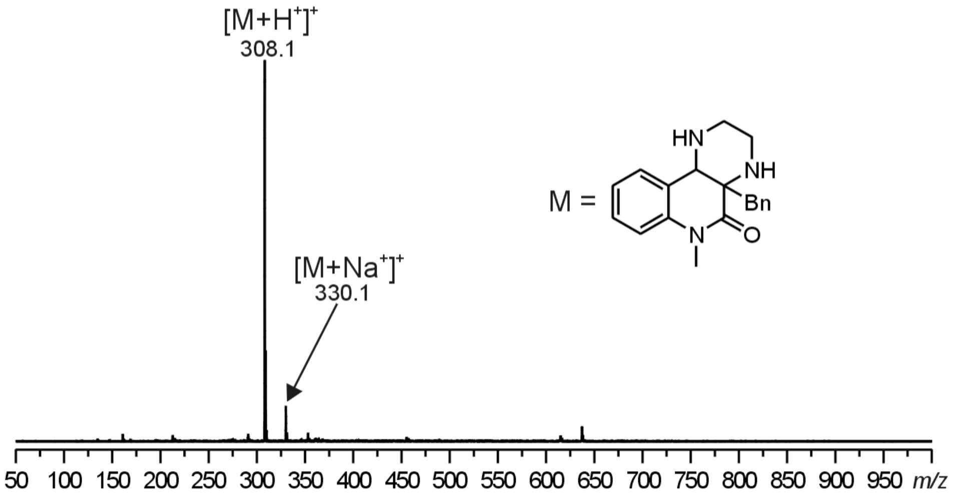

Figure S41: The positive-ion ESI mass spectra of compound **4b**. The assignments for observed signals are shown in the brackets.

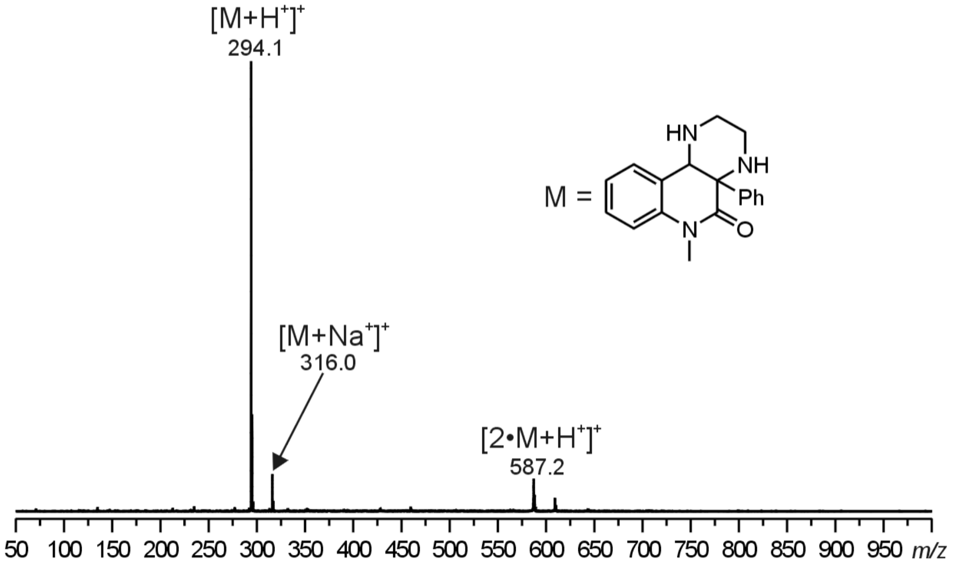

Figure S42: The positive-ion ESI mass spectra of compound **4c**. The assignments for observed signals are shown in the brackets.

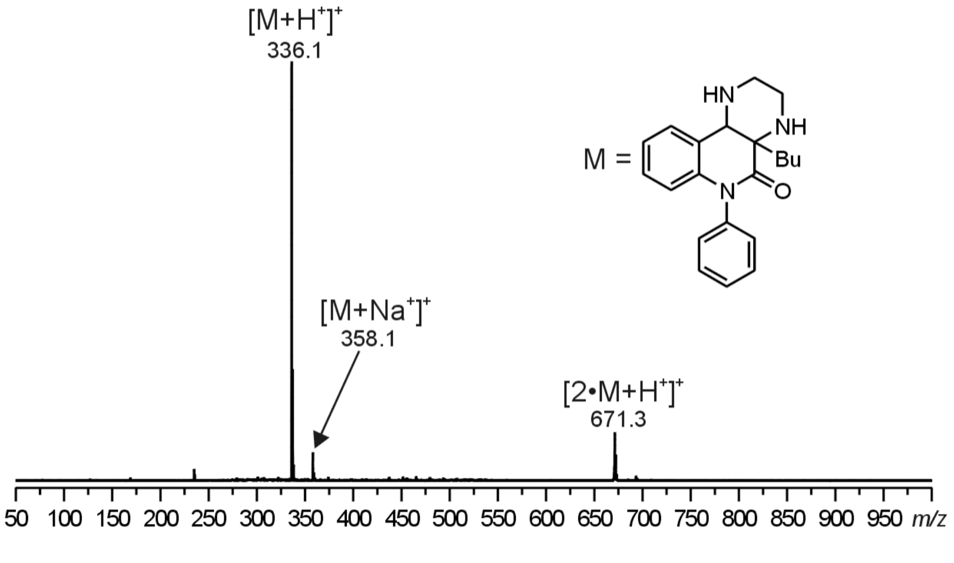

Figure S43: The positive-ion ESI mass spectra of compound **4d**. The assignments for observed signals are shown in the brackets.

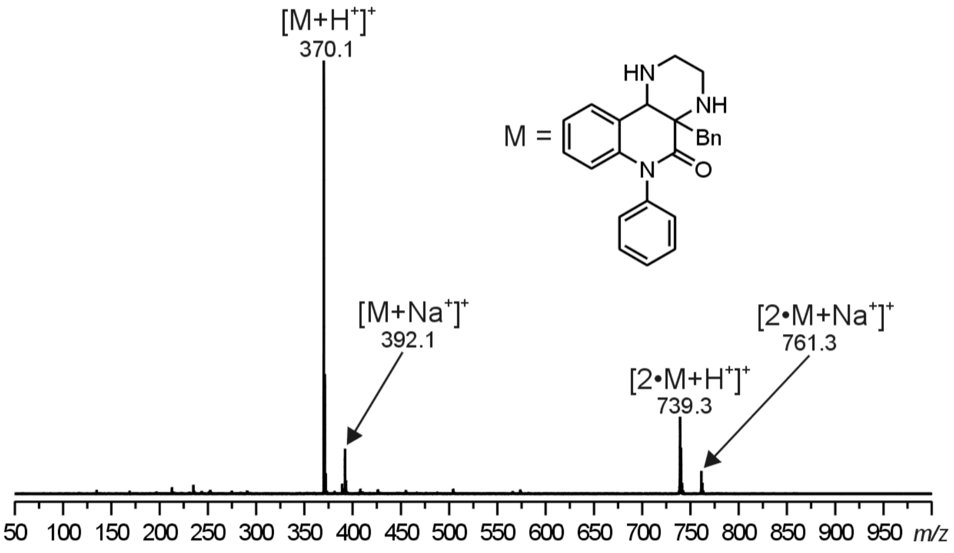

Figure S45: The positive-ion ESI mass spectra of compound **4e**. The assignments for observed signals are shown in the brackets.

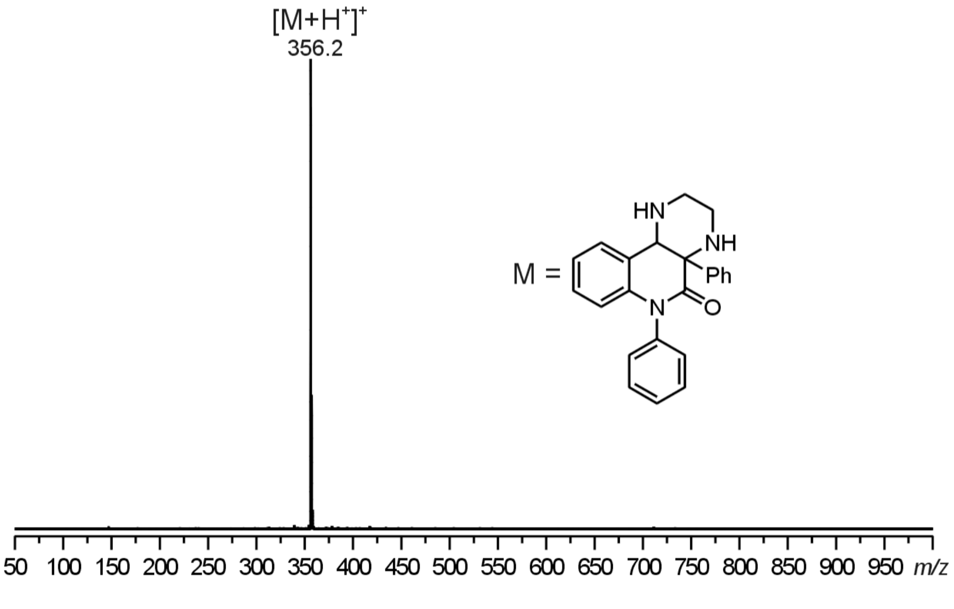

Figure S46: The positive-ion ESI mass spectra of compound **4f**. The assignments for observed signals are shown in the brackets.

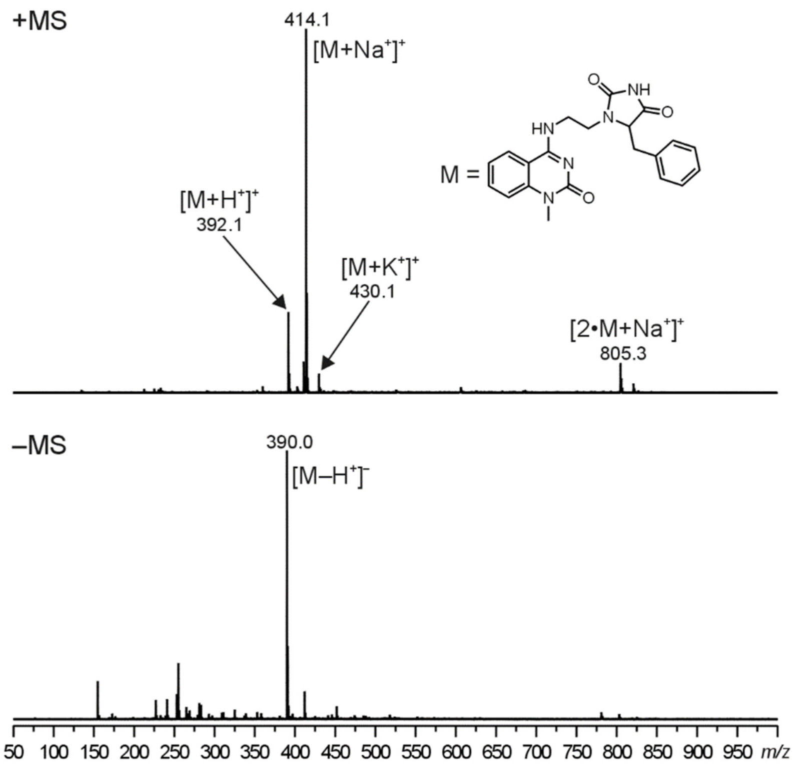

Figure S47: The positive and negative-ion ESI mass spectra of compound **5b**. The assignments for observed signals are shown in the brackets.

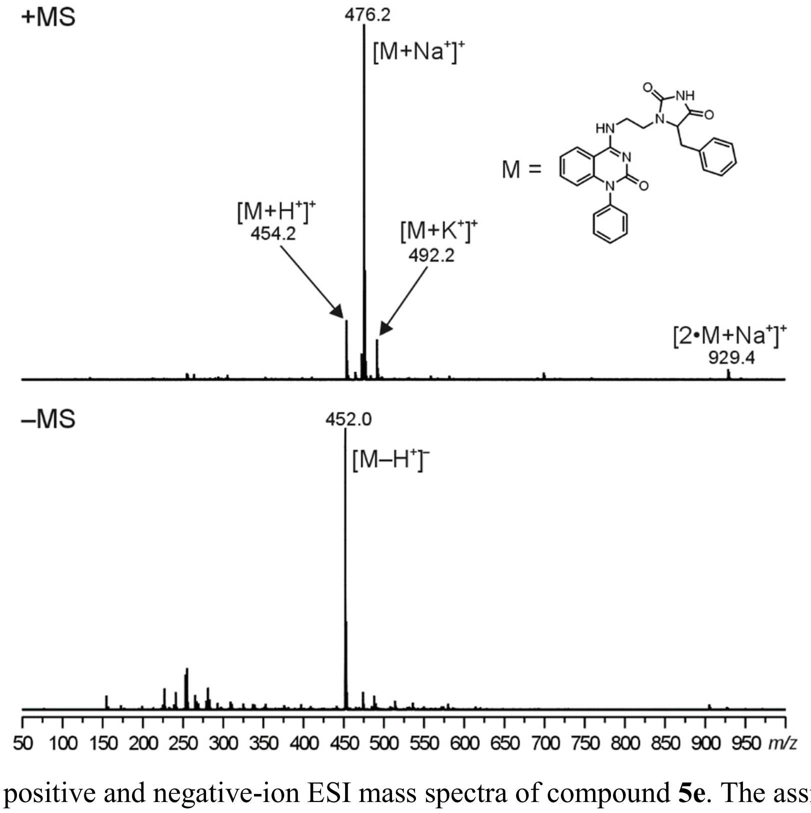

Figure S48: The positive and negative-ion ESI mass spectra of compound **5e**. The assignments for observed signals are shown in the brackets.

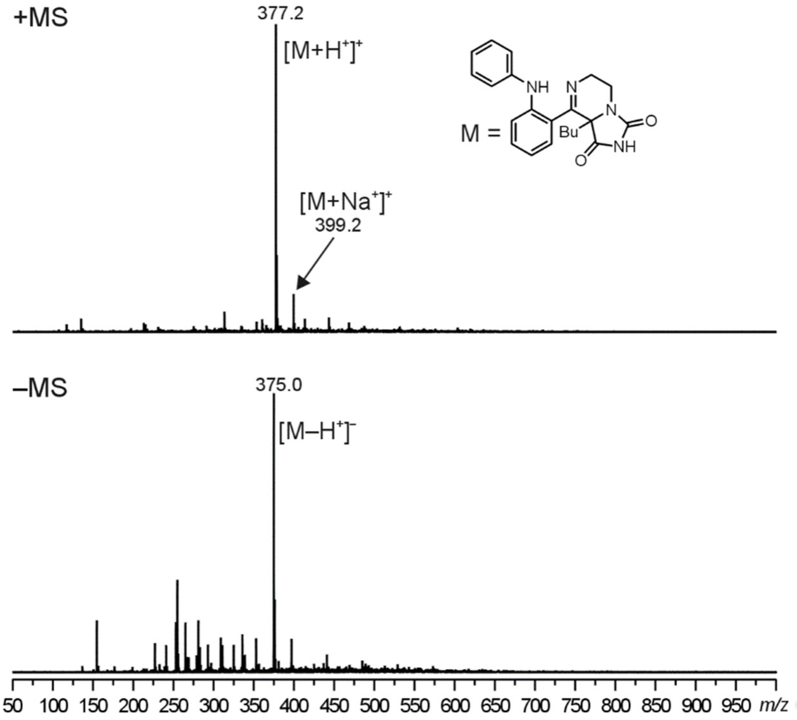

Figure S49: The positive and negative-ion ESI mass spectra of compound **6d**. The assignments for observed signals are shown in the brackets.

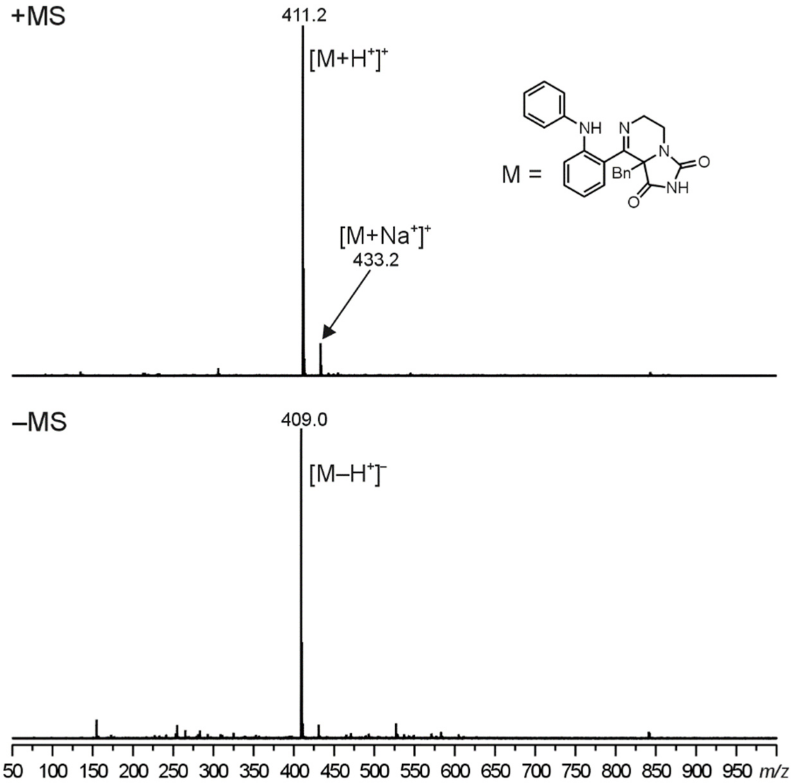

Figure S50: The positive and negative-ion ESI mass spectra of compound **6e**. The assignments for observed signals are shown in the brackets.

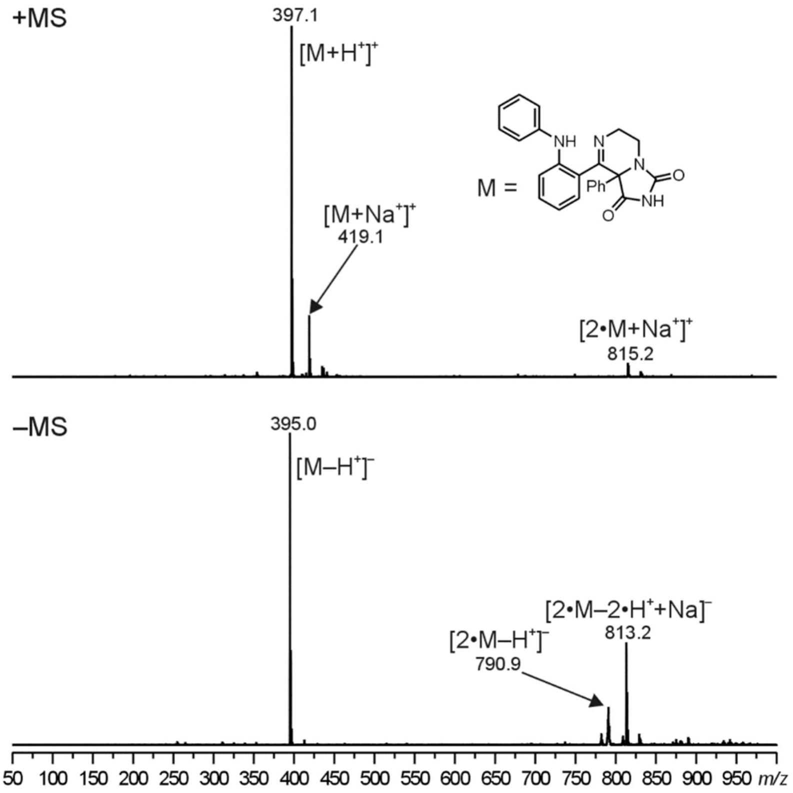

Figure S51: The positive and negative-ion ESI mass spectra of compound **6f**. The assignments for observed signals are shown in the brackets.

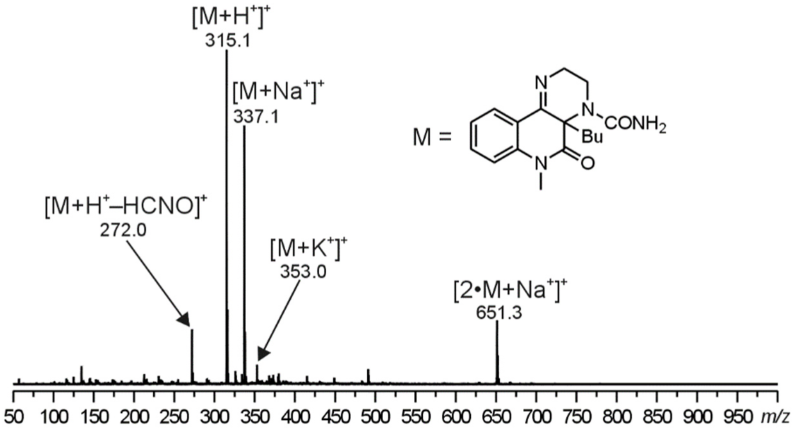

Figure S52: The positive -ion ESI mass spectra of compound **7a**. The assignments for observed signals are shown in the brackets.

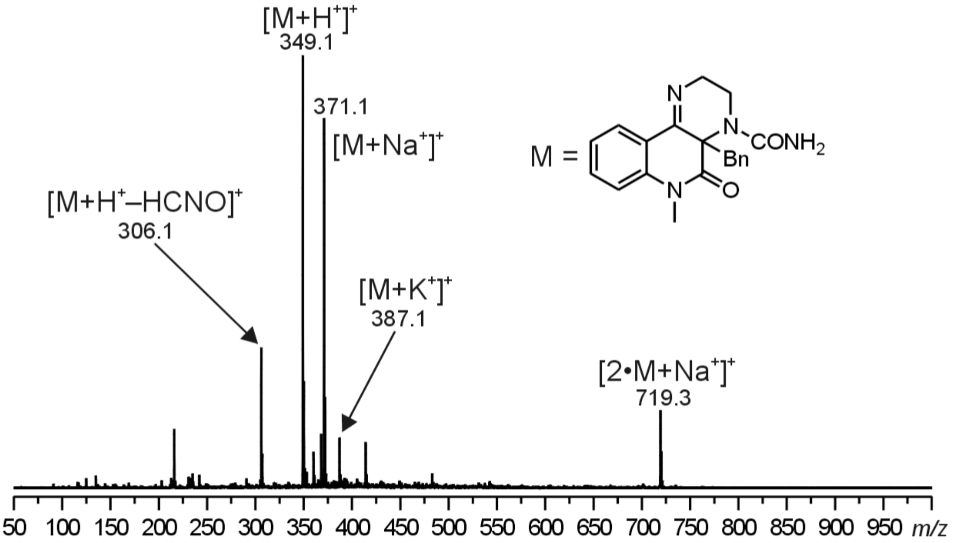

Figure S53: The positive -ion ESI mass spectra of compound **7b**. The assignments for observed signals are shown in the brackets.

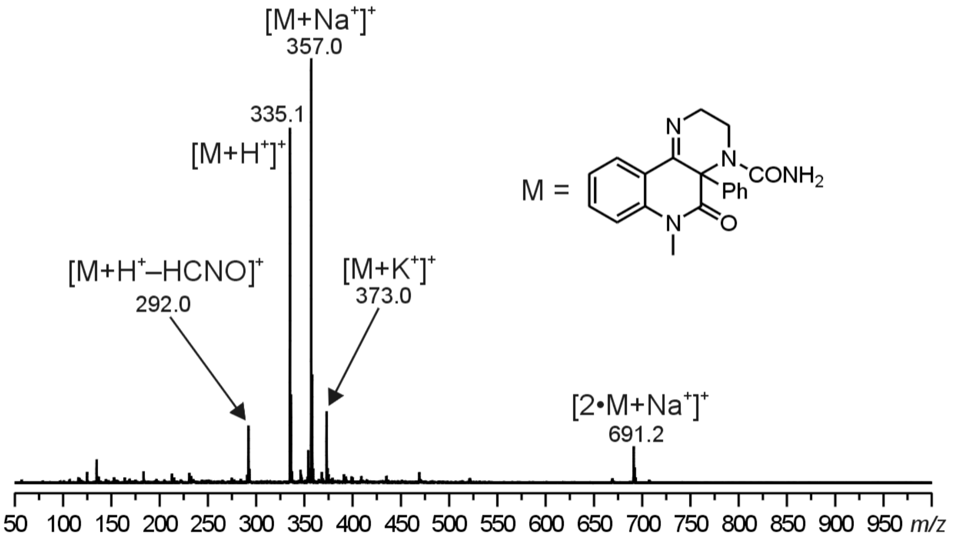

Figure S54: The positive -ion ESI mass spectra of compound **7c**. The assignments for observed signals are shown in the brackets.

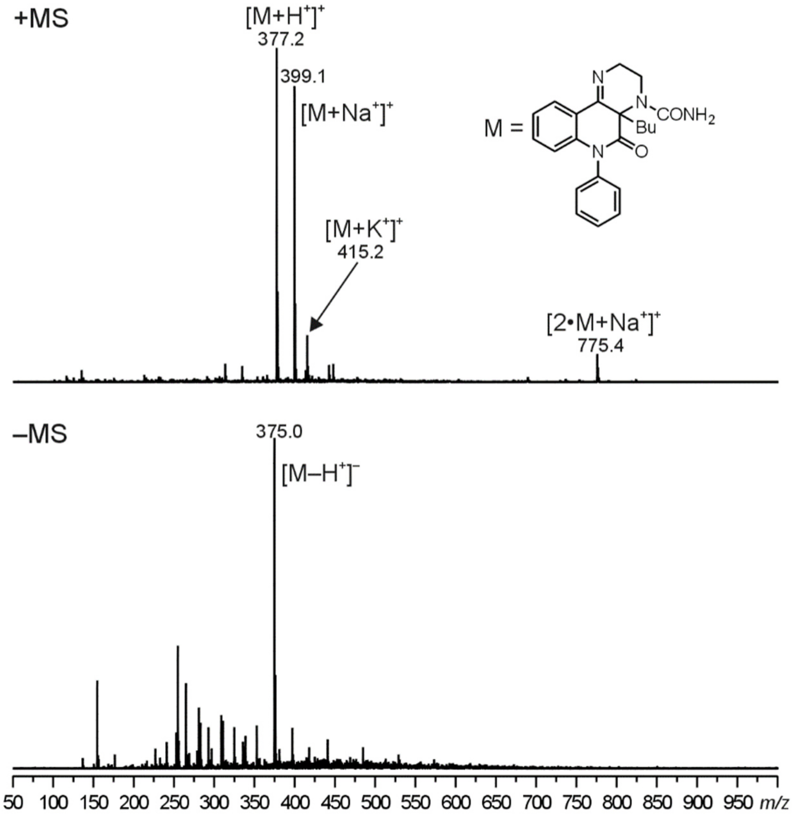

Figure S55: The positive and negative-ion ESI mass spectra of compound **7d**. The assignments for observed signals are shown in the brackets.

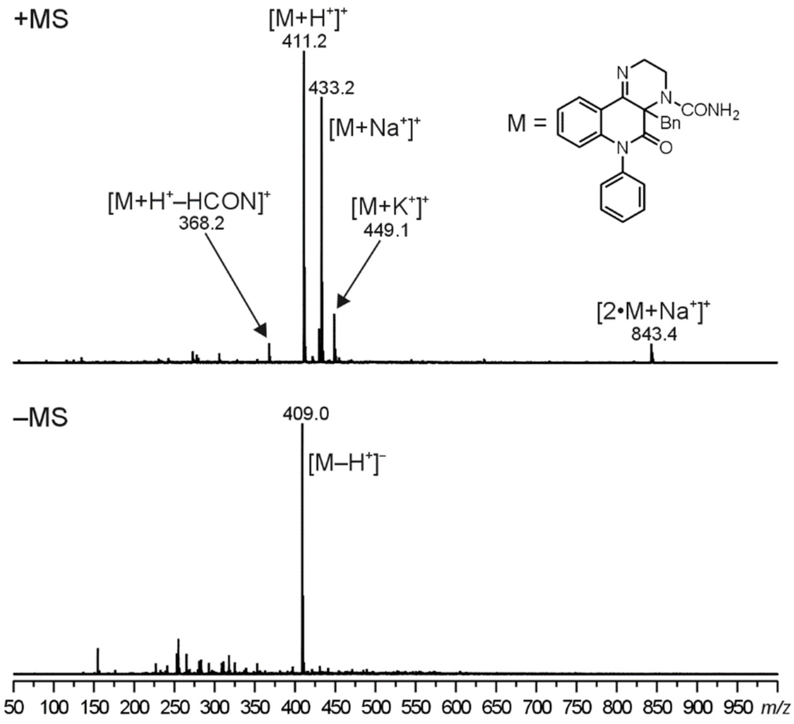

Figure S56: The positive and negative-ion ESI mass spectra of compound **7e**. The assignments for observed signals are shown in the brackets.

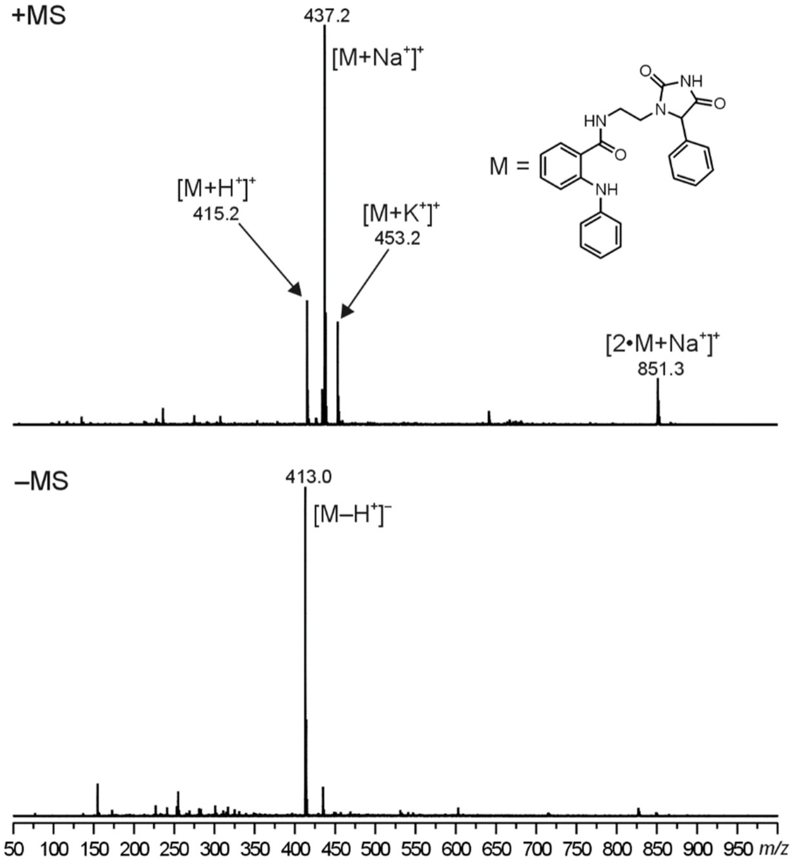

Figure S57: The positive and negative-ion ESI mass spectra of compound **8f**. The assignments for observed signals are shown in the brackets.
